# Molecular Recognition of the Catalytic Zinc(II) Ion in MMP-13: Structure-Based Evolution of an Allosteric Inhibitor to Dual Binding Mode Inhibitors with Improved Lipophilic Ligand Efficiencies

**DOI:** 10.3390/ijms17030314

**Published:** 2016-03-01

**Authors:** Thomas Fischer, Rainer Riedl

**Affiliations:** Center for Organic and Medicinal Chemistry, Institute of Chemistry and Biotechnology, Zurich University of Applied Sciences ZHAW, Einsiedlerstrasse 31, 8820 Wädenswil, Switzerland; thomas.fischer@zhaw.ch

**Keywords:** structure-based drug design, organic synthesis, matrix metalloproteinase inhibitors, molecular recognition, weak zinc binders, lipophilic ligand efficiency

## Abstract

Matrix metalloproteinases (MMPs) are a class of zinc dependent endopeptidases which play a crucial role in a multitude of severe diseases such as cancer and osteoarthritis. We employed MMP-13 as the target enzyme for the structure-based design and synthesis of inhibitors able to recognize the catalytic zinc ion in addition to an allosteric binding site in order to increase the affinity of the ligand. Guided by molecular modeling, we optimized an initial allosteric inhibitor by addition of linker fragments and weak zinc binders for recognition of the catalytic center. Furthermore we improved the lipophilic ligand efficiency (LLE) of the initial inhibitor by adding appropriate zinc binding fragments to lower the clogP values of the inhibitors, while maintaining their potency. All synthesized inhibitors showed elevated affinity compared to the initial hit, also most of the novel inhibitors displayed better LLE. Derivatives with carboxylic acids as the zinc binding fragments turned out to be the most potent inhibitors (compound **3** (ZHAWOC5077): IC_50_ = 134 nM) whereas acyl sulfonamides showed the best lipophilic ligand efficiencies (compound **18** (ZHAWOC5135): LLE = 2.91).

## 1. Introduction

As the most efficient contributor to degeneration of type II collagen within its superfamily of zinc-dependant endopeptidases, matrix metalloproteinase-13 (MMP-13) is a highly validated target for the treatment of diseases based on tissue degradation [1,2,3,4,5]. Unregulated MMP activity can lead to a variety of diseases such as tumor growth and metastasis, periodontal disease and osteoarthritis (OA) [6,7,8,9,10,11,12].

Initial success in developing inhibitors against MMP-13 was achieved over a decade ago by modifying the natural substrate close to the scissile amide bond. By implementing structural elements that cannot be hydrolyzed by the enzyme, peptidomimetics were obtained [13]. Potent inhibitors resulted by adding strong zinc binding groups such as hydroxamic acid to interact with the catalytic domain [14,15]. Those inhibitors lacked sufficient selectivity due to high structural similarity among the various MMPs. Because of toxicity and severe side effects arising from scarce selectivity no compound with strong zinc chelating groups such as hydroxamic acids has survived clinical trials so far [16,17]. 

Later generations of MMP-13 inhibitors did not bind to the catalytic zinc, but buried themselves deeper in the S_1_’ pocket, occupying the specificity loop leading to inhibitors that combine high potency with appealing selectivity profiles [18,19,20,21,22,23,24,25]. Adding weak zinc binding groups to those allosteric inhibitors can lead to selective inhibitors with higher potency due to a dual binding mode (allosteric pocket and catalytic center) [26]. 

**Figure 1:**
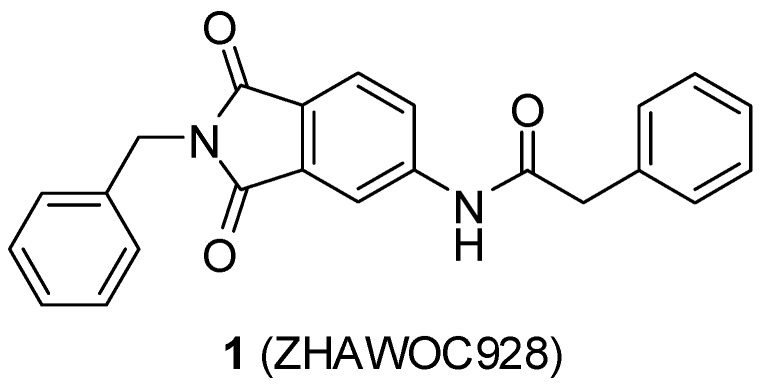
Chemical structure of the previously identified inhibitor **1**.

A previous study conducted in our laboratory led to the identification of compound **1** (Figure 1) with a lipophilic ligand efficiency (LLE = 1.07) which inhibits MMP-13 showing an IC_50_ value of 9.8 μM without chelating the catalytic zinc ion [27]. 

In this article we present a structure-based design approach to modify the allosteric MMP-13 inhibitor **1** to increase its potency by improving the molecular recognition between the enzyme and its ligand. This could be achieved by the introduction of a weak zinc binding fragment in order to interact with the catalytic center. Furthermore we enhanced the lipophilic ligand efficiency of the novel compounds compared to the allosteric inhibitor.

## 2. Results

### 2.1. Molecular Modeling

**Figure 2:**
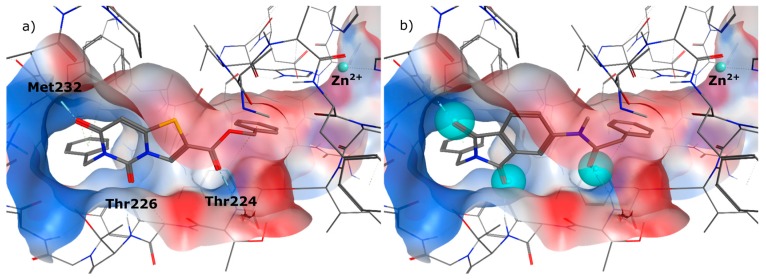

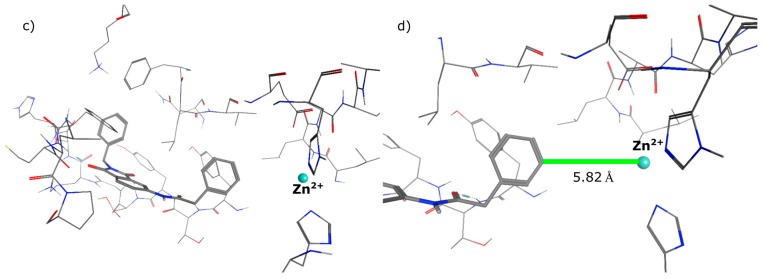
(**a**) Co-crystal structure PDB 2OW9; (**b**) compound **1** docked to PDB 2OW9 with the pharmacophore being fulfilled; (**c**) possibility of attaching a weak zinc binder in meta position; (**d**) distance between ligand and zinc ion.

In the first step of the design process we had a close look on the binding mode of **1** in the allosteric binding pocket of MMP-13. We used the crystal structure PDB 2OW9 (Figure 2a) [24] to dock the molecule into the cavity defining a pharmacophore query to maintain the three hydrogen bonds to the backbone amino acids Thr224, Thr226 and Met232, formed by the oxygen atoms of the phthalimide scaffold (Figure 2b). It turned out that a zinc binding group could be attached to the molecule, favourably in meta position, to interact with the catalytic zinc ion (Figure 2c). As the distance that has to be overcome added up to more than 5 Å (Figure 2d) we decided to implement a linker consisting of four and more heavy atoms between the aromatic ring system and the chelating group.

**Figure 3:**
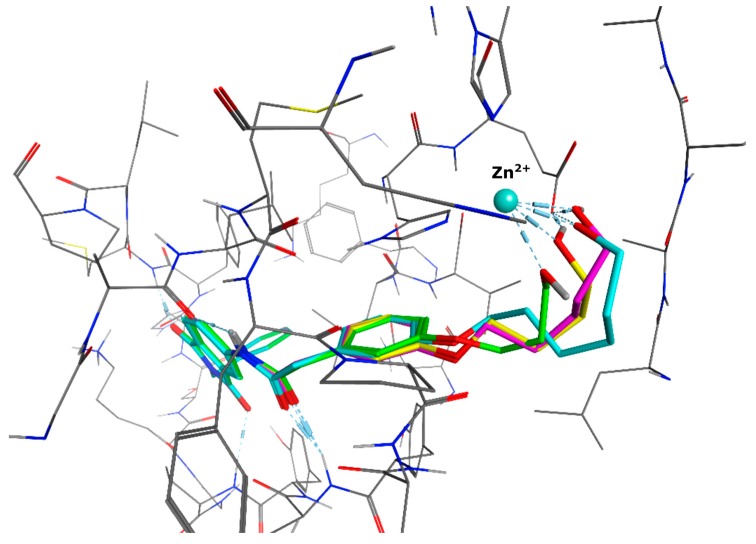
Docking poses of four molecules with different linker lengths and the zinc-chelating hydroxy group (green: 3 CH_2_, yellow: 4 CH_2_, magenta: 5 CH_2_, cyan: 6 CH_2_).

At the meta position of the aromatic ring an oxygen was added for synthetic reasons, followed by aliphatic linker chains between 3–6 carbon atoms in length as well as a terminal hydroxy group. The alcohol head group was implemented as a generic zinc chelator of small size to evaluate the optimal linker length (Figure 3).

**Figure 4:**
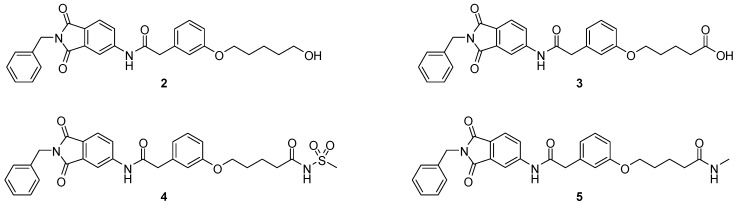
Structural proposals for extended molecules to overcome the distance to the zinc ion, including different weak zinc binders.

We decided to focus on chain lengths of 4 and 5 carbon atoms (Figure 3, yellow and magenta) as they showed lower root mean square deviation (RMSD) values (1.16 yellow and 1.35 magenta) than the other two linkers (1.56 green and 2.57 cyan). This indicates better quality of the docking poses [28]. In addition, visual inspection of the docking poses indicated that chain lengths of 4 and 5 carbon atoms seemed to cover the distance more efficiently than the other two linkers. Subsequently we examined different head groups as potential zinc chelators. Therefore we modified structure **2** by implementing a carboxylic acid (**3**), an acyl sulfonamide (**4**) and an amide (**5**) to obtain a set of ligands for which the recognition of the catalytic zinc should increase their affinity to the protein and therefore enhance the inhibitors potency (Figure 4) [29,30].

**Figure 5:**
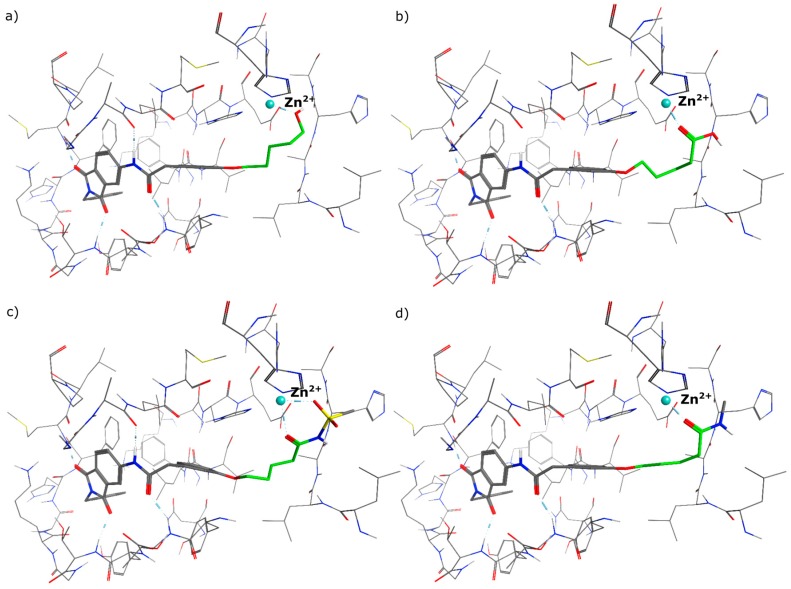
Highest ranked docking pose of (**a**) alcohol **2**; (**b**) carboxylic acid **3**; (**c**) acyl sulfonamide **4**; (**d**) amide **5**, with the linker emphasized (green) and the head group showing the anticipated interaction with the zinc.

For the purpose of this evaluation we executed docking experiments applying the identical pharmacophore as for the previously docked structure **1** (Figure 5a–d).

**Figure 6:**
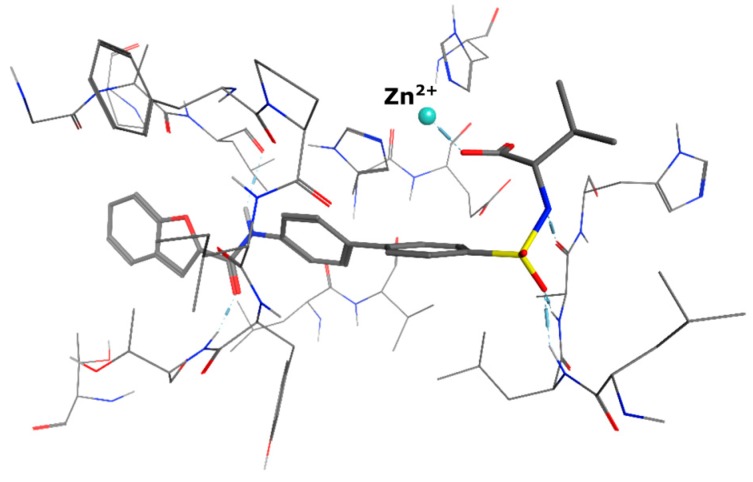
Co-crystal structure of a carboxylic acid interacting with the zinc ion in the active site of MMP-13 involving only one of the two oxygen atoms.

All of the four examined weak zinc binding head groups showed the postulated molecular recognition of the metal ion. The alcohol and the acid as well as the amide exhibit monodentate binding, whereas the acyl sulfonamide acts as a bidentate chelator. According to our modeling experiments, the carboxylic acid interacts as a monodentate ligand. This corresponds well with the reported binding mode of carboxylic acids in the active site of MMP-13 (Figure 6, crystal structure PDB 1ZTQ [31]).

The designed compounds **2**–**5** displayed better scoring values (in the range of −18) compared to **1** (−12.8). In consideration of the modeling results described above, we decided to synthesize a small library of this type of molecules.

### 2.2. Chemistry

**Figure 7:**
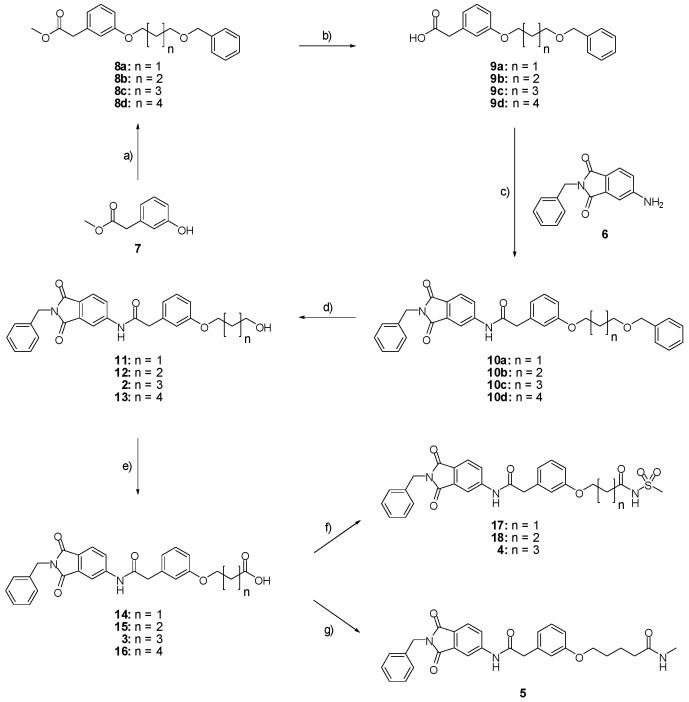
Synthesis of compounds **2**–**18**. (**a**) Benzyl-n-bromoalkylether, Cs_2_CO_3_, DMF, RT, 18 h, **8a**: 80%, **8b**: 85%, **8c**: 83%, **8d**: 84%; (**b**) KOH 10% in H_2_O, methanol, RT, 30 min., **9a**: 89%, **9b**: 92%, **9c**: 100%, **9d**: 94%; (**c**) SOCl_2_, DIPEA, THF, RT, 2 h, **10a**: 74%, **10b**: 64%, **10c**: 66%, **10d**: 55%; (**d**) Pd/C 10%, ethanol, RT, 2 h, **11**: 57%, **12**: 48%, **2**: 83%, **13**: 77%; (**e**) TEMPO, sodium phosphate 0.67 M in H_2_O pH 6.7, NaClO_2_, NaOCl, ACN, 40 °C, 4 h, **14**: 51%, **15**: 84%, **3**: 53%, **16**: 87%; (**f**) EDC·HCl, methanesulfonamide, DMAP, DCM, 0 °C, 20 min., RT, 12 h, **17**: 19%, **18**: 26%, **4**: 41%; (**g**) DIPEA, HATU, methylamine 2 M in THF, THF, RT, 12 h, **5**: 39%.

Driven by the positive results of our computational design approach towards molecular recognition of the catalytic zinc ion, we synthesized compounds **2**–**5** according to the synthetic route summarized in Figure 7. The benzylated aminophthalimide derivative **6** was prepared according to a previously described procedure [27]. The acid **9** was obtained by saponification of **8** which was prepared by the nucleophilic substitution of **7** on benzyl-n-bromoalkylether. An amide coupling between the alkylated aminophthalimide **6** and the acid **9** led to compound **10** which could be hydrogenated to obtain the alcohol **2**. By subsequent oxidation of the alcohol **2** the acid **3** was formed and finally converted to the compounds **4** and **5** by amide bond formation.

### 2.3. Biological Assays

Consecutively the synthesized compounds were tested for their inhibition activity against MMP-13 in *in vitro* assays. The values are averaged over triplicate determinations (Table 1).

The carboxylic acids **3** and **16** turned out to be the most potent inhibitors within this series (IC_50_: 134 nM (**3**), 280 nM (**16**)). The acyl sulfonamides **4** and **18** show some of the best lipophilic ligand efficiencies (LLE: 2.53 (**4**), 2.91 (**18**)).

## 3. Discussion

We modified the allosteric inhibitor **1** of MMP-13 to increase its potency against the target enzyme and to elevate the LLE of the initial inhibitor. Increased potency could be achieved by the addition of a zinc binding fragment to the inhibitor. The carboxylic acid turned out to be the most potent option for the molecular recognition of the catalytic zinc(II) ion. The new inhibitors combine allosteric interaction with the recognition of the catalytic center to yield dual binding mode inhibitors. Compound **3** showed the highest affinity with an IC_50_ value of 134 nM. Increased LLE values were achieved by the implementation of acyl sulfonamide fragments to **1** which lowered the clogP value of the respective inhibitor. By comparing the acyl sulfonamide **18** with its carboxylic acid counterpart **15** one observes that the LLE improves from 2.16 to 2.91, which is in the range of lead compounds, while the IC_50_ values are comparable. Therefore acyl sulfonamides are good bioisosteres for substituting carboxylic acids as zinc binding functional groups if an increase of the LLE of MMP inhibitors is intended. 

The results of the series with carboxylic acids as the zinc binding group clearly show that the selection of excessively short linkers leads to a loss in potency. Also linkers with six heavy atoms between the allosteric fragment and the zinc-recognizing acid cause diminished inhibition, leading to an optimal linker length of five heavy atoms. These findings harmonize with our modeling results as we observed better geometrical matches for linkers with medium lengths.

It should be mentioned that the critical observation of the generated docking poses with respect to binding angles and torsions was absolutely mandatory for the successful outcome of this study. The corresponding scoring values can only be compared for sets of molecules with comparable binding modes, but even then it is safer to examine the poses visually instead of using the scoring values for prioritizing compounds for synthesis.

## 4. Materials and Methods 

### 4.1. General

All NMR spectra were recorded on a Bruker AVANCE III HD 500 One Bay spectrometer with a magnetic field of 11.75 T. For ^1^H NMR spectra a frequency of 500 MHz resulted. Chemical shifts are reported in ppm from tetramethylsilane as internal standard. Data are reported as follows: chemical shift, integration, multiplicity (s = singlet, d = doublet, t = triplet, q = quartet, quint. = quintet, br. = broad, m = multiplet), coupling constants (Hz). For ^13^C NMR spectra a frequency of 125 MHz resulted. Chemical shifts are reported in ppm from tetramethylsilane as internal standard. The multiplicities of the signals were determined by DEPT Measurements. High-resolution mass spectrometry was performed on an Agilent Technologies 6530 Q-TOF. NMR spectra, HRMS spectra and IC_50_ curves can be found in the supplementary materials.

### 4.2. Chemistry

All reagents and solvents used in the synthesis were purchased from Sigma Aldrich (Buchs, Switzerland) or TCI (Zwijndrecht, Belgium) and used as received. Solvents were stored over molecular sieves 4 Å.

*Methyl 2-(3-{[5-(benzyloxy)pentyl]oxy}phenyl)acetate* (**8c**; ZHAWOC4927): under an argon atmosphere, methyl 2-(3-hydroxyphenyl)acetate (**7**) (2.5 g, 15.05 mmol) and caesium carbonate (9.81 g, 30.11 mmol) were suspended in dimethylformamide (100 mL), the mixture was stirred at ambient temperature for 2 h. Benzyl-5-bromoamylether (4.26 g, 16.56 mmol) was added and it was stirred at ambient temperature for further 12 h. Water (250 mL) and ethyl acetate (250 mL) was added and the resulting phases separated. The organic phase was dried over sodium sulfate and concentrated in vacuum. Purification by chromatography on silica gel (Gradient: 0%–100% ethyl acetate in cyclohexane) afforded the title compound **8c** as a white solid (4.30 g, 83% yield): ^1^H-NMR (500 MHz, [D_6_]DMSO, 25 °C, TMS): δ = 7.35–7.31 (4H; m; C-*H*_aromatic_), 7.29–7.24 (1H; m; C-*H*_aromatic_), 7.20 (1H; t; *J* = 7.9 Hz; C-*H*_aromatic_), 6.85–6.76 (3H; m; C-*H*_aromatic_), 4.50 (2H; s; C*H*_2_), 3.94 (2H; t; *J* = 6.5 Hz; C*H*_2_), 3.67 (3H; s; C*H*_3_), 3.57 (2H; s; C*H*_2_), 3.49 (2H; t; *J* = 6.3 Hz; C*H*_2_), 1.82–1.75 (2H; m; C*H*_2_), 1.71–1.65 (2H; m; C*H*_2_), 1.59–1.51 (2H; m; C*H*_2_) ppm. ^13^C-NMR (125 MHz, [D_6_]DMSO, 25 °C, TMS): δ = 171.96, 159.28, 138.66, 135.37, 129.56, 128.41, 127.67, 127.55, 121.46, 115.54, 113.19, 72.97, 70.27, 67.77, 52.08, 41.27, 29.57, 29.15, 22.86 ppm. HRMS-TOF: *m*/*z* [*M* + H]^+^ calculated for C_21_H_26_O_4_: 343.1909, found: 343.1898.

In analogy to **8c** the following derivatives were synthesized:

*Methyl 2-(3-{[3-(benzyloxy)propyl]oxy}phenyl)acetate* (**8a**; ZHAWOC4557): The title compound **8a** was obtained as a white solid in 80% yield: ^1^H-NMR (500 MHz, [D_6_]DMSO, 25 °C, TMS): δ = 7.35–7.29 (4H; m; C-*H*_aromatic_), 7.29–7.24 (1H; m; C-*H*_aromatic_), 7.24–7.18 (1H; t; m; C-*H*_aromatic_), 6.86–6.76 (3H; m; C-*H*_aromatic_), 4.51 (2H; s; C*H*_2_), 4.06 (2H; t; *J* = 6.3 Hz; C*H*_2_), 3.67 (3H; s; C*H*_3_), 3.65 (2H; t; *J* = 6.3 Hz; C*H*_2_), 3.57 (2H; s; C*H*_2_), 2.07 (2H; quint; *J* = 6.19 Hz; C*H*_2_) ppm. ^13^C-NMR (125 MHz, [D_6_]DMSO, 25 °C, TMS): δ = 171.93, 159.20, 138.47, 135.39, 129.56, 128.41, 127.65, 127.59, 121.56, 115.63, 113.21, 73.07, 66.87, 64.82, 52.06, 41.25, 29.80 ppm. MS: *m*/*z* [*M* + Na]^+^ 337.

*Methyl 2-(3-{[4-(benzyloxy)butyl]oxy}phenyl)acetate* (**8b**; ZHAWOC4558): The title compound **8b** was obtained as a white solid in 85% yield: ^1^H-NMR (500 MHz, [D_6_]DMSO, 25 °C, TMS): δ = 7.35–7.30 (4H; m; C-*H*_aromatic_), 7.30–7.23 (1H; m; C-*H*_aromatic_), 7.22–7.16 (1H; t; m; C-*H*_aromatic_), 6.85–6.74 (3H; m; C-*H*_aromatic_), 4.50 (2H; s; C*H*_2_), 3.95 (2H; t; *J* = 6.14 Hz; C*H*_2_), 3.66 (3H; s; C*H*_3_), 3.56 (2H; s; C*H*_2_), 3.52 (2H; t; *J* = 6.14 Hz; C*H*_2_), 1.93–1.72 (4H; m; C*H*_2_) ppm. ^13^C-NMR (125 MHz, [D_6_]DMSO, 25 °C, TMS): δ = 171.93, 159.26, 138.63, 135.39, 129.56, 128.41, 127.67, 127.57, 121.49, 115.58, 113.20, 72.94, 69.98, 67.60, 52.04, 41.26, 26.42, 26.22 ppm. MS: *m*/*z* [*M* + Na]^+^ 351.

*Methyl 2-(3-{[6-(benzyloxy)hexyl]oxy}phenyl)acetate* (**8d**; ZHAWOC4928): The title compound **8d** was obtained as a white solid in 84% yield: ^1^H-NMR (500 MHz, [D_6_]DMSO, 25 °C, TMS): δ = 7.35–7.31 (4H; m; C-*H*_aromatic_), 7.29–7.25 (1H; m; C-*H*_aromatic_), 7.21 (1H; t; *J* = 7.88 Hz; C-*H*_aromatic_), 6.85–6.77 (3H; m; C-*H*_aromatic_), 4.50 (2H; s; C*H*_2_), 3.93 (2H; t; *J* = 6.5 Hz; C*H*_2_), 3.68 (3H; s; C*H*_3_), 3.58 (2H; s; C*H*_2_), 3.48 (2H; t; *J* = 6.5 Hz; C*H*_2_), 1.81–1.74 (2H; m; C*H*_2_), 1.68–1.62 (2H; m; C*H*_2_), 1.51–1.42 (4H; m; C*H*_2_) ppm. ^13^C-NMR (125 MHz, [D_6_]DMSO, 25 °C, TMS): δ = 171.97, 159.29, 138.69, 135.34, 129.54, 128.38, 127.66, 127.52, 121.43, 115.53, 113.19, 72.92, 70.35, 67.81, 52.07, 41.27, 29.74, 29.27, 26.03, 25.96 ppm. MS: *m*/*z* [*M* + Na]^+^ 379.

*2-(3-{[5-(benzyloxy)pentyl]oxy}phenyl)acetic acid* (**9c**; ZHAWOC4929): The ester (**8c**) (4.30 g, 12.57 mmol) was dissolved in methanol (220 mL) and stirred at ambient temperature. Potassium hydroxide 10% in water (220 mL) was added over 10 min. and the mixture was stirred for another 20 min. Methanol was removed in vacuum and the aqueous phase extracted with diethyl ether (200 mL). The aqueous phase was acidified with concentrated hydrochloric acid and extracted with diethyl ether (200 mL). The second organic phase was dried over sodium sulfate and concentrated in vacuum to obtain the title compound **9c** as a white solid (4.13 g, 100% yield) : ^1^H-NMR (500 MHz, [D_6_]DMSO, 25 °C, TMS): δ = 7.35–7.31 (4H; m; C-*H*_aromatic_), 7.30–7.25 (1H; m; C-*H*_aromatic_), 7.21 (1H; t; *J* = 7.9 Hz; C-*H*_aromatic_), 6.86–6.77 (3H; m; C-*H*_aromatic_), 4.51 (2H; s; C*H*_2_), 3.94 (2H; t; *J* = 6.6 Hz; C*H*_2_), 3.59 (2H; s; C*H*_2_), 3.50 (2H; t; *J* = 6.6 Hz; C*H*_2_), 1.82–1.75 (2H; m; C*H*_2_), 1.72–1.65 (2H; m; C*H*_2_), 1.58–1.50 (2H; m; C*H*_2_) ppm. ^13^C-NMR (125 MHz, [D_6_]DMSO, 25 °C, TMS): δ = 177.07, 159.27, 138.54, 134.73, 129.61, 128.40, 127.70, 127.57, 121.56, 115.66, 113.37, 72.94, 70.24, 67.77, 41.09, 29.50, 29.10, 22.80 ppm. HRMS-TOF: *m*/*z* [*M* + H]^+^ calculated for C_20_H_24_O_4_: 329.1753, found: 329.1748.

In analogy to **9c** the following derivatives were synthesized:

*2-(3-{[3-(benzyloxy)propyl]oxy}phenyl)acetic acid* (**9a**; ZHAWOC4559): The title compound **9a** was obtained as a white solid in 89% yield: ^1^H-NMR (500 MHz, [D_6_]DMSO, 25 °C, TMS): δ = 8.46 (1H; br. s.; COO*H*), 7.33–7.17 (6H; m; C-*H*_aromatic_), 6.87–6.77 (3H; m; C-*H*_aromatic_), 4.51 (2H; s; C*H*_2_), 4.05 (2H; t; *J* = 6.2 Hz; C*H*_2_), 3.64 (2H; t; *J* = 6.2 Hz; C*H*_2_), 3.57 (2H; s; C*H*_2_), 2.06 (2H; quint; *J* = 6.2 Hz ; C*H*_2_) ppm. ^13^C-NMR (125 MHz, [D_6_]DMSO, 25 °C, TMS): δ = 177.35, 159.20, 138.33, 134.79, 129.63, 128.43, 127.72, 127.65, 121.70, 115.82, 113.40, 73.06, 66.87, 64.84, 41.13, 29.73 ppm. MS: *m*/*z* [*M −* H]^−^ 299.

*2-(3-{[4-(benzyloxy)butyl]oxy}phenyl)acetic acid* (**9b**; ZHAWOC4560): The title compound **9b** was obtained as a white solid in quantitative yield: ^1^H-NMR (500 MHz, [D_6_]DMSO, 25 °C, TMS): δ = 7.35–7.31 (4H; m; C-*H*_aromatic_), 7.30–7.25 (1H; m; C-*H*_aromatic_), 7.21 (1H; t; *J* = 7.8 Hz; C-*H*_aromatic_), 6.86–6.77 (3H; m; C-*H*_aromatic_), 4.51 (2H; s; C*H*_2_), 3.96 (2H; t; *J* = 6.4 Hz; C*H*_2_), 3.59 (2H; s; C*H*_2_), 3.54 (2H; t; *J* = 6.4 Hz; C*H*_2_), 1.90–1.83 (2H; m; C*H*_2_), 1.82–1.75 (2H; m; C*H*_2_) ppm. ^13^C-NMR (125 MHz, [D_6_]DMSO, 25 °C, TMS): δ = 177.27, 159.24, 138.50, 134.69, 129.62, 128.41, 127.71, 127.59, 121.59, 115.68, 113.39, 72.92, 69.93, 67.59, 41.08, 26.35, 26.15 ppm. MS: *m*/*z* [*M* − H]^−^ 313.

*2-(3-{[6-(benzyloxy)hexyl]oxy}phenyl)acetic acid* (**9d**; ZHAWOC4930): The title compound **9d** was obtained as a white solid in 94% yield: ^1^H-NMR (500 MHz, [D_6_]DMSO, 25 °C, TMS): δ = 7.35–7.31 (4H; m; C-*H*_aromatic_), 7.29–7.24 (1H; m; C-*H*_aromatic_), 7.21 (1H; t; *J* = 7.8 Hz; C-*H*_aromatic_), 6.85–6.78 (3H; m; C-*H*_aromatic_), 4.50 (2H; s; C*H*_2_), 3.92 (2H; t; *J* = 6.6 Hz; C*H*_2_), 3.58 (2H; s; C*H*_2_), 3.47 (2H; t; *J* = 6.6 Hz; C*H*_2_), 1.80-1.73 (2H; m; C*H*_2_), 1.67–1.61 (2H; m; C*H*_2_), 1.50–1.38 (4H; m; C*H*_2_) ppm. ^13^C-NMR (125 MHz, [D_6_]DMSO, 25 °C, TMS): δ = 177.17, 159.30, 138.55, 134.80, 129.61, 128.41, 127.73, 127.59, 121.57, 115.68, 113.40, 72.89, 70.34, 67.84, 41.15, 29.67, 29.24, 26.00, 25.94 ppm. MS: *m*/*z* [*M* − H]^−^ 341.

*N-(2-benzyl-1,3-dioxo-2,3-dihydro-1H-isoindol-5-yl)-2-(3-{[5-(benzyloxy)pentyl]oxy}phenyl)acetamide* (**10c**; ZHAWOC5606): The acid (**9c**) (4.10 g, 12.49 mmol) was stirred in an excess of thionylchloride at 55 °C for 1 h. After removal of excess thionylchloride in vacuum the acid chloride was dissolved in tetrahydrofuran (10 mL) and added to a solution of 5-amino-2-benzylisoindoline-1,3-dione (**6**) (2.42 g, 9.60 mmol) in tetrahydrofuran (200 mL) under argon at ambient temperature. Diisopropylethylamine (1.90 g, 14.70 mmol) was added and the mixture was stirred at ambient temperature for 2 h. After removal of the tetrahydrofuran in vacuum, ethyl acetate (200 mL) and 10% citric acid (200 mL) were added and the resulting phases were separated. The organic phase was washed with 10% sodium bicarbonate (200 mL) and brine (200 mL), dried over sodium sulfate and concentrated in vacuum. Purification by chromatography on silica gel (Gradient: 0%–100% ethyl acetate in cyclohexane) afforded the title compound **10c** as a yellow solid (3.56 g, 66% yield): ^1^H-NMR (500 MHz, [D_6_]DMSO, 25 °C, TMS): δ = 7.89 (1H; d; *J* = 2.1 Hz; C-*H*_aromatic_), 7.84 (1H; dd; *J* = 8.2 Hz, 2.1 Hz; C-*H*_aromatic_), 7.74 (1H; d; *J* = 8.2 Hz; C-*H*_aromatic_), 7.71 (1H; br. s; N-*H*), 7.43–7.39 (2H; m; C-*H*_aromatic_), 7.37–7.24 (9H; m; C-*H*_aromatic_), 6.93–6.81 (3H; m; C-*H*_aromatic_), 4.82 (2H; s; C*H*_2_), 4.53 (2H; m; C*H*_2_), 3.98 (2H; t; *J* = 6.5 Hz; C*H*_2_), 3.74 (2H; s; C*H*_2_), 3.53 (2H; t; *J* = 6.5 Hz; C*H*_2_), 1.87–1.77 (2H; m; C*H*_2_), 1.76–1.67 (2H; m; C*H*_2_), 1.63––.53 (2H; m; C*H*_2_) ppm. ^13^C-NMR (125 MHz, [D_6_]DMSO, 25 °C, TMS): δ = 171.22, 169.33, 167.53, 159.83, 143.21, 136.35, 130.47, 128.66, 128.49, 128.38, 127.79, 127.65, 127.56, 124.45, 121.41, 115.83, 114.08, 113.76, 72.97, 70.23, 67.88, 44.88, 41.64, 29.52, 29.06, 22.84 ppm. HRMS-TOF: *m*/*z* [*M* + H]^+^ calculated for C_35_H_34_N_2_O_5_: 563.2546, found: 563.2542.

In analogy to **10c** the following derivatives were synthesized:

*N-(2-benzyl-1,3-dioxo-2,3-dihydro-1H-isoindol-5-yl)-2-(3-{[3-(benzyloxy)propyl]oxy}phenyl)acetamide* (**10a**; ZHAWOC4754): The title compound **10a** was obtained as a yellow solid in 74% yield: ^1^H-NMR (500 MHz, [D_6_]DMSO, 25 °C, TMS): δ = 8.08 (1H; s; N*H*), 7.90 (1H; d; *J* = 1.8 Hz; C-*H*_aromatic_), 7.82 (1H; dd; *J* = 8.2 Hz, 1.8 Hz; C-*H*_aromatic_), 7.68 (1H; d; *J* = 8.2 Hz; C-*H*_aromatic_), 7.39–7.19 (11H; m; C-*H*_aromatic_), 6.89–6.76 (3H; m; C-*H*_aromatic_), 4.78 (2H; s; C*H*_2_), 4.50 (2H; m; C*H*_2_), 4.05 (2H; t; *J* = 6.2 Hz; C*H*_2_), 3.67 (2H; s; C*H*_2_), 3.64 (2H; t; *J* = 6.2 Hz; C*H*_2_), 2.11–2.02 (2H; m; C*H*_2_) ppm. ^13^C-NMR (125 MHz, [D_6_]DMSO, 25 °C, TMS): δ = 171.27, 169.55, 167.62, 167.57, 159.62, 143.46, 138.33, 136.36, 135.22, 133.51, 130.29, 128.66, 128.43, 128.39, 127.79, 127.61, 126.78, 124.42, 123.85, 121.47, 115.82, 114.14, 113.64, 73.04, 66.78, 64.92, 44.72, 41.62, 29.68 ppm. MS: *m*/*z* [*M* − H]^−^ 533; [*M* + Na]^+^ 557.

*N-(2-benzyl-1,3-dioxo-2,3-dihydro-1H-isoindol-5-yl)-2-(3-{[4-(benzyloxy)butyl]oxy}phenyl)acetamide* (**10b**; ZHAWOC4755): The title compound **10b** was obtained as a yellow solid in 64% yield: ^1^H-NMR (500 MHz, [D_6_]DMSO, 25 °C, TMS): δ = 8.21 (1H; s; N*H*), 7.91 (1H; d; *J* = 1.9 Hz; C-*H*_aromatic_), 7.83 (1H; dd; *J* = 8.2 Hz, 1.9 Hz; C-*H*_aromatic_), 7.68 (1H; d; *J* = 8.2 Hz; C-*H*_aromatic_), 7.40–7.19 (11H; m; C-*H*_aromatic_), 6.87–6.77 (3H; m; C-*H*_aromatic_), 4.78 (2H; s; C*H*_2_), 4.50 (2H; m; C*H*_2_), 3.94 (2H; t; *J* = 6.0 Hz; C*H*_2_), 3.66 (2H; s; C*H*_2_), 3.53 (2H; t; *J* = 6.0 Hz; C*H*_2_), 1.92–1.71 (4H; m; C*H*_2_) ppm. ^13^C-NMR (125 MHz, [D_6_]DMSO, 25 °C, TMS): δ = 169.63, 167.63, 167.58, 159.64, 143.53, 138.48, 136.37, 135.26, 133.49, 130.22, 128.66, 128.42, 128.38, 127.78, 127.64, 127.57, 126.74, 124.40, 123.86, 121.38, 115.79, 114.15, 113.51, 72.92, 69.93, 47.66, 44.68, 41.61, 26.34, 26.15 ppm. MS: *m*/*z* [*M* − H]^−^ 547; [*M* + Na]^+^ 571.

*N-(2-benzyl-1,3-dioxo-2,3-dihydro-1H-isoindol-5-yl)-2-(3-{[4-(benzyloxy)butyl]oxy}phenyl)acetamide* (**10d**): The title compound **10d** was prepared from the acid **9d** and used directly for further synthesis without purification.

*N-(2-benzyl-1,3-dioxo-2,3-dihydro-1H-isoindol-5-yl)-2-{3-[(5-hydroxypentyl)oxy]phenyl}acetamide* (**2**; ZHAWOC5041): The benzyl ether (**10c**) (3.30 g, 5.87 mmol) was dissolved in ethanol (300 mL). Palladium 10% on activated charcoal (0.70 g, 0.59 mmol) was added and a hydrogen atmosphere was applied at 1 bar. After stirring at ambient temperature for 2 h the mixture was filtered over celite and concentrated in vacuum. Purification by chromatography on silica gel (Gradient: 0%–100% ethyl acetate in cyclohexane) afforded the title compound **2** as a white solid (2.30 g, 83% yield): ^1^H-NMR (500 MHz, [D_6_]DMSO, 25 °C, TMS): δ = 8.99 (1H; s; N-*H*), 7.98 (1H; d; *J* = 2 Hz; C-*H*_aromatic_), 7.83 (1H; dd; *J* = 8.2 Hz, 2 Hz; C-*H*_aromatic_), 7.64 (1H; d; *J* = 8.2 Hz; C-*H*_aromatic_), 7.37–7.32 (2H; m; C-*H*_aromatic_), 7.28–7.12 (4H; m; C-*H*_aromatic_), 6.84–6.69 (3H; m; C-*H*_aromatic_), 4.76 (2H; s; C*H*_2_), 3.85 (2H; t; *J* = 6.3 Hz; C*H*_2_), 3.64 (4H; br. s; 2 x C*H*_2_), 2.21 (1H; br. s; O-*H*), 1.77–1.69 (2H, m; C*H*_2_), 1.63–1.55 (2H; m; C*H*_2_), 1.53–1.44 (2H; m; C*H*_2_) ppm. ^13^C-NMR (125 MHz, [D_6_]DMSO, 25 °C, TMS): δ = 170.14, 167.68, 167.65, 159.38, 143.87, 136.33, 135.48, 133.34, 129.90, 128.61, 128.33, 127.74, 124.28, 123.93, 121.28, 112.72, 114.22, 113.08, 67.69, 62.48, 44.41, 41.54, 32.31, 28.94, 22.40 ppm. HRMS-TOF: *m*/*z* [*M* + H]^+^ calculated for C_28_H_28_N_2_O_5_: 473.2076, found: 473.2076.

*5-(3-{[(2-benzyl-1,3-dioxo-2,3-dihydro-1H-isoindol-5-yl)carbamoyl]methyl}phenoxy)pentanoic acid* (**3**; ZHAWOC5077): The alcohol (**2**) (2.20 g, 4.66 mmol), TEMPO (0.07 g, 0.352 mmol) and 0.67 M aqueous sodium phosphate (22 mL) in acetonitrile (26.4 mL) were stirred and heated to 40 °C. NaClO_2_ (1.12 g, 9.90 mmol in 5 mL water) and 10% NaOCl (2.2 mL) were added in parallel dropwise and it was stirred for 4 h. After cooling to ambient temperature water (20 mL) was added and the mixture was poured in an ice could solution of Na_2_SO_3_ (1.8 g in 30 mL water). The aqueous phase was extracted with diethylether (80 mL), acidified with 10% citric acid and again extracted with diethylether (2 × 100 mL). The organic phases were dried over sodium sulfate and concentrated in vacuum to obtain the title compound **3** as a white solid (1.20 g, 53% yield): ^1^H-NMR (500 MHz, [D_6_]DMSO, 25 °C, TMS): δ = 12.05 (1H; br. s; COO*H*), 10.77 (1H; s; N-*H*), 8.22 (1H; d; *J* = 1.6 Hz; C-*H*_aromatic_), 7.90 (1H; dd; *J* = 8.2 Hz, 1.6 Hz; C-*H*_aromatic_), 7.84 (1H; d; *J* = 8.2 Hz; C-*H*_aromatic_), 7.35–7.21 (6H; m; C-*H*_aromatic_), 6.93–6.80 (3H; m; C-*H*_aromatic_), 4.74 (2H; s; C*H*_2_), 3.96 (2H; t; *J* = 6.2 Hz; C*H*_2_), 3.69 (2H; s; C*H*_2_), 2.28 (2H; t; *J* = 7.5 Hz; C*H*_2_), 1.76–1.69 (2H; m; C*H*_2_), 1.68–1.60 (2H; m; C*H*_2_) ppm. ^13^C-NMR (125 MHz, [D_6_]DMSO, 25 °C, TMS): δ = 174.82, 170.44, 167.93, 167.76, 159.08, 145.23, 137.21, 137.14, 129.85, 129.04, 127.85, 127.82, 125.78, 124.93, 123.86, 121.75, 116.00, 113.36, 113.04, 67.48, 43.86, 41.31, 33.79, 28.61, 21.70 ppm. HRMS-TOF: *m*/*z* [*M* + H]^+^ calculated for C_28_H_26_N_2_O_6_: 487.1869, found: 487.1850.

*5-(3-{[(2-benzyl-1,3-dioxo-2,3-dihydro-1H-isoindol-5-yl)carbamoyl]methyl}phenoxy)-N-methanesulfonylpentanamide* (**4**; ZHAWOC5079): The acid (**3**) (106 mg, 0.218 mmol) was dissolved in dichloromethane (5 mL) and cooled to 0 °C. EDC·HCl (63 mg, 0.327 mmol), methansulfonamide (23 mg, 0.240 mmol) and DMAP (40 mg, 0.327 mmol) were added and stirred at 0 °C for further 20 min. After warming up to ambient temperature it was stirred for another 12 h. The reaction was quenched by adding 10% citric acid (5 mL). The precipitate was filtered off and dissolved in DMSO (1 mL). Purification by reversed phase chromatography on silica gel C18 (Gradient: 0%–100% methanol in water) afforded the title compound **4** as a white solid (50 mg, 41% yield): ^1^H-NMR (500 MHz, [D_6_]DMSO, 25 °C, TMS): δ = 11.68 (1H; s; N-*H*), 10.76 (1H; s; N-*H*), 8.22 (1H; d: *J* = 1.9 Hz; C-*H*_aromatic_), 7.90 (1H; dd; *J* = 8.5 Hz, 1.9 Hz; C-*H*_aromatic_), 7.85 (1H; d; *J* = 8.5 Hz; C-*H*_aromatic_), 7.35–7.21 (6H; m; C-*H*_aromatic_), 6.94–6.81 (3H; m; C-*H*_aromatic_), 4.74 (2H; s; C*H*_2_), 3.96 (2H; t; J = 5.7 Hz; C*H*_2_), 3.69 (2H; s; C*H*_2_), 3.22 (3H; s ; C*H*_3_), 2.35 (2H; t; J = 7.2 Hz; C*H*_2_), 1.76–1.60 (4H; m; 2 × C*H*_2_) ppm. ^13^C-NMR (125 MHz, [D_6_]DMSO, 25 °C, TMS): δ = 172.94, 170.43, 167.93, 167.76, 159.05, 145.22, 137.21, 137.15, 133.55, 129.85, 129.04, 127.86, 127.83, 125.79, 124.94, 123.85, 121.80, 115.99, 113.35, 113.06, 67.37, 43.84, 41.45, 41.32, 35.47, 28.42, 21.29 ppm. HRMS-TOF: *m*/*z* [*M* + H]^+^ calculated for C_29_H_29_N_3_O_7_S: 564.1804, found: 564.1808.

*5-(3-{[(2-benzyl-1,3-dioxo-2,3-dihydro-1H-isoindol-5-yl)carbamoyl]methyl}phenoxy)-N-methylpentanamide* (**5**; ZHAWOC5080): The acid (**3**) (100 mg, 0.206 mmol) was dissolved in tetrahydrofuran (3 mL) and diisopropylethylamine (138 mg, 1.070 mmol), HATU (215 mg, 0.565 mmol) such as methylamine 2 M in THF (0.206 mL, 0.412 mmol) were added. The mixture was stirred at ambient temperature for 18 h before it was concentrated in vacuum and purified by chromatography on silica gel (Gradient: 0%–100% ethyl acetate in Cyclohexane) to obtain the title compound **5** as a white solid (40 mg, 39% yield): ^1^H-NMR (500 MHz, [D_6_]DMSO, 25 °C, TMS): δ = 10.76 (1H; s; N-*H*), 8.21 (1H; d; *J* = 1.9 Hz; C-*H*_aromatic_), 7.90 (1H; dd; *J* = 8.2 Hz, 1.9 Hz; C-*H*_aromatic_), 7.84 (1H; d; *J* = 8.2 Hz; C-*H*_aromatic_), 7.71 (1H; br. q; *J* = 4.5 Hz; N-*H*), 7.35–7.20 (6H; m; C-*H*_aromatic_), 6.92–6.79 (3H; m; C-*H*_aromatic_), 4.74 (2H; s; C*H*_2_), 3.94 (2H; t; *J* = 6 Hz; C*H*_2_), 3.68 (2H; s; C*H*_2_), 2.55 (3H; d; *J* = 4.5 Hz; C*H*_3_), 2.11 (2H; t; *J* = 7.3 Hz; C*H*_2_), 1.72–1.58 (4H; m; 2 × C*H*_2_) ppm. ^13^C-NMR (125 MHz, [D_6_]DMSO, 25 °C, TMS): δ = 172.71, 170.44, 167.93 167.76, 159.09, 145.23, 137.21, 137.14, 129.84, 129.04, 127.86, 127.83, 125.79, 124.93, 123.85, 121.74, 115.98, 113.36, 113.05, 67.46, 43.85, 41.32, 35.38, 28.78, 25.87, 22.36 ppm. HRMS-TOF: *m*/*z* [*M* + H]^+^ calculated for C_29_H_29_N_3_O_5_: 500.2185, found: 500.2181.

The following alcohols were synthesized according to compound (**2**):

*N-(2-benzyl-1,3-dioxo-2,3-dihydro-1H-isoindol-5-yl)-2-[3-(3-hydroxypropoxy)phenyl]acetamide* (**11**; ZHAWOC4756): The title compound was isolated as a white solid in 57% yield: ^1^H-NMR (500 MHz, [D_6_]DMSO, 25 °C, TMS): δ = 8.95 (1H; s; N-*H*), 7.95 (1H; d; *J* = 1.7 Hz; C-*H*_aromatic_), 7.77 (1H; dd; *J* = 8.2 Hz, 1.7 Hz; C-*H*_aromatic_), 7.60 (1H; d; *J* = 8.2 Hz; C-*H*_aromatic_), 7.38–7.05 (6H; m; C-*H*_aromatic_), 6.82–6.64 (3H; m; C-*H*_aromatic_), 4.74 (2H; s; C*H*_2_), 3.97 (2H; t; *J* = 6 Hz; C*H*_2_), 3.77 (2H; t; *J* = 5.9 Hz; C*H*_2_), 3.58 (2H; s; C*H*_2_), 2.95 (1H; br. s; O-*H*), 1.94 (2H; quint.; *J* = 5.9 Hz; C*H*_2_) ppm. ^13^C-NMR (125 MHz, [D_6_]DMSO, 25 °C, TMS): δ = 170.31, 167.82, 167.73, 159.19, 143.74, 136.27, 135.46, 133.32, 130.02, 128.69, 128.34, 127.84, 126.55, 124.36, 124.08, 121.57, 115.70, 114.32, 113.20, 65.09, 59.68, 44.37, 41.62, 31.97 ppm. HRMS-TOF: *m*/*z* [*M* + H]^+^ calculated for C_26_H_24_N_2_O_5_: 445.1763, found: 445.1776. 

*N-(2-benzyl-1,3-dioxo-2,3-dihydro-1H-isoindol-5-yl)-2-[3-(4-hydroxybutoxy)phenyl]acetamide* (**12**; ZHAWOC4757): The title compound was isolated as a white solid in 48% yield: ^1^H-NMR (500 MHz, [D_6_]DMSO, 25 °C, TMS): δ = 8.93 (1H; s; N-*H*), 7.96 (1H; d; *J* = 1.75 Hz; C-*H*_aromatic_), 7.80 (1H; dd; *J* = 8.2 Hz, 1.75 Hz; C-*H*_aromatic_), 7.62 (1H; d; *J* = 8.2 Hz; C-*H*_aromatic_), 7.38–7.10 (6H; m; C-*H*_aromatic_), 6.85–6.66 (3H; m; C-*H*_aromatic_), 4.74 (2H; s; C*H*_2_), 3.87 (2H; t; *J* = 6 Hz; C*H*_2_), 3.66 (2H; t; *J* = 6.3 Hz; C*H*_2_), 3.61 (2H; s; C*H*_2_), 2.84 (1H; br. s; O-*H*), 1.85–1.73 (2H; m; C*H*_2_), 1.73–1.60 (2H; m; C*H*_2_) ppm. ^13^C-NMR (125 MHz, [D_6_]DMSO, 25 °C, TMS): δ = 170.26, 167.82, 167.72, 159.27, 143.78, 136.28, 135.47, 133.35, 130.04, 128.68, 128.34, 127.83, 126.55, 124.38, 124.06, 121.50, 115.83, 114.32, 113.18, 67.70, 62.35, 44.42, 41.61, 29.30, 25.76 ppm. HRMS-TOF: *m*/*z* [*M* + H]^+^ calculated for C_27_H_26_N_2_O_5_: 459.1920, found: 459.1922.

*N-(2-benzyl-1,3-dioxo-2,3-dihydro-1H-isoindol-5-yl)-2-{3-[(6-hydroxyhexyl)oxy]phenyl}acetamide* (**13**; ZHAWOC5042): The title compound was isolated as a white solid in 77% yield: ^1^H-NMR (500 MHz, [D_6_]DMSO, 25 °C, TMS): δ = 8.74 (1H; s; N-*H*), 7.94 (1H; d; *J* = 1.8 Hz; C-*H*_aromatic_), 7.82 (1H; dd; *J* = 8.2 Hz, 1.8 Hz; C-*H*_aromatic_), 7.65 (1H; d; *J* = 8.2 Hz; C-*H*_aromatic_), 7.38–7.32 (2H; m; C-*H*_aromatic_), 7.29–7.15 (4H; m; C-*H*_aromatic_), 6.85–6.72 (3H; m; C-*H*_aromatic_), 4.76 (2H; s; C*H*_2_), 3.86 (2H; t; *J* = 6.4 Hz; C*H*_2_), 3.64 (2H; s; C*H*_2_), 3.63 (2H; t; *J* = 6.5 Hz; C*H*_2_), 2.41 (1H; br. s; O-*H*), 1.76–1.67 (2H; m; ; C*H*_2_), 1.60–1.53 (2H; m; ; C*H*_2_), 1.47–1.33 (4H; m; ; C*H*_2_) ppm. ^13^C-NMR (125 MHz, [D_6_]DMSO, 25 °C, TMS): δ = 170.08, 167.74, 167.67, 159.51, 143.74, 136.30, 135.38, 133.38, 130.06, 128.67, 128.36, 127.80, 126.58, 124.38, 121.33, 115.76, 114.25, 113.30, 67.78, 62.71, 44.53, 41.61, 32.58, 29.13, 25.82, 25.53 ppm. HRMS-TOF: *m*/*z* [*M* + H]^+^ calculated for C_29_H_30_N_2_O_5_: 487.2233, found: 487.2211.

The following acids were synthesized according to compound (**3**):

*3-(3-{[(2-benzyl-1,3-dioxo-2,3-dihydro-1H-isoindol-5-yl)carbamoyl]methyl}phenoxy)propanoic acid* (**14**; ZHAWOC4767): The title compound was isolated as a white solid in 51% yield: ^1^H-NMR (500 MHz, [D_6_]DMSO, 25 °C, TMS): δ = 12.35 (1H; br. s; COO*H*), 10.76 (1H; s; N-*H*), 8.21 (1H; d; *J* = 1.7 Hz; C-*H*_aromatic_), 7.90 (1H; dd; *J* = 8.2 Hz, 1.7 Hz; C-*H*_aromatic_), 7.84 (1H; d; *J* = 8.2 Hz; C-*H*_aromatic_), 7.36–7.19 (6H; m; C-*H*_aromatic_), 6.96–6.78 (3H; m; C-*H*_aromatic_), 4.74 (2H; s; C*H*_2_), 4.15 (2H; t; *J* = 6 Hz; C*H*_2_), 3.69 (2H; s; C*H*_2_), 2.69 (2H; t; *J* = 6 Hz; C*H*_2_) ppm. ^13^C-NMR (125 MHz, [D_6_]DMSO, 25 °C, TMS): δ = 172.71, 170.42, 167.93, 167.76, 158.80, 145.23, 137.21, 133.55, 129.89, 129.04, 127.83, 125.79, 124.93, 123.86, 122.00, 115.91, 113.37, 113.10, 63.95, 43.83, 41.31, 34.56 ppm. HRMS-TOF: *m*/*z* [*M* + H]^+^ calculated for C_26_H_22_N_2_O_6_: 459.1556, found: 459.1557.

*4-(3-{[(2-benzyl-1,3-dioxo-2,3-dihydro-1H-isoindol-5-yl)carbamoyl]methyl}phenoxy)butanoic acid* (**15**; ZHAWOC4768): The title compound was isolated as a white solid in 84% yield: ^1^H-NMR (500 MHz, [D_6_]DMSO, 25 °C, TMS): δ = 12.12 (1H; br. s; COO*H*), 10.76 (1H; s; N-*H*), 8.21 (1H; d; *J* = 1.7 Hz; C-*H*_aromatic_), 7.90 (1H; dd; *J* = 8.2 Hz, 1.7 Hz; C-*H*_aromatic_), 7.84 (1H; d; *J* = 8.2 Hz; C-*H*_aromatic_), 7.37–7.18 (6H; m; C-*H*_aromatic_), 6.95–6.78 (3H; m; C-*H*_aromatic_), 4.74 (2H; s; C*H*_2_), 3.97 (2H; t; *J* = 6.4 Hz; C*H*_2_), 3.68 (2H; s; C*H*_2_), 2.38 (2H; t; *J* = 7.4 Hz; C*H*_2_), 1.93 (2H; quint.; *J* = 6.7 Hz; C*H*_2_) ppm. ^13^C-NMR (125 MHz, [D_6_]DMSO, 25 °C, TMS): δ = 174.62, 170.44, 167.93, 167.76, 158.97, 145.24, 137.21, 137.17, 129.86, 129.04, 127.85, 127.82, 124.92, 123.86, 121.83, 115.95, 113.37, 113.11, 66.97, 43.84, 41.31, 30.72, 24.80 ppm. HRMS-TOF: *m*/*z* [*M* + H]^+^ calculated for C_27_H_24_N_2_O_6_: 473.1712, found: 473.1709.

*6-(3-{[(2-benzyl-1,3-dioxo-2,3-dihydro-1H-isoindol-5-yl)carbamoyl]methyl}phenoxy)hexanoic acid* (**16**; ZHAWOC5078): The title compound was isolated as a white solid in 87% yield: ^1^H-NMR (500 MHz, [D_6_]DMSO, 25 °C, TMS): δ = 11.82 (1H; br. s; COO*H*), 10.78 (1H; s; N-*H*), 8.21 (1H; d; *J* = 1.8 Hz; C-*H*_aromatic_), 7.90 (1H; dd; *J* = 8.2 Hz, 1.8 Hz; C-*H*_aromatic_), 7.83 (1H; d; *J* = 8.2 Hz; C-*H*_aromatic_), 7.35–7.19 (6H; m; C-*H*_aromatic_), 6.93–6.78 (3H; m; C-*H*_aromatic_), 4.73 (2H; s; C*H*_2_), 3.93 (2H; t; *J* = 6.3 Hz; C*H*_2_), 3.68 (2H; s; C*H*_2_), 2.22 (2H; t; *J* = 7.3 Hz; C*H*_2_), 1.73–1.66 (2H; m; C*H*_2_), 1.59–1.51 (2H; m; C*H*_2_), 1.45–1.37 (2H; m; C*H*_2_) ppm. ^13^C-NMR (125 MHz, [D_6_]DMSO, 25 °C, TMS): δ = 174.94, 170.45, 167.92, 167.75, 159.11, 145.24, 137.20, 137.14, 129.84, 129.04, 127.85, 127.82, 125.77, 124.91, 123.85, 121.72, 115.96, 113.36, 113.05, 67.67, 43.86, 41.31, 34.19, 28.93, 25.65, 24.79 ppm. HRMS-TOF: *m*/*z* [*M* + H]^+^ calculated for C_29_H_28_N_2_O_6_: 501.2025, found: 501.2022.

The following acyl sulfonamides were synthesized according to compound (**4**):

*3-(3-{[(2-benzyl-1,3-dioxo-2,3-dihydro-1H-isoindol-5-yl)carbamoyl]methyl}phenoxy)-N-methanesulfonylpropanamide* (**17**; ZHAWOC5136): The title compound was isolated as a white solid in 19% yield: ^1^H-NMR (500 MHz, [D_6_]DMSO, 25 °C, TMS): δ = 11.86 (1H; s; N-*H*), 10.77 (1H; s; N-*H*), 8.21 (1H; d: *J* = 1.9 Hz; C-*H*_aromatic_), 7.89 (1H; dd; *J* = 8.2 Hz, 1.9 Hz; C-*H*_aromatic_), 7.84 (1H; d; *J* = 8.2 Hz; C-*H*_aromatic_), 7.35–7.21 (6H; m; C-*H*_aromatic_), 6.94–6.81 (3H; m; C-*H*_aromatic_), 4.74 (2H; s; C*H*_2_), 4.18 (2H; t; *J* = 6 Hz; C*H*_2_), 3.69 (2H; s; C*H*_2_), 3.22 (3H; s ; C*H*_3_), 2.75 (2H; t; *J* = 6 Hz; C*H*_2_) ppm. ^13^C-NMR (125 MHz, [D_6_]DMSO, 25 °C, TMS): δ = 170.90, 170.41, 167.93, 167.76, 158.72, 145.22, 137.21, 133.55, 129.90, 129.04, 127.86, 127.82, 125.80, 124.94, 123.86, 122.10, 115.99, 113.16, 63.33, 43.82, 41.46, 41.32, 36.15 ppm. HRMS-TOF: *m*/*z* [*M* + H]^+^ calculated for C_27_H_25_N_3_O_7_S: 536.1491, found: 536.1483.

*4-(3-{[(2-benzyl-1,3-dioxo-2,3-dihydro-1H-isoindol-5-yl)carbamoyl]methyl}phenoxy)-N-methanesulfonylbutanamide* (**18**; ZHAWOC5135): The title compound was isolated as a white solid in 26% yield: ^1^H-NMR (500 MHz, [D_6_]DMSO, 25 °C, TMS): δ = 11.71 (1H; s; N-*H*), 10.77 (1H; s; N-*H*), 8.21 (1H; d: *J* = 1.8 Hz; C-*H*_aromatic_), 7.89 (1H; dd; *J* = 8.2 Hz, 1.8 Hz; C-*H*_aromatic_), 7.84 (1H; d; *J* = 8.2 Hz; C-*H*_aromatic_), 7.35–7.21 (6H; m; C-*H*_aromatic_), 6.93–6.80 (3H; m; C-*H*_aromatic_), 4.74 (2H; s; C*H*_2_), 3.96 (2H; t; *J* = 6.2 Hz; C*H*_2_), 3.68 (2H; s; C*H*_2_), 3.21 (3H; s ; C*H*_3_), 2.44 (2H; t; *J* = 7.4 Hz; C*H*_2_), 1.95 (2H; quint.; *J* = 6.7 Hz; C*H*_2_) ppm. ^13^C-NMR (125 MHz, [D_6_]DMSO, 25 °C, TMS): δ = 172.77, 170.42, 167.93, 167.76, 158.92, 145.22, 137.21, 137.18, 133.55, 129.87, 129.04, 127.86, 127.83, 125.80, 124.94, 123.86, 121.90, 115.93, 113.36, 113.13, 66.89, 43.83, 41.46, 41.32, 32.55, 24.15 ppm. HRMS-TOF: *m*/*z* [*M* + H]^+^ calculated for C_28_H_27_N_3_O_7_S: 550.1648, found: 550.1640.

### 4.3. In Silico Studies

Molecular modeling experiments were performed using the Molecular Operating Environment MOE 2013.08 from Chemical Computing Group (www.chemcomp.com). Co-crystal structures of MMP-13 are available from the Protein Data Bank (www.rcsb.org). For the actual work PDB code 2OW9 was selected for the computational studies. In MOE the duplicates were eliminated using the sequence editor and the pocket was prepared for the docking studies via the Protonate 3D method applying the default values for temperature 300 K, pH 7 and salt 0.1. The ligands to be docked to the protein were imported from structural data files (SD files) to receive a MOE compatible molecular database. As the SD files did not contain 3D coordinates, they were generated directly using MOE rebuild3D with a root mean square deviation (RMSD) gradient of 0.1. For docking experiments the Amber10:EHT force field [32,33] was used. The pharmacophore placement was applied with the pharmacophore set of three hydrogen bond acceptors (Acc1) used in the query. The docked poses were subsequently analyzed with respect to their scores and interactions with the catalytic zinc ion.

### 4.4. In Vitro Assays 

IC_50_ values were determined at Reaction Biology Corporation, Malvern, PA, USA in triplicates using 10 concentrations starting at 10 μM with 3 fold dilution. The substrate used for the determinations was the (5-FAM/QXLTM) FRET peptide. The buffer consisted of 50 mM HEPES at pH 7.5 with 10 mM CaCl_2_ and 0.01% Brij-35. 0.1 mg/mL BSA was added before use. As a control inhibitor GM6001 was used.

## 5. Conclusions

In conclusion, we could employ molecular recognition for evolving an allosteric inhibitor through the addition of properly arranged weak zinc binding fragments to obtain an inhibitor with dual binding mode. In addition, the lipophilic ligand efficiency could be improved to lead-like level.

For the definite confirmation of the binding mode, a co-crystal structure of the complex is required. This work is currently ongoing in our lab.

## Figures and Tables

**Table 1:** MMP-13 inhibitory data for compounds **1**–**5** and **11**–**18**.

Structure	Comp.	% Activity ^1^	IC_50_ [μM]	pIC_50_	clogP	LE ^2^	LLE ^3^
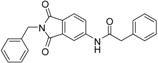	**1**	25 ± 4	9.8 ± 3.4	5.01	3.94	0.25	1.07
	ZHAWOC						
	928						
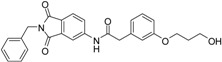	**11**	7.9 ± 0.4	0.586 ± 0.13	6.23	3.68	0.26	2.55
	ZHAWOC						
	4756						
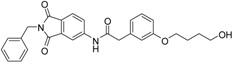	**12**	7.6 ± 0.5	0.454 ± 0.20	6.34	4.12	0.26	2.22
	ZHAWOC						
	4757						
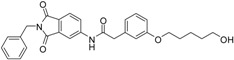	**2**	9.1 ± 0.9	0.822 ± 0.21	6.09	4.56	0.24	1.53
	ZHAWOC						
	5041						
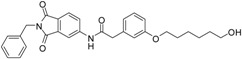	**13**	33.0 ± 2.9	n.d.^4^	n.d.^4^	5.00	n.d.^4^	n.d.^4^
	ZHAWOC						
	5042						
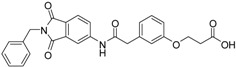	**14**	15.4 ± 0.3	n.d.^4^	n.d.^4^	3.52	n.d.^4^	n.d.^4^
	ZHAWOC						
	4767						
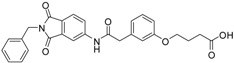	**15**	4.6 ± 0.6	0.777 ± 0.05	6.11	3.95	0.24	2.16
	ZHAWOC						
	4768						
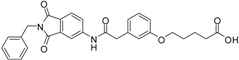	**3**	2.4 ± 0.1	0.134 ± 0.04	6.87	4.41	0.27	2.46
	ZHAWOC						
	5077						
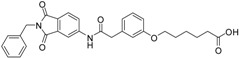	**16**	2.6 ± 0.3	0.280 ± 0.04	6.55	4.85	0.25	1.70
	ZHAWOC						
	5078						
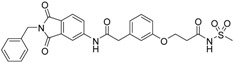	**17**	30.5 ± 0.8	n.d.^4^	n.d.^4^	2.70	n.d.^4^	n.d.^4^
	ZHAWOC						
	5136						
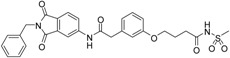	**18**	11.2 ± 0.3	0.896 ± 0.06	6.05	3.15	0.22	2.91
	ZHAWOC						
	5135						
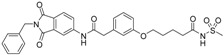	**4**	13.8 ± 1.4	0.767 ± 0.08	6.12	3.59	0.21	2.53
	ZHAWOC						
	5079						
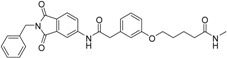	**5**	6.7 ± 0.5	0.328 ± 0.05	6.48	4.03	0.25	2.45
	ZHAWOC						
	5080						

^1^ Remaining enzyme activity at 10 μM inhibitor concentration; ^2^ Ligand Efficiency (LE) was calculated as 1.4(−*log*IC_50_)/N; N equals the number of non-hydrogen atoms; ^3^ LLE = pIC_50_ - clogP; metric for the estimate of drug likeness; ^4^ Not determined (n.d.) due to more than 15% remaining enzyme activity in single dose inhibitory assays at 10 μM inhibitor concentration.

## Supplementary Materials

Supplementary materials can be found at http://www.mdpi.com/1422-0067/17/3/314/s1.
