# Supplementary material for: Molecular Recognition of the Catalytic Zinc(II) Ion in MMP-13: Structure-Based Evolution of an Allosteric Inhibitor to Dual Binding Mode Inhibitors with Improved Lipophilic Ligand Efficiencies
Source: Int J Mol Sci. 2016 Mar 1;17(3):314. doi: 10.3390/ijms17030314 (PMC4813177; doi:10.3390/ijms17030314)

Supplementary Materials: Molecular Recognition of the Catalytic Zinc(II) Ion in MMP-13: Structure-Based Evolution of an Allosteric Inhibitor to Dual Binding Mode Inhibitors with Improved Lipophilic Ligand Efficiencies

Thomas Fischer and Rainer Riedl

N-(2-benzyl-1,3-dioxo-2,3-dihydro-1H-isoindol-5-yl)-2-{3-[(5-hydroxypentyl)oxy]phenyl}acetamide (**2**; ZHAW5041)

NMR

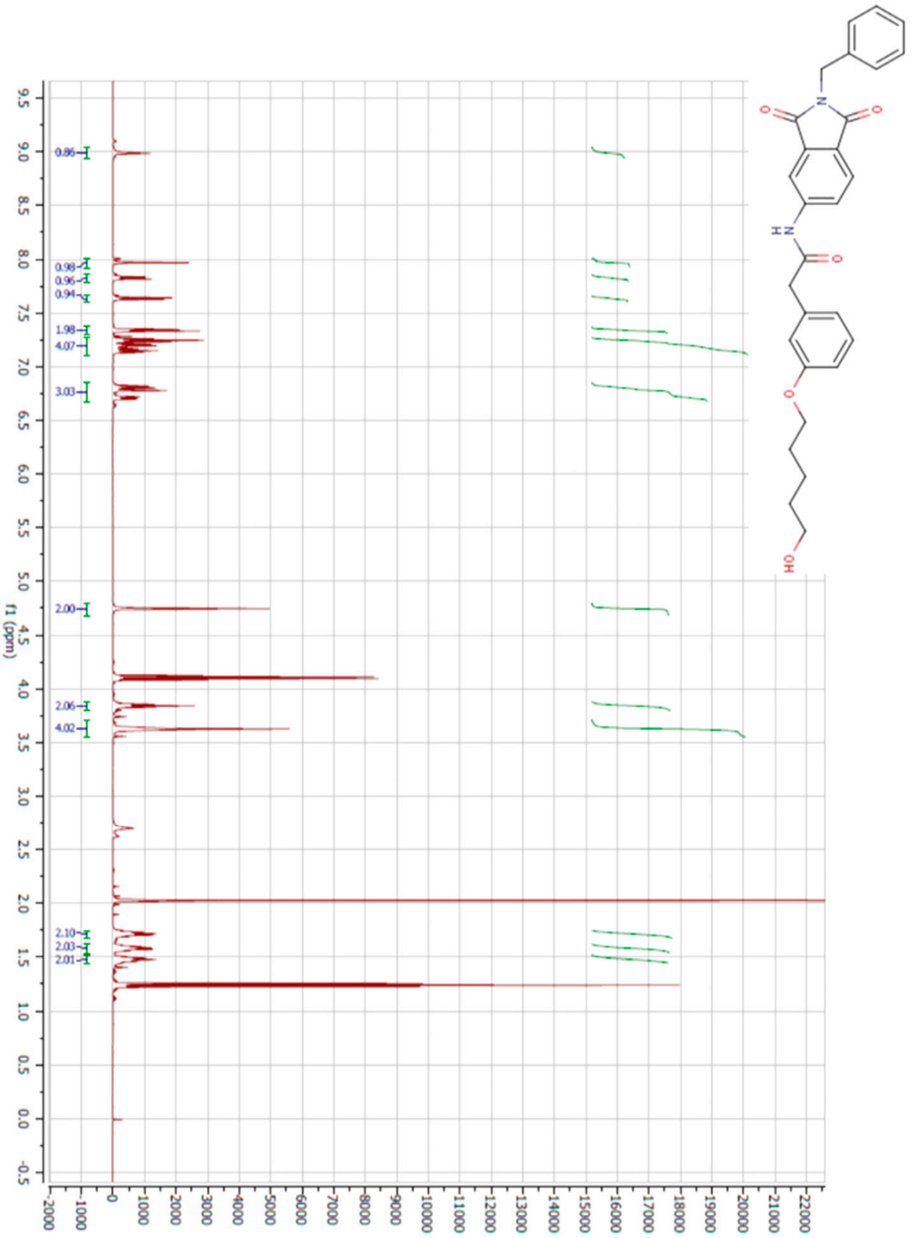

N-(2-benzyl-1,3-dioxo-2,3-dihydro-1H-isoindol-5-yl)-2-{3-[(5-hydroxypentyl)oxy]phenyl}acetamide (**2**; ZHAW5041)

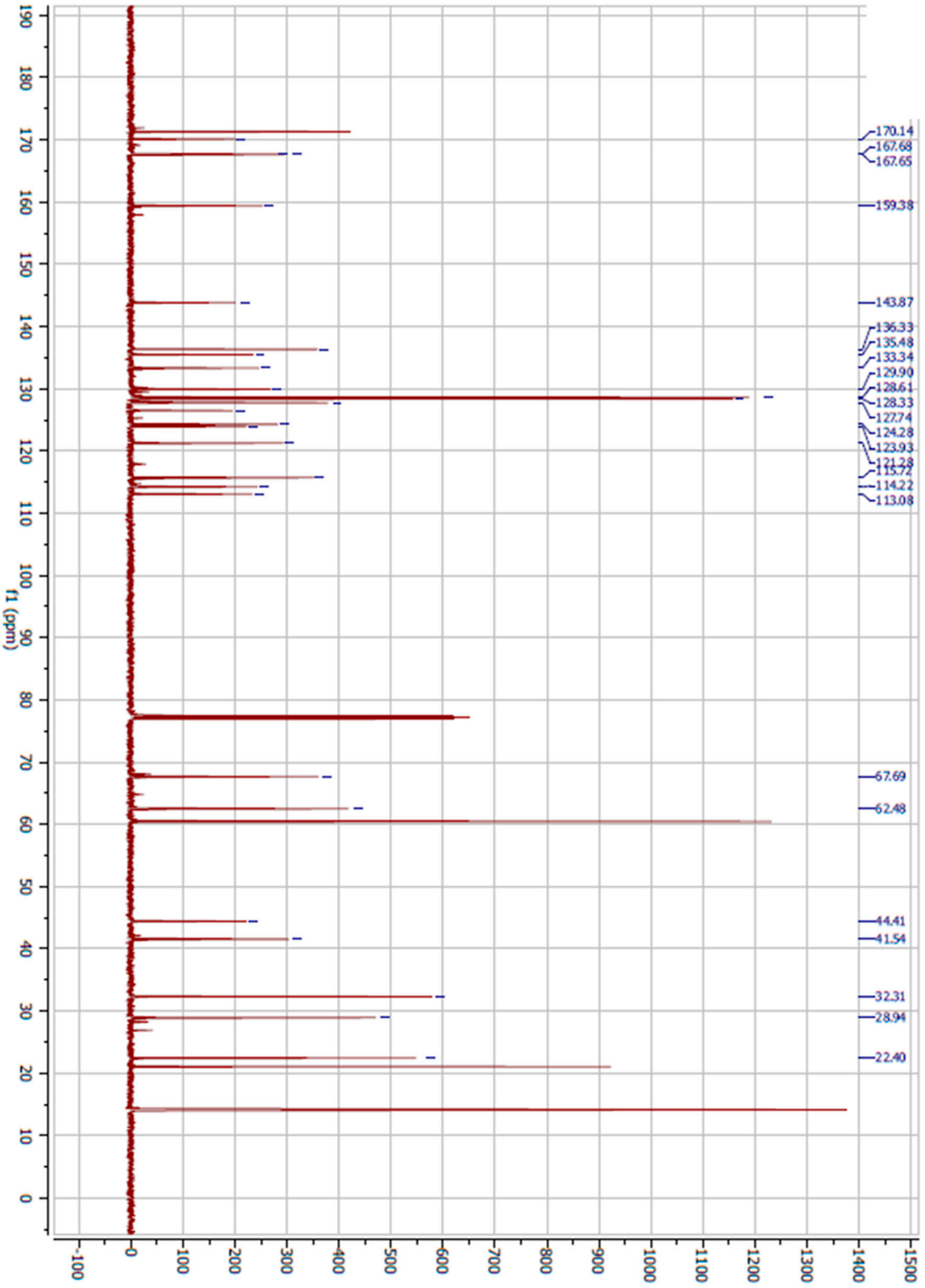

HRMS

N-(2-benzyl-1,3-dioxo-2,3-dihydro-1H-isoindol-5-yl)-2-{3-[(5-hydroxypentyl)oxy]phenyl}acetamide (**2**; ZHAW5041)

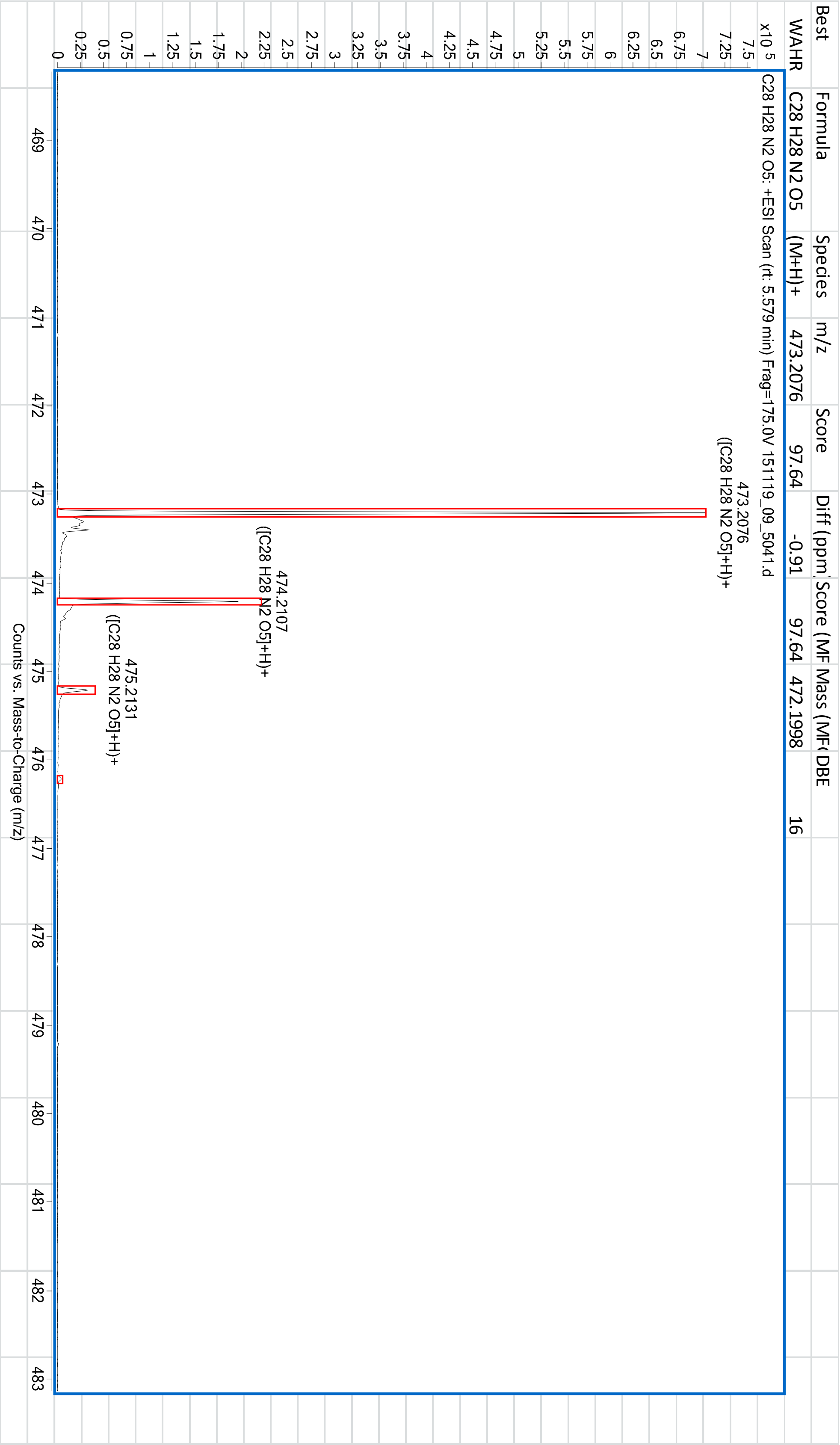

IC50

N-(2-benzyl-1,3-dioxo-2,3-dihydro-1H-isoindol-5-yl)-2-{3-[(5-hydroxypentyl)oxy]phenyl}acetamide (**2**; ZHAW5041)

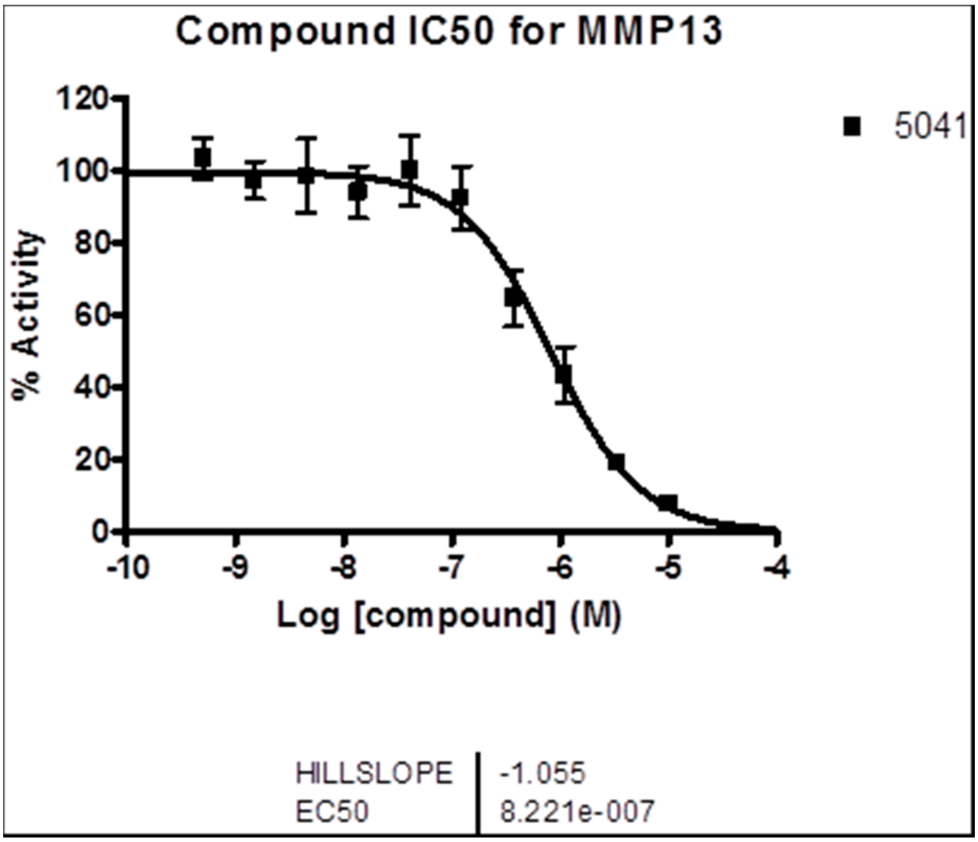

5-(3-{[(2-benzyl-1,3-dioxo-2,3-dihydro-1H-isoindol-5-yl)carbamoyl]methyl}phenoxy)pentanoic acid (**3**; ZHAW5077)

NMR

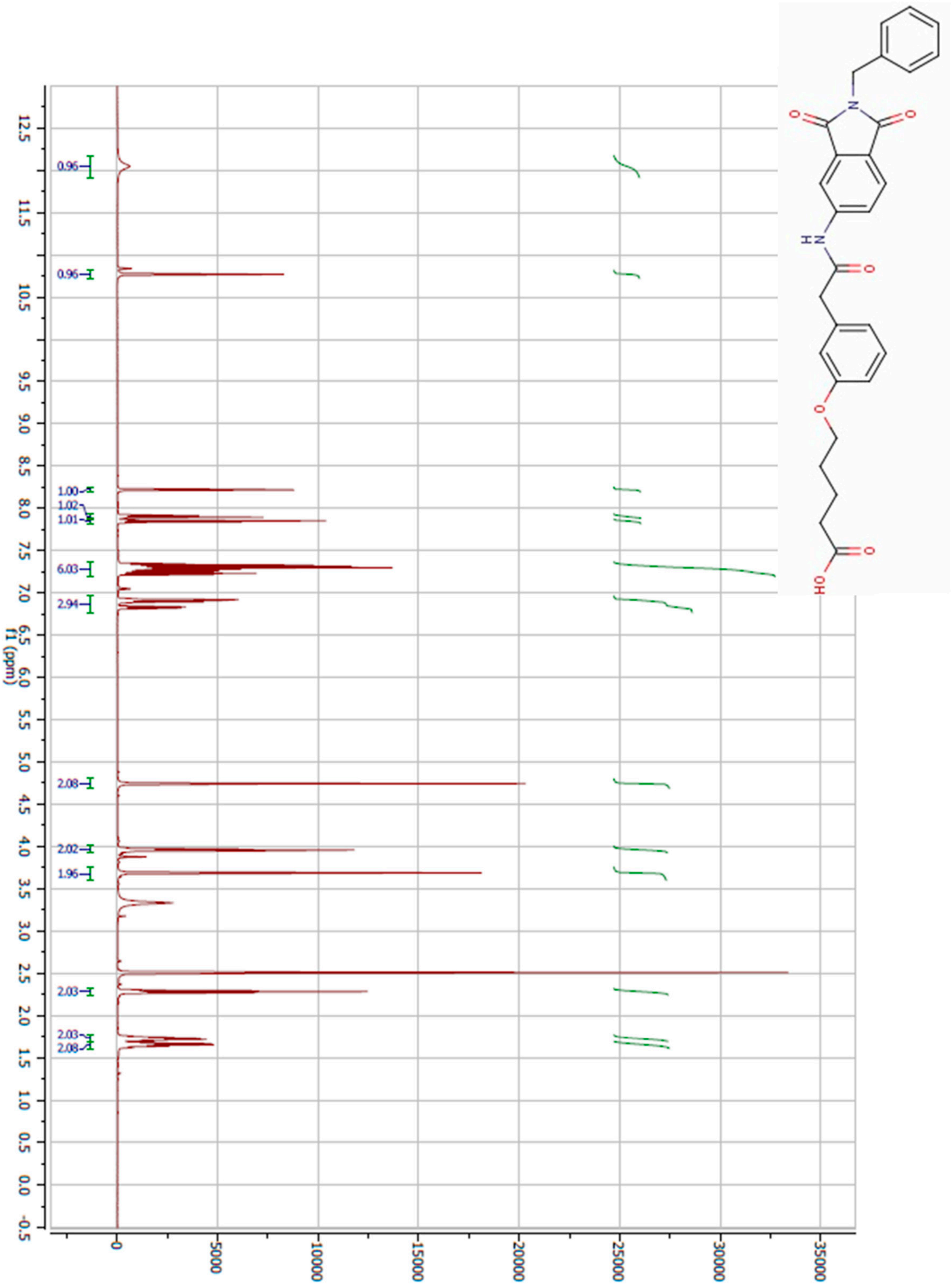

5-(3-{[(2-benzyl-1,3-dioxo-2,3-dihydro-1H-isoindol-5-yl)carbamoyl]methyl}phenoxy)pentanoic acid (**3**; ZHAW5077)

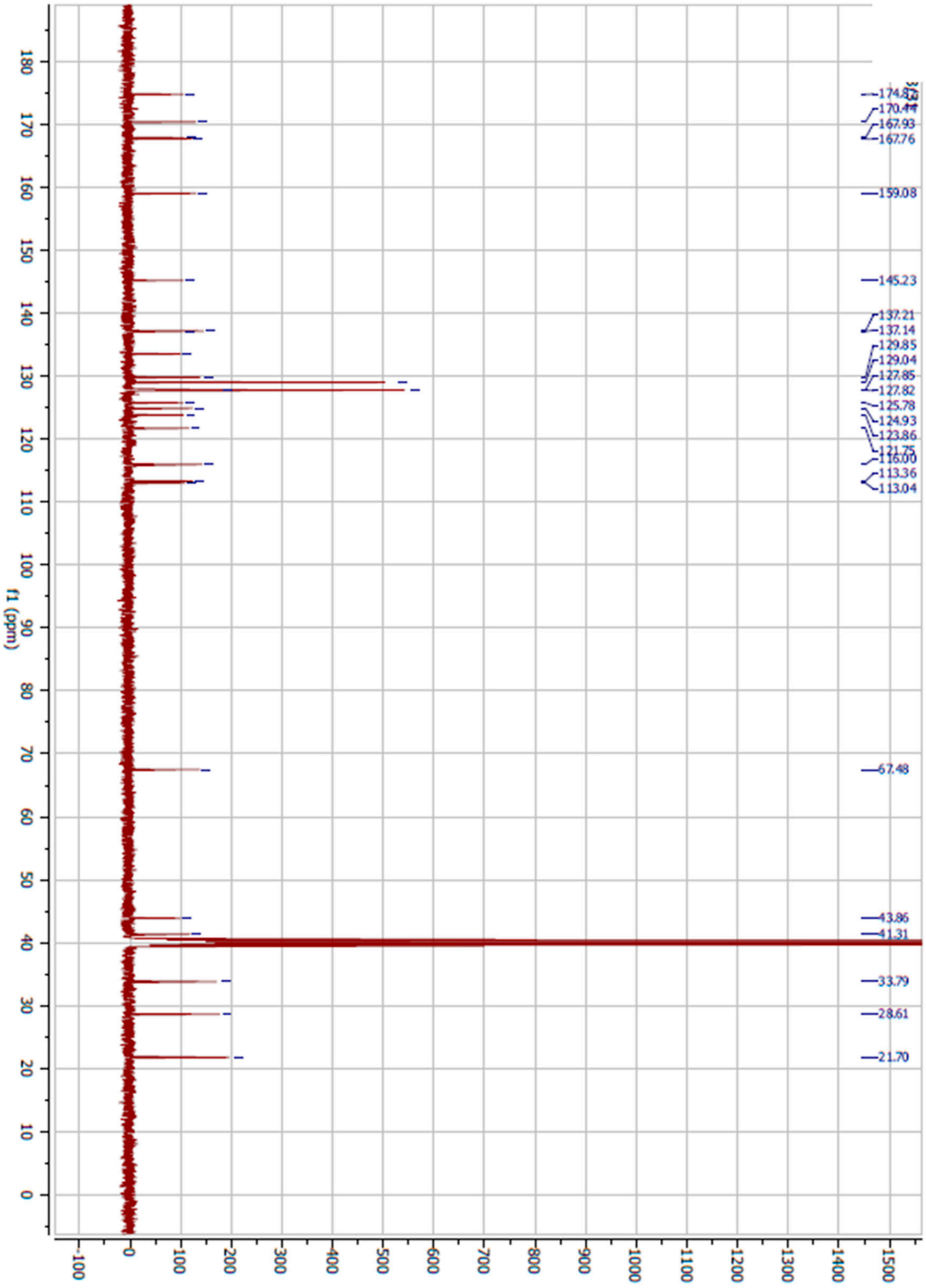

HRMS

5-(3-{[(2-benzyl-1,3-dioxo-2,3-dihydro-1H-isoindol-5-yl)carbamoyl]methyl}phenoxy)pentanoic acid (**3**; ZHAW5077)

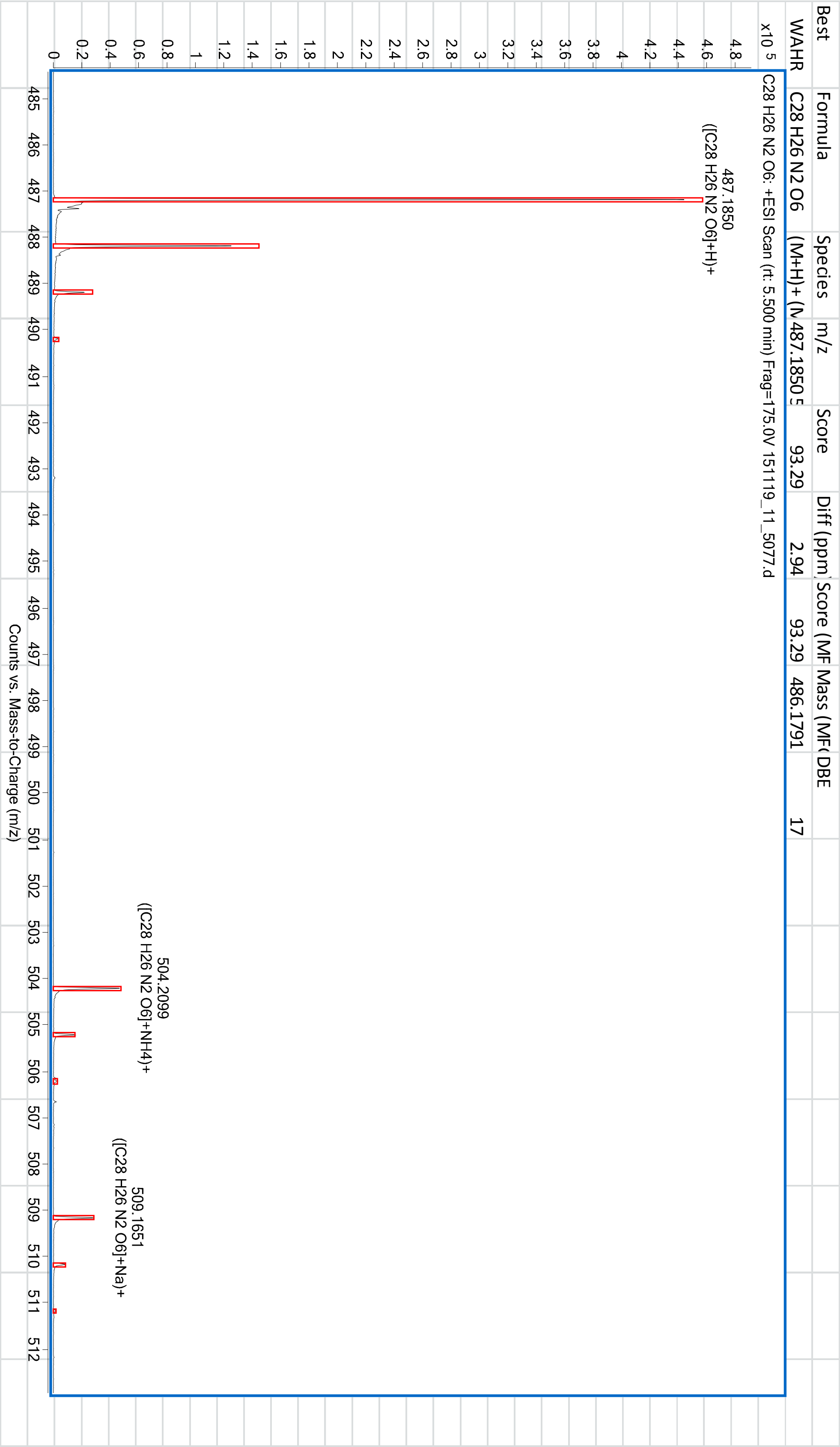

IC_{50}

5-(3-{[(2-benzyl-1,3-dioxo-2,3-dihydro-1H-isoindol-5-yl)carbamoyl]methyl}phenoxy)pentanoic acid (**3**; ZHAW5077)

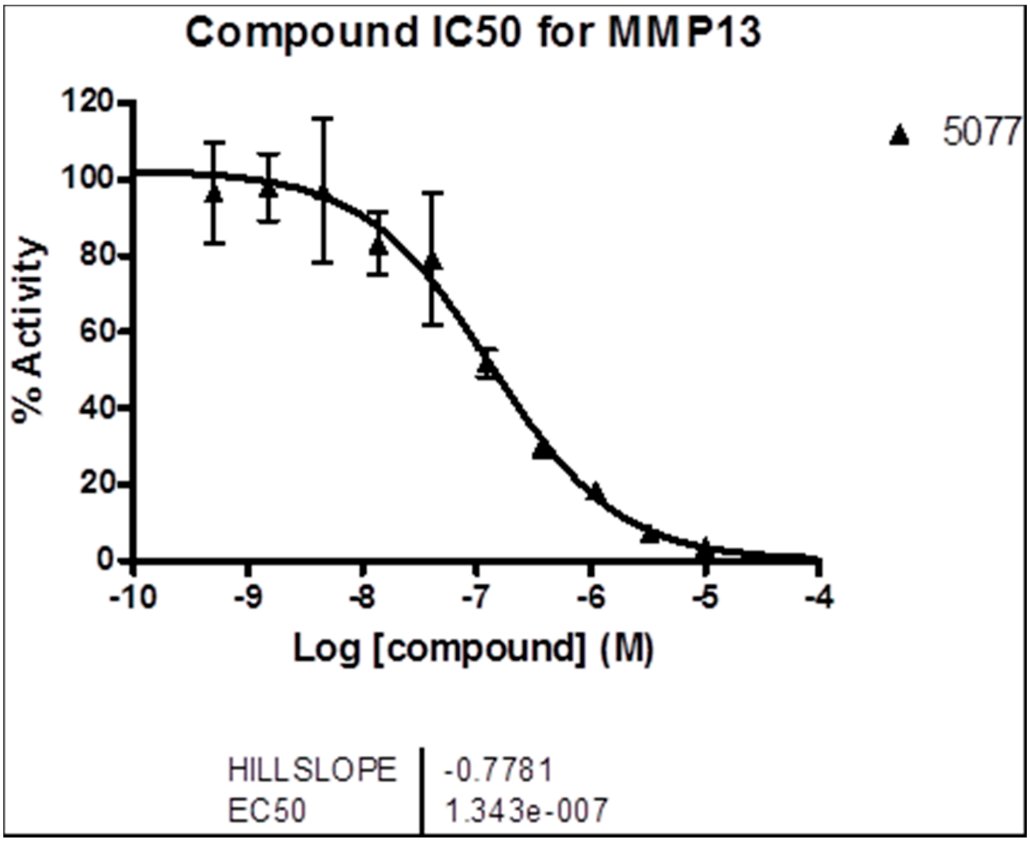

5-(3-{[(2-benzyl-1,3-dioxo-2,3-dihydro-1H-isoindol-5-yl)carbamoyl]methyl}phenoxy)-N-methanesulfonylpentanamide (**4**; ZHAW5079)

NMR

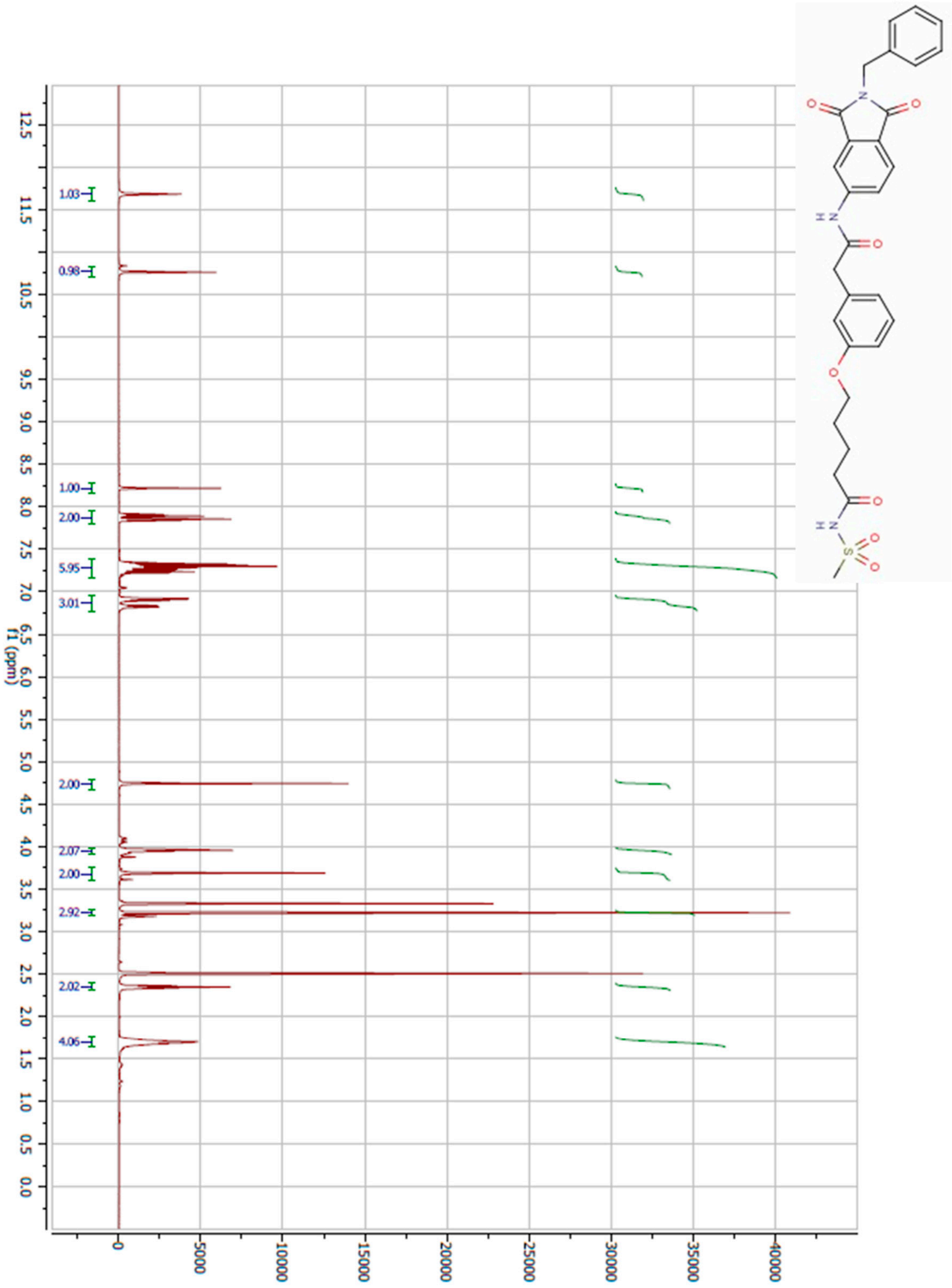

5-(3-{[(2-benzyl-1,3-dioxo-2,3-dihydro-1H-isoindol-5-yl)carbamoyl]methyl}phenoxy)-N-methanesulfonylpentanamide (**4**; ZHAW5079)

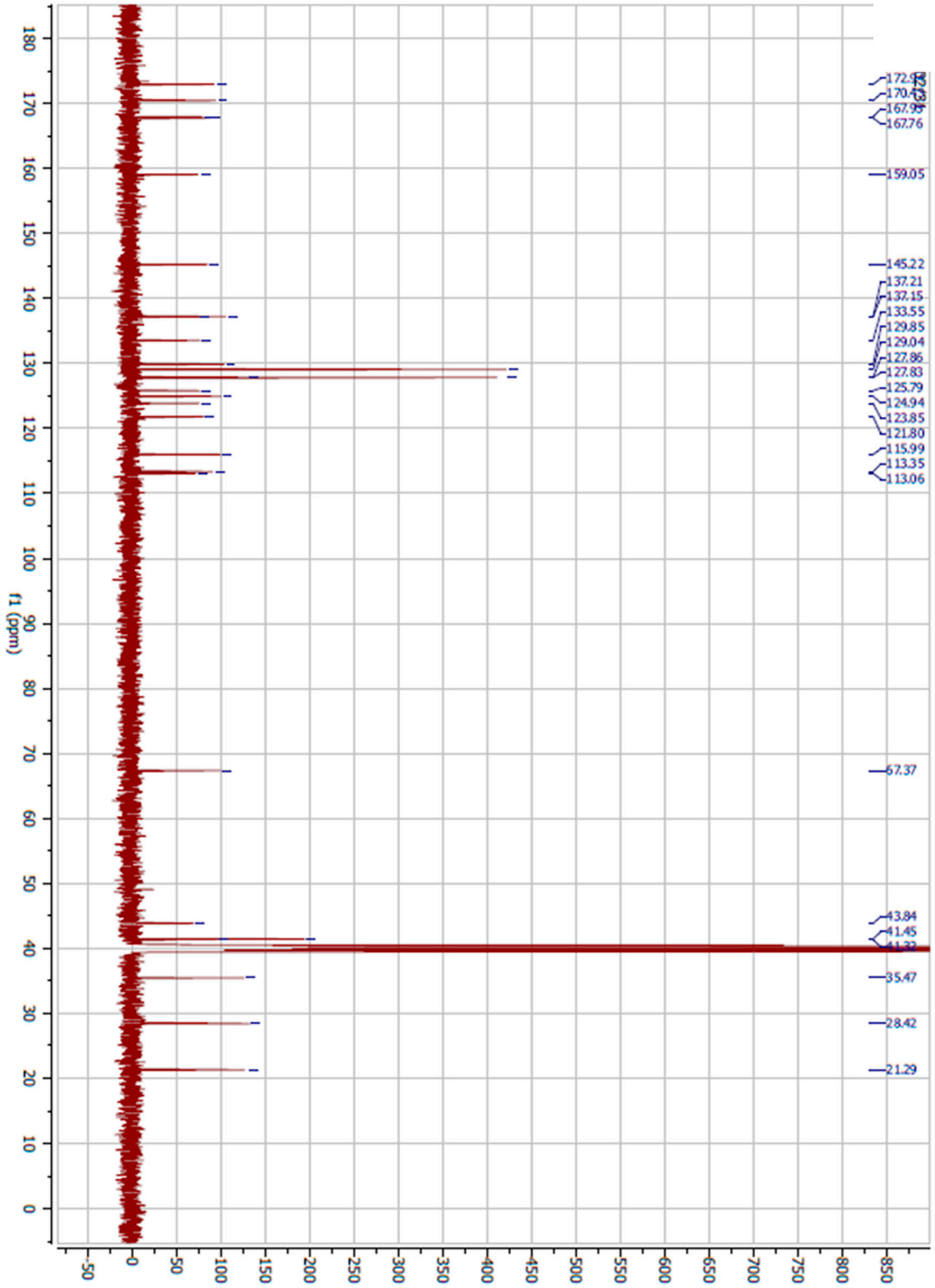

HRMS

5-(3-{[(2-benzyl-1,3-dioxo-2,3-dihydro-1H-isoindol-5-yl)carbamoyl]methyl}phenoxy)-N-methanesulfonylpentanamide (**4**; ZHAW5079)

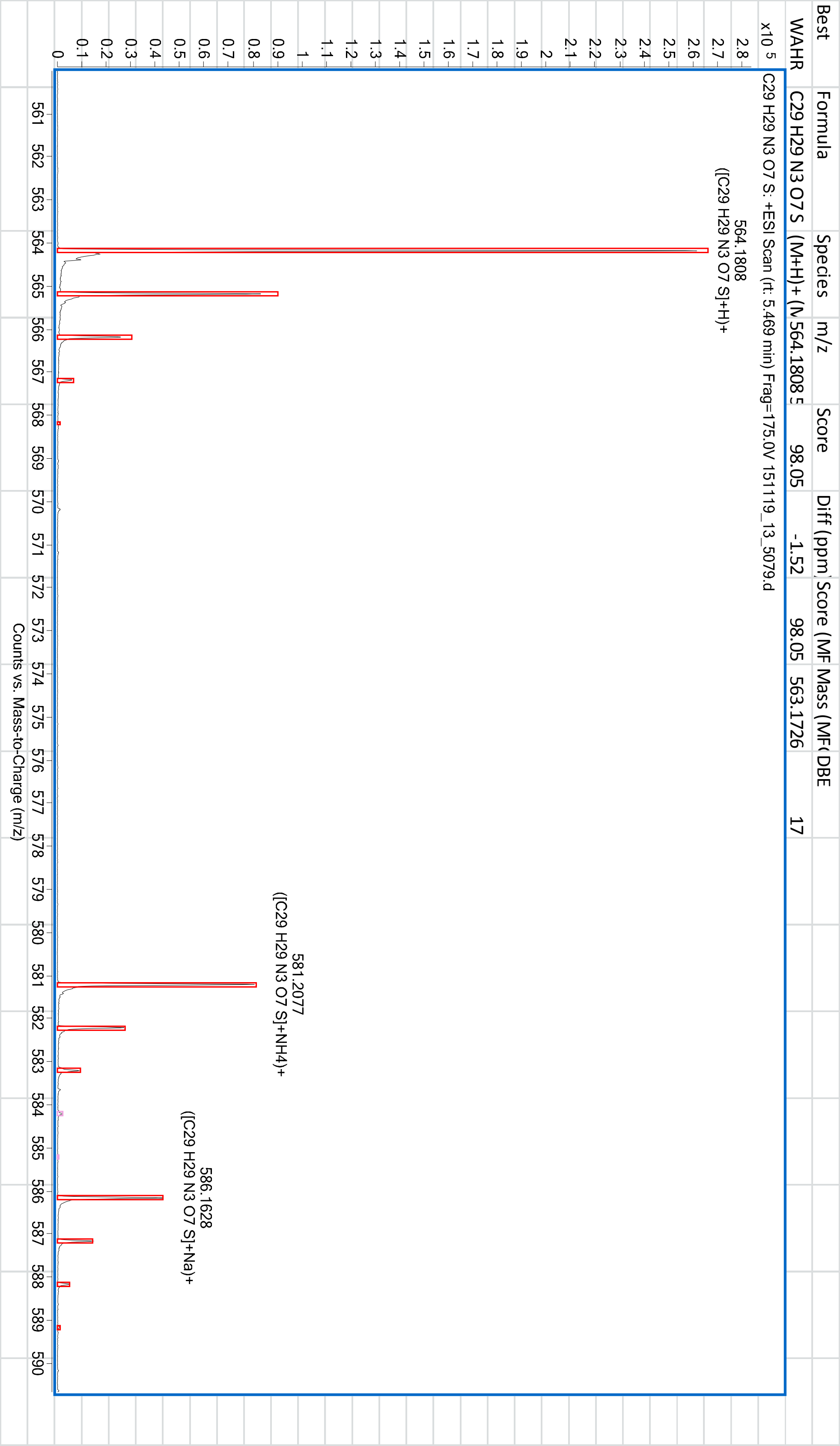

IC$_{50}$

5-(3-{[(2-benzyl-1,3-dioxo-2,3-dihydro-1H-isoindol-5-yl)carbamoyl]methyl}phenoxy)-N-methanesulfonylpentanamide (**4**; ZHAW5079)

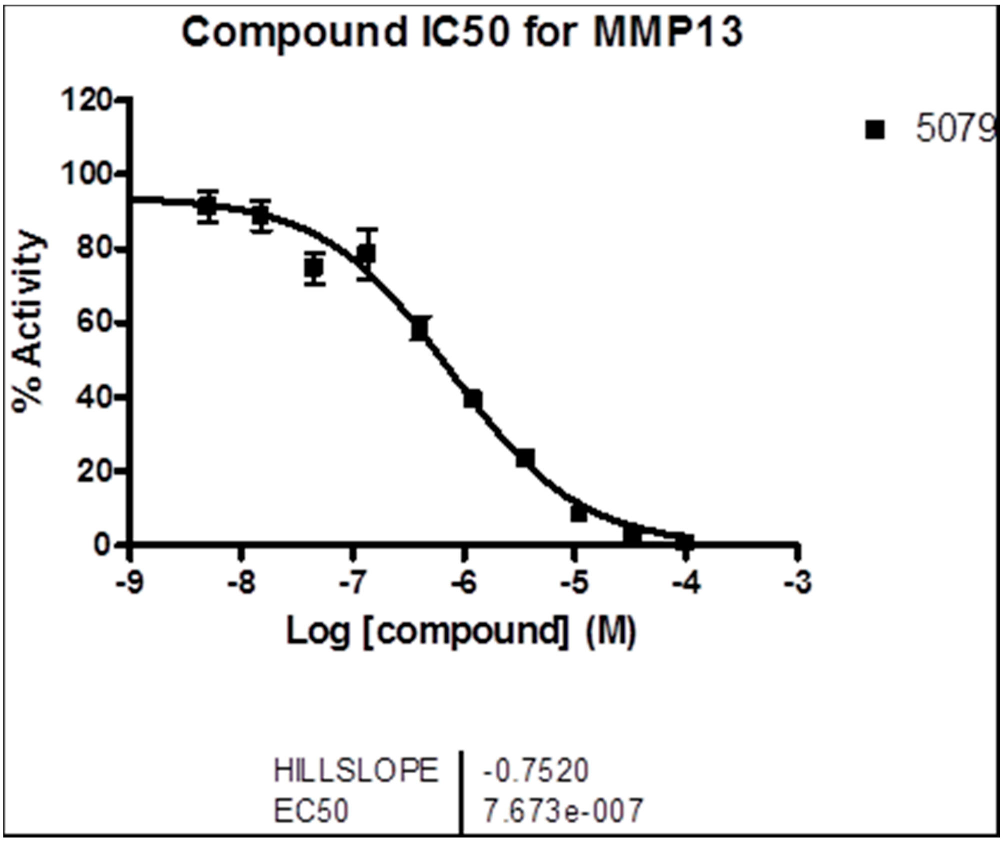

5-(3-{[(2-benzyl-1,3-dioxo-2,3-dihydro-1H-isoindol-5-yl)carbamoyl]methyl}phenoxy)-N-methylpentanamide (**5**; ZHAW5080)

NMR

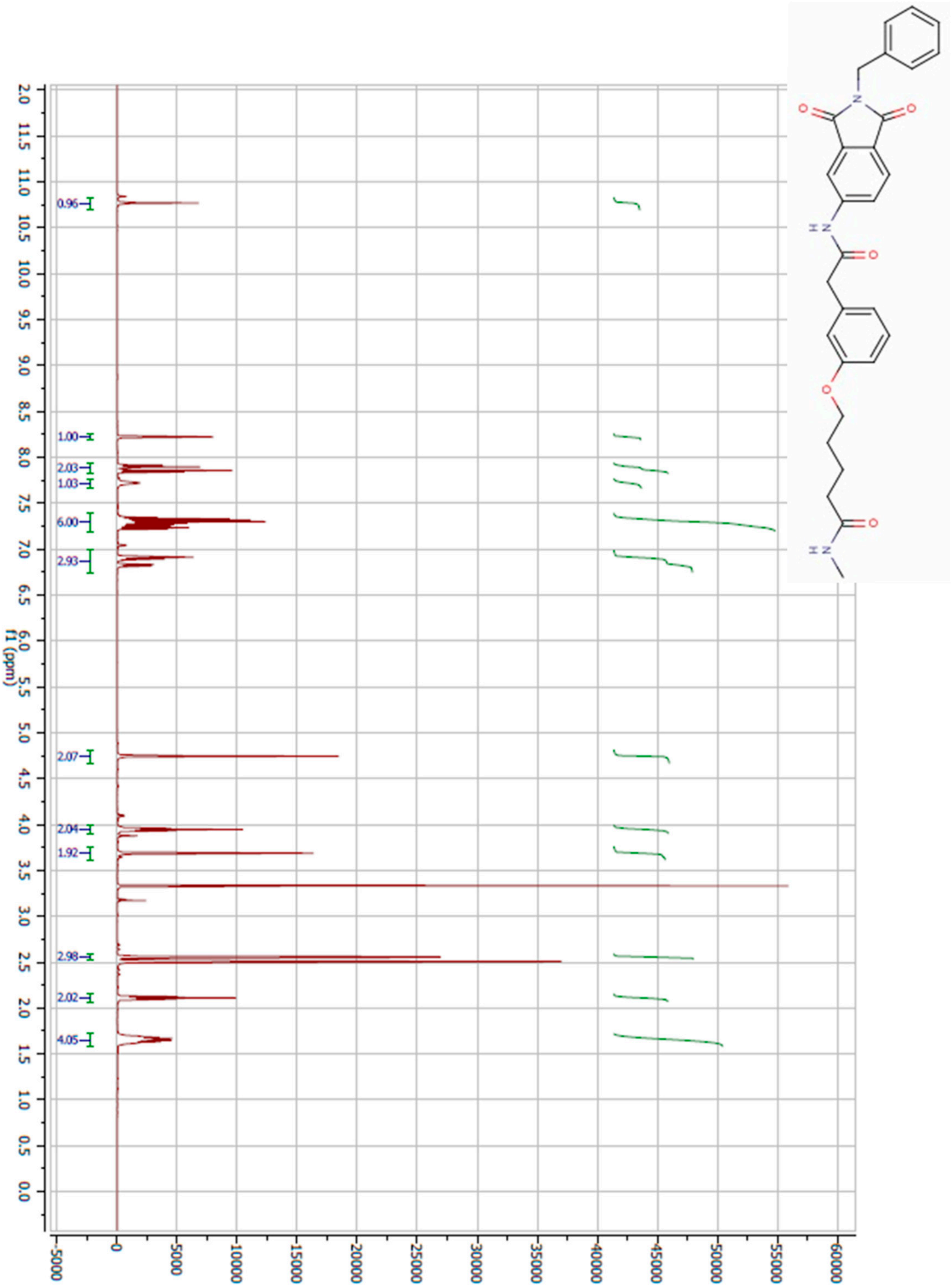

5-(3-{[(2-benzyl-1,3-dioxo-2,3-dihydro-1H-isoindol-5-yl)carbamoyl]methyl}phenoxy)-N-methylpentanamide (**5**; ZHAW5080)

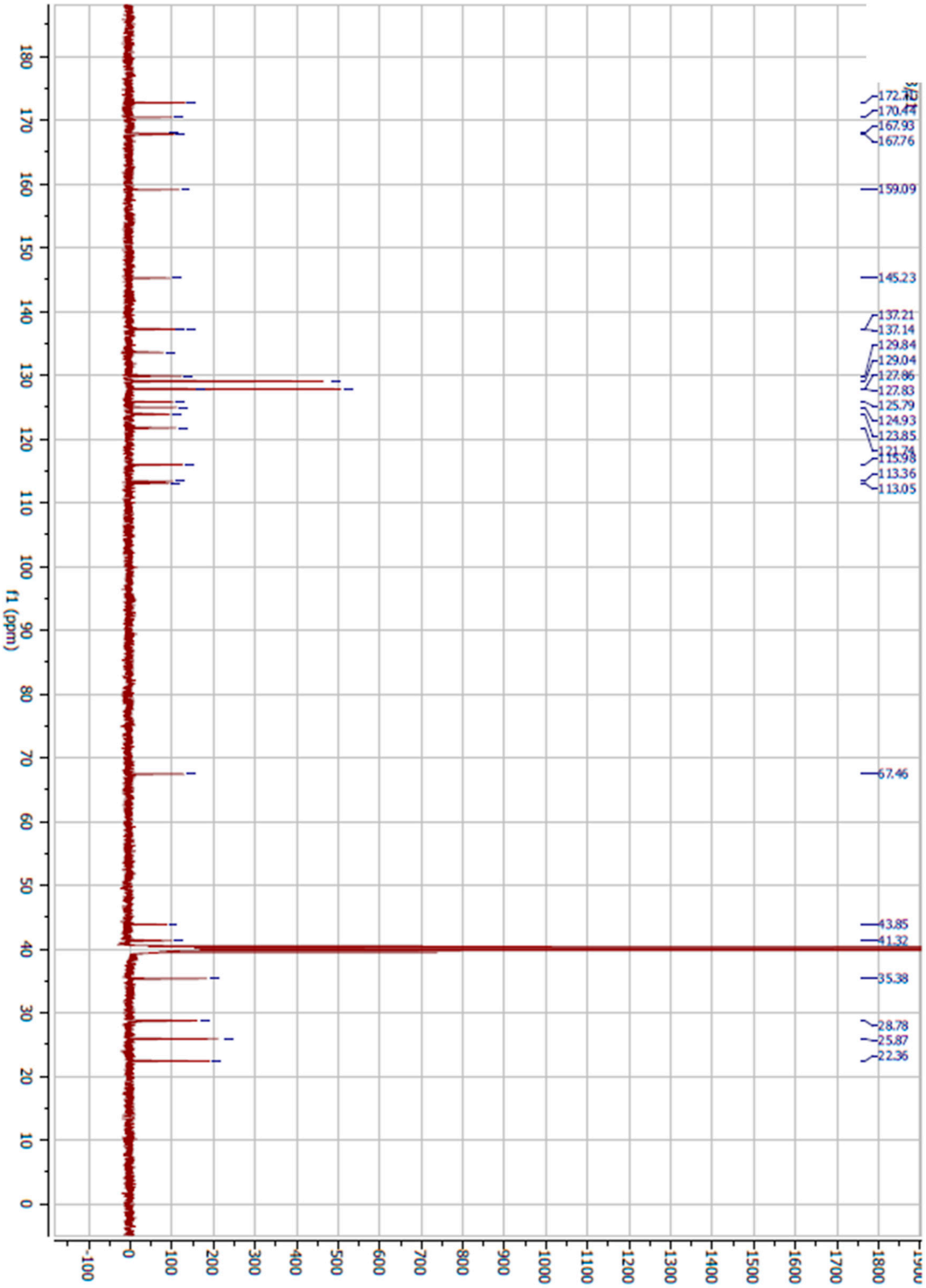

HRMS

5-(3-{[(2-benzyl-1,3-dioxo-2,3-dihydro-1H-isoindol-5-yl)carbamoyl]methyl}phenoxy)-N-methylpentanamide (**5**; ZHAW5080)

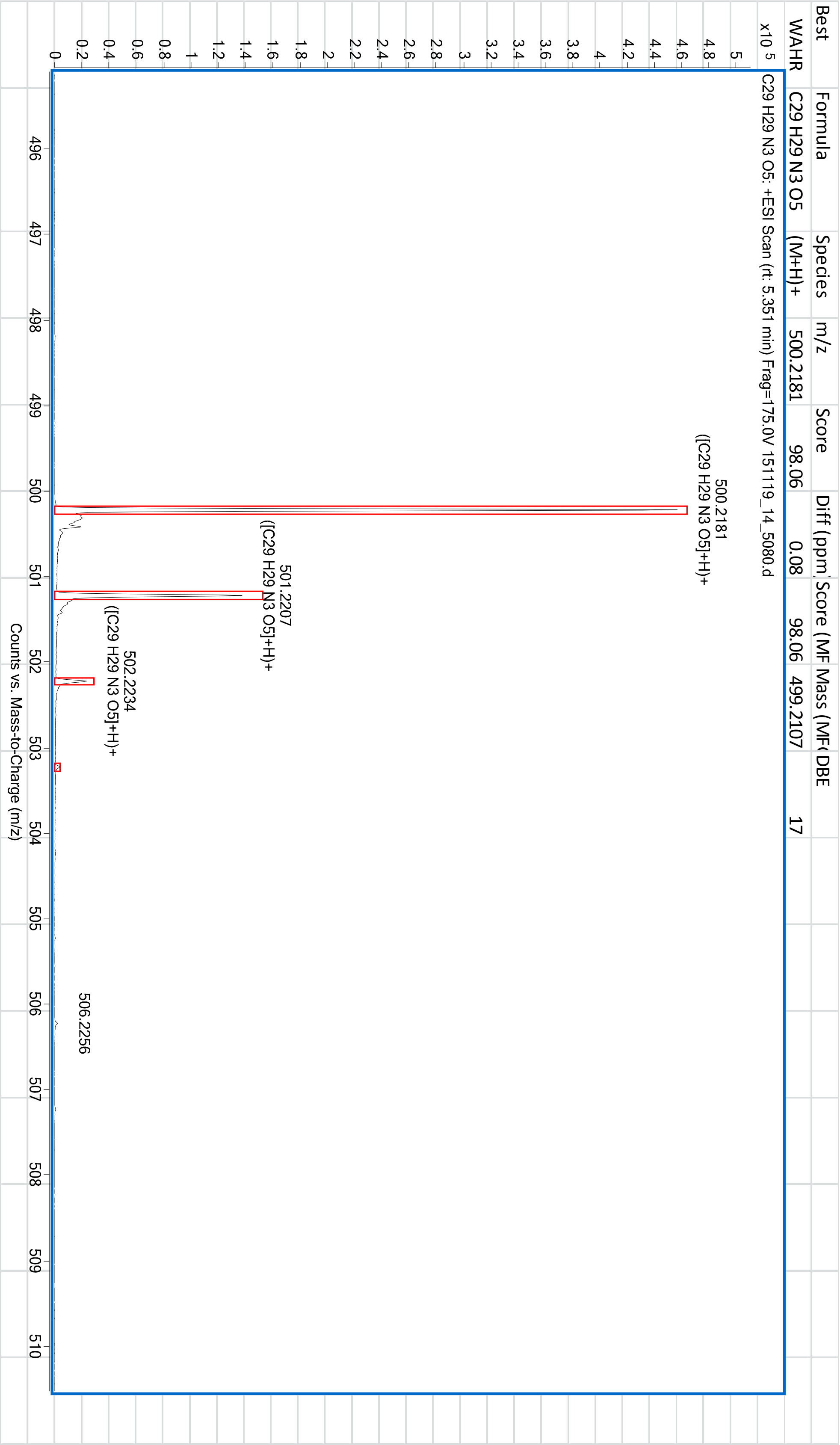

IC$_{50}$

5-(3-{[(2-benzyl-1,3-dioxo-2,3-dihydro-1H-isoindol-5-yl)carbamoyl]methyl}phenoxy)-N-methylpentanamide (**5**; ZHAW5080)

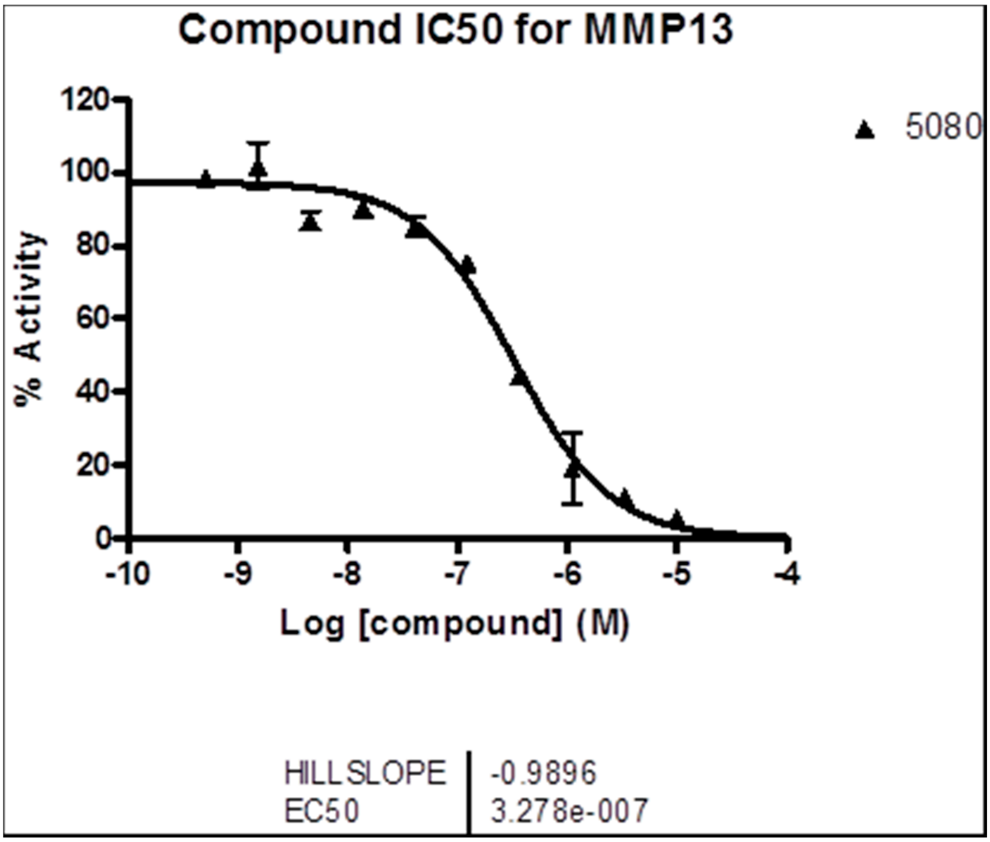

Methyl 2-(3-{[3-(benzyloxy)propyl]oxy}phenyl)acetate (**8a**; ZHAW4557)

NMR

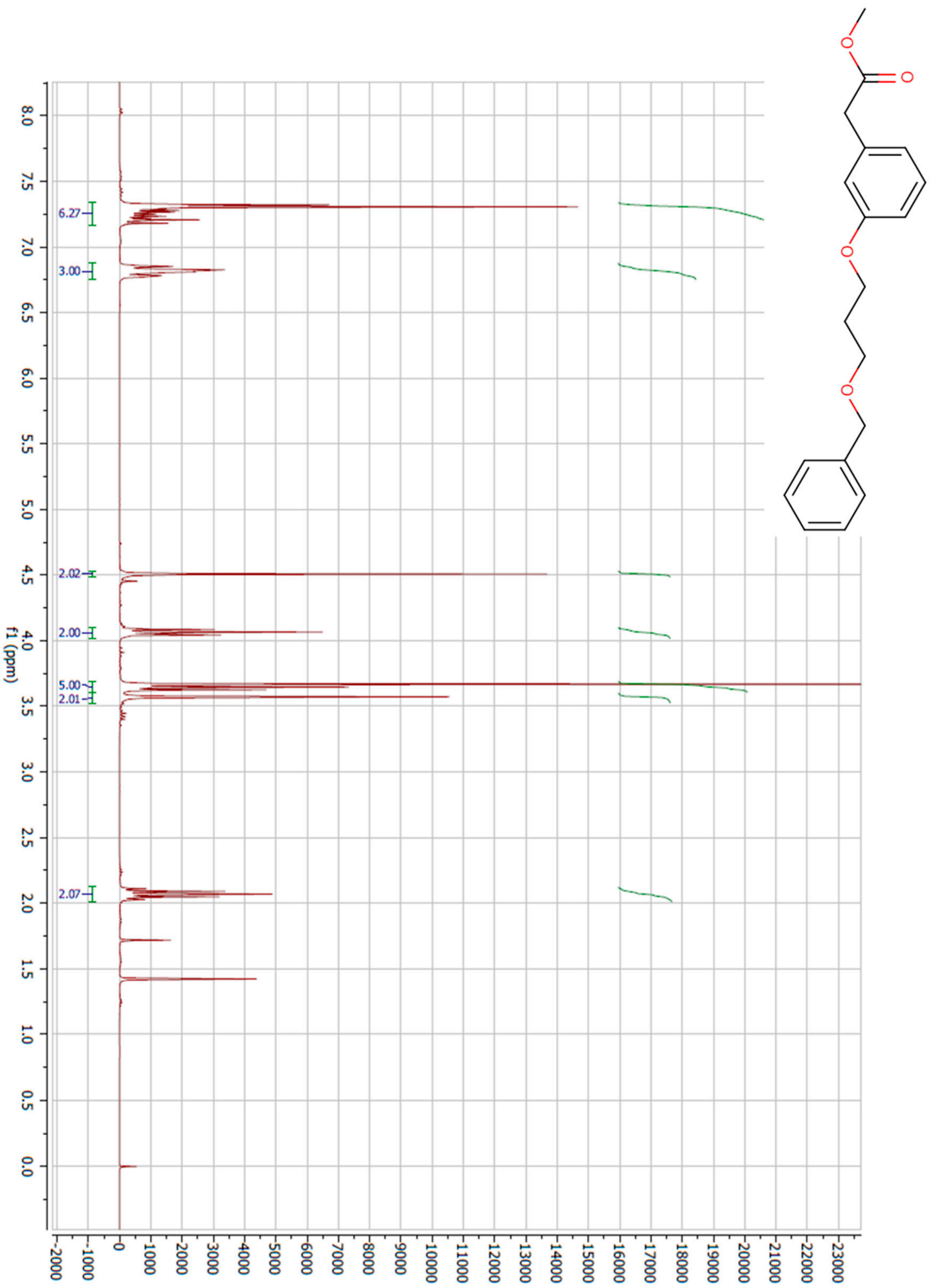

Methyl 2-(3-{[3-(benzyloxy)propyl]oxy}phenyl)acetate (**8a**; ZHAW4557)

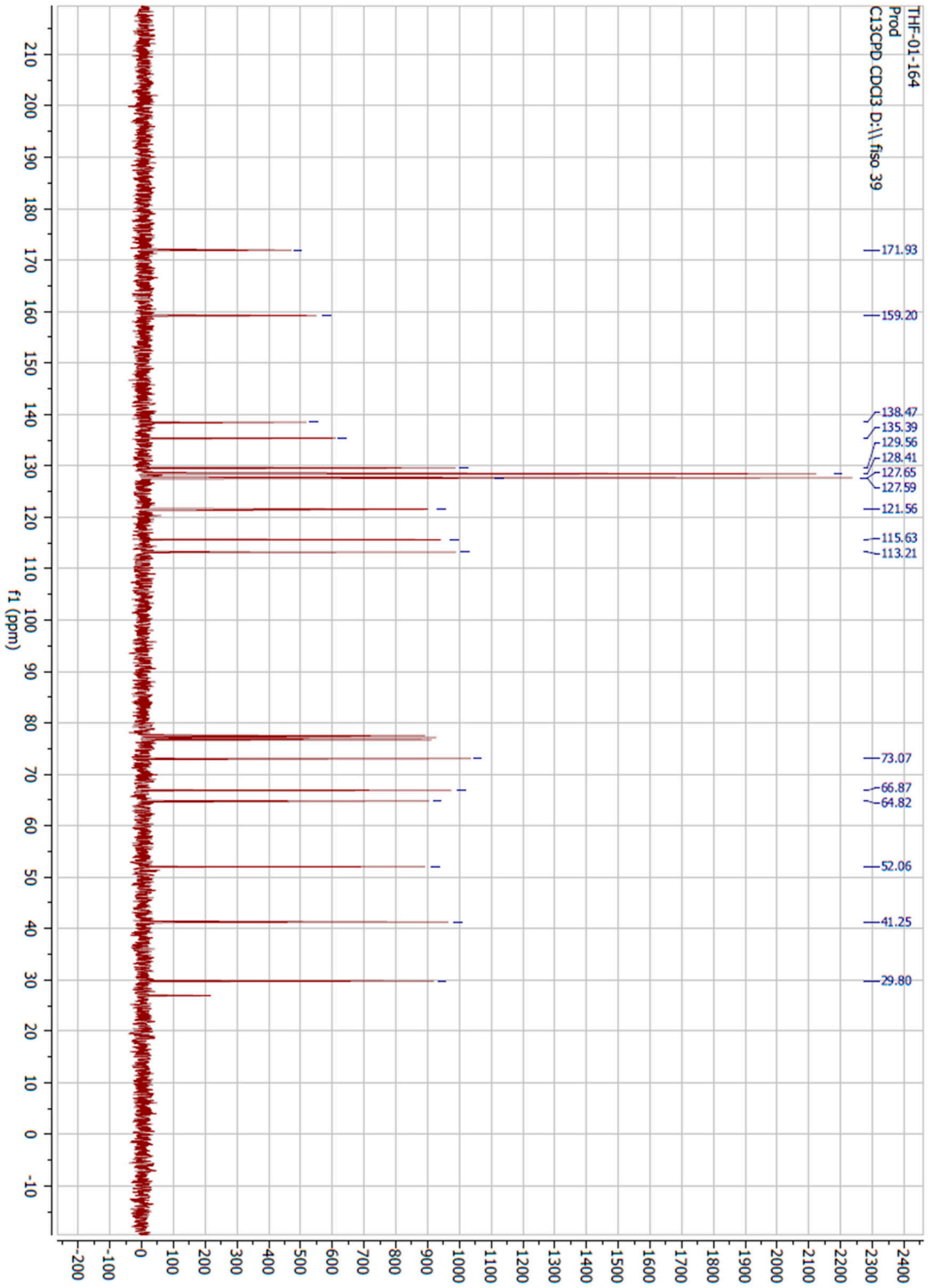

Methyl 2-(3-{[4-(benzyloxy)butyl]oxy}phenyl)acetate (**8b**; ZHAW4558)

NMR

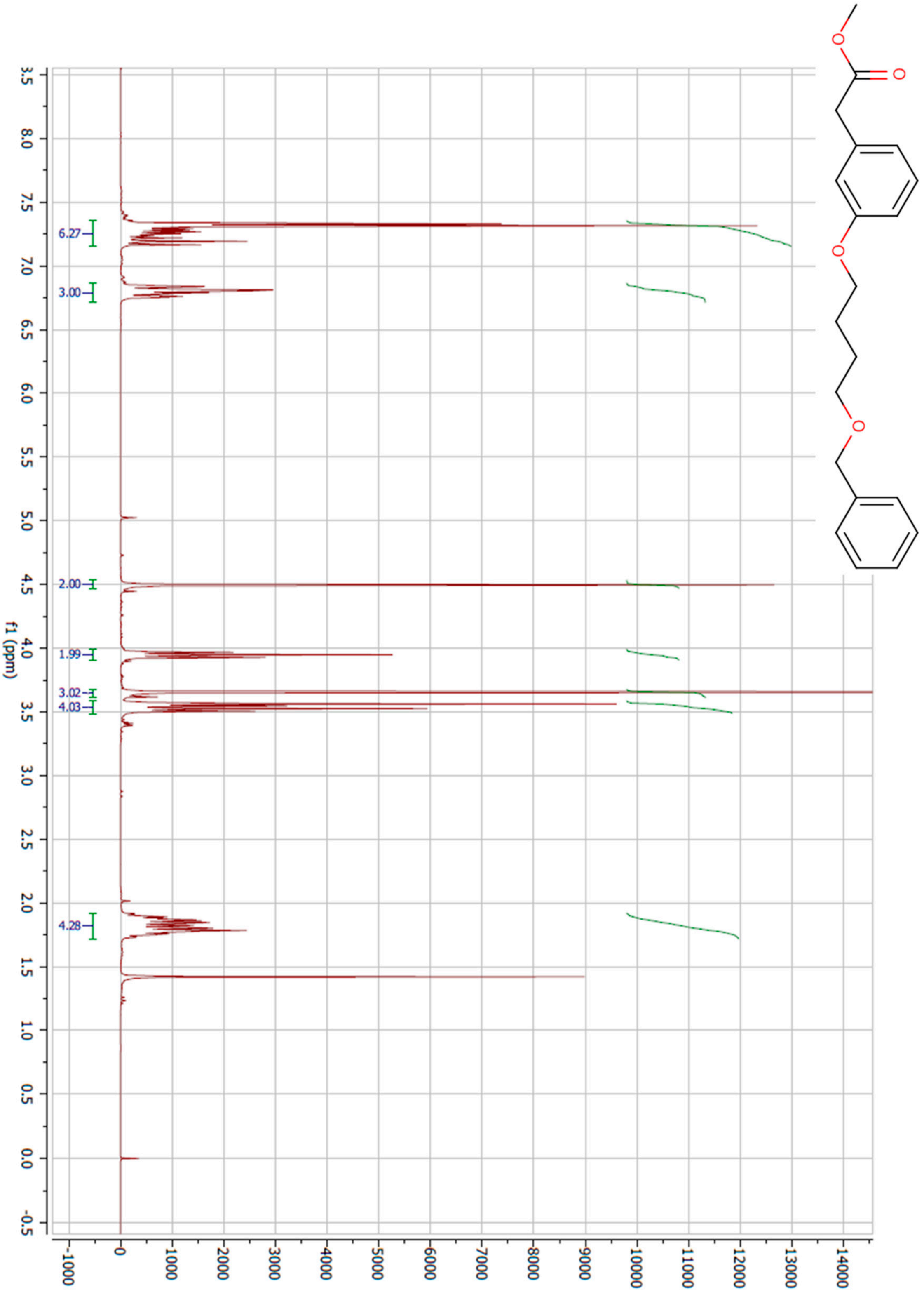

Methyl 2-(3-{[4-(benzyloxy)butyl]oxy}phenyl)acetate (**8b**; ZHAW4558)

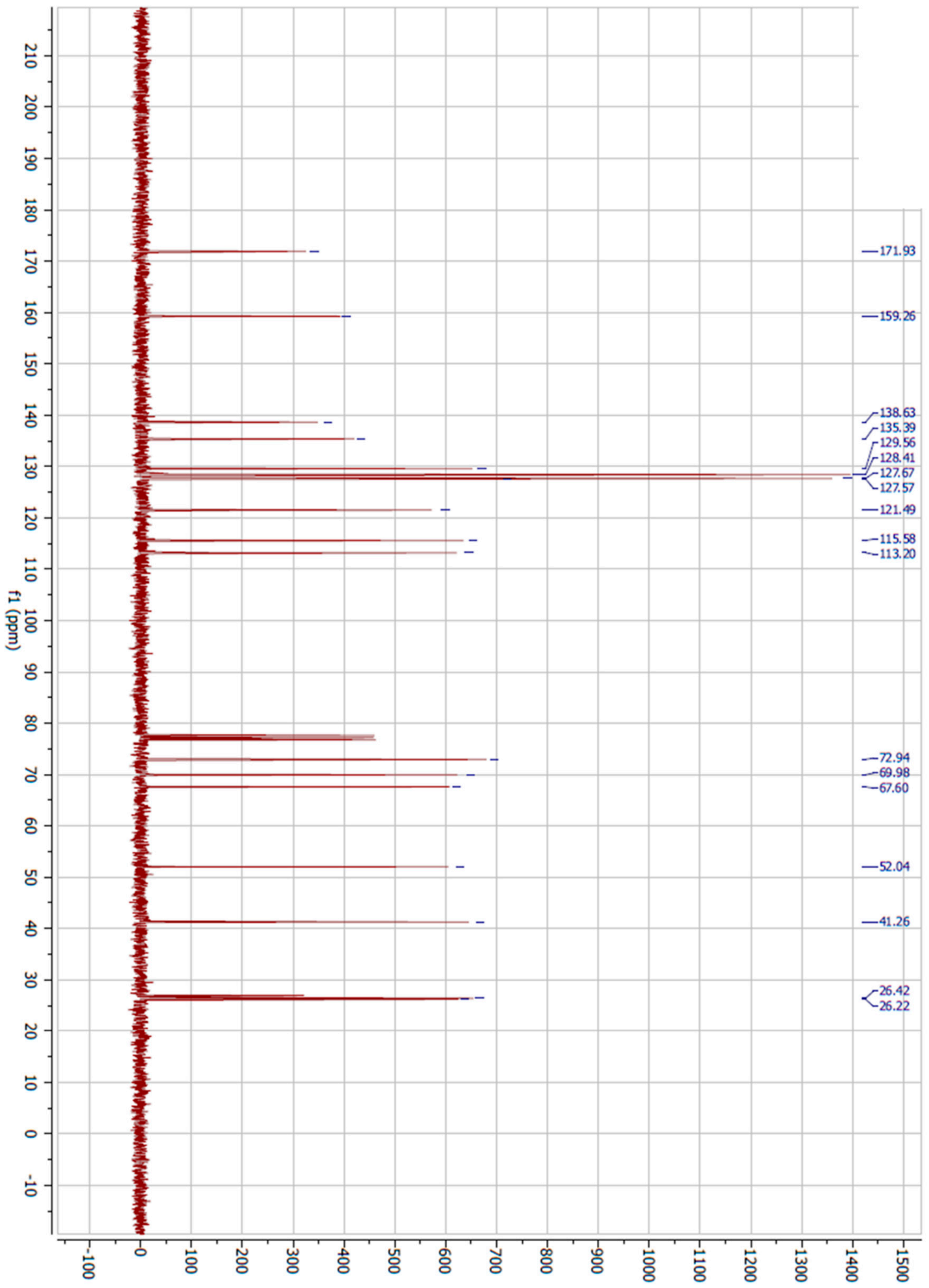

Methyl 2-(3-{[5-(benzyloxy)pentyl]oxy}phenyl)acetate (**8c**; ZHAW4927)

NMR

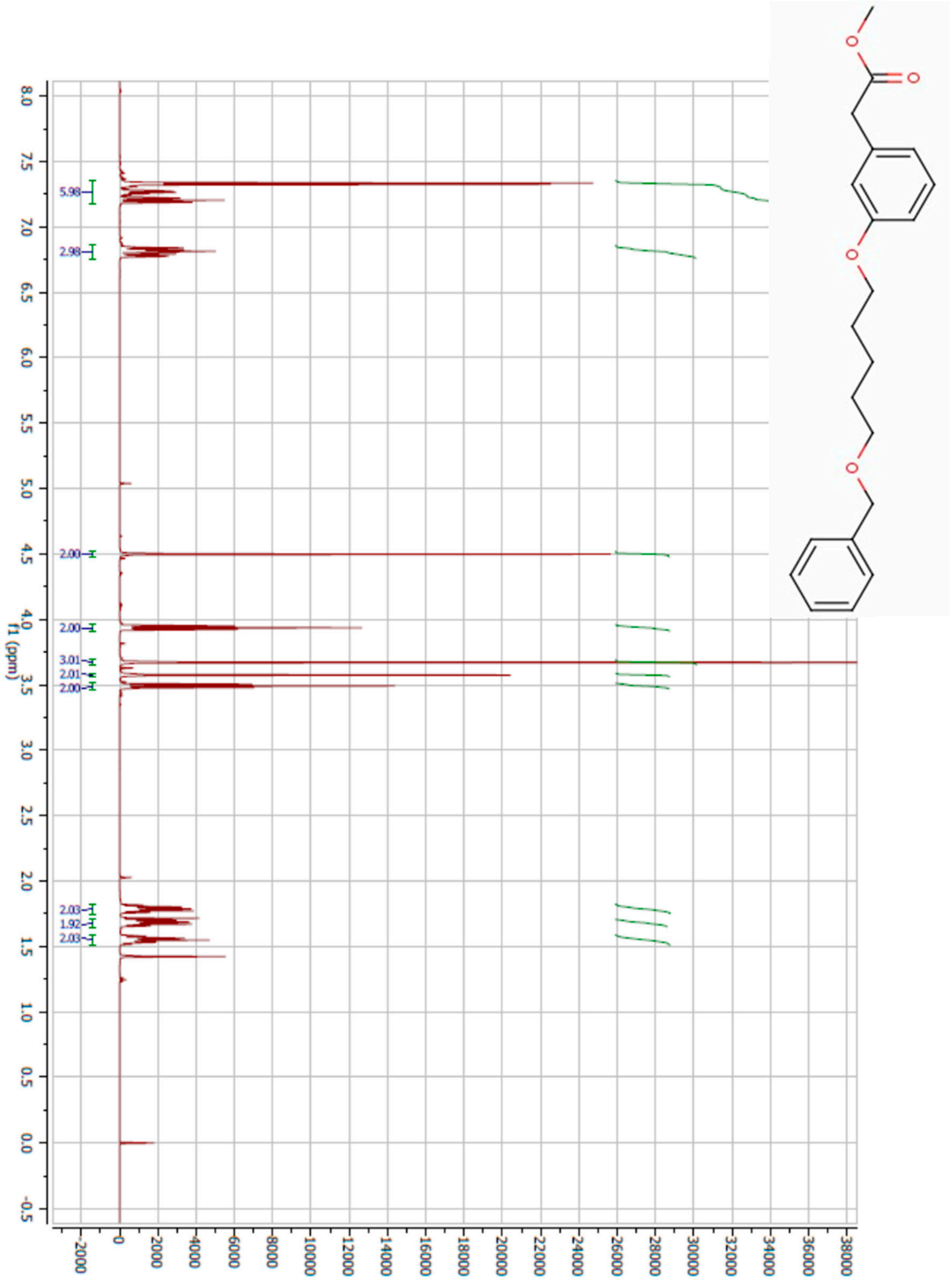

Methyl 2-(3-{[5-(benzyloxy)pentyl]oxy}phenyl)acetate (**8c**; ZHAW4927)

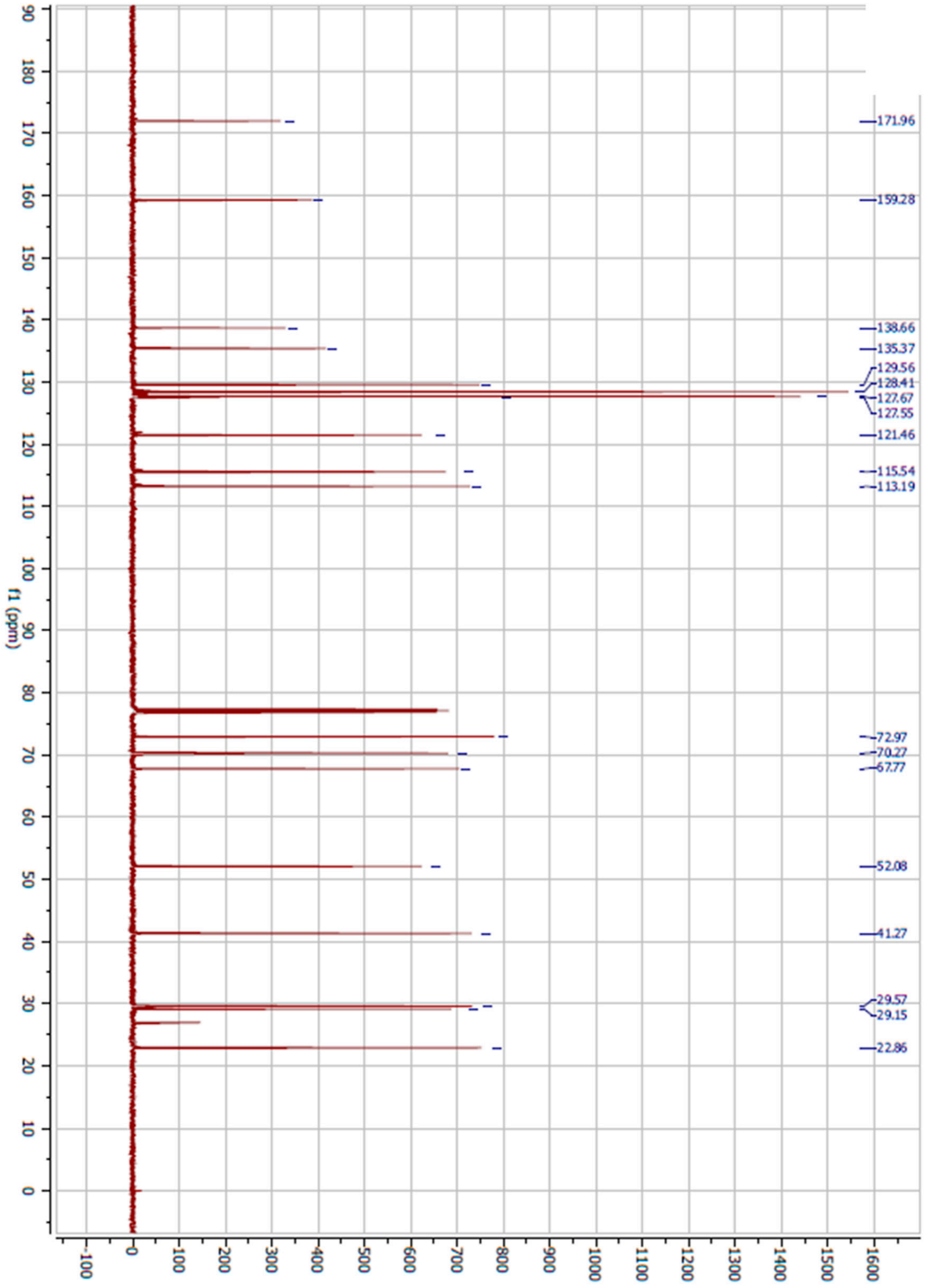

HRMS

Methyl 2-(3-{[5-(benzyloxy)pentyl]oxy}phenyl)acetate (**8c**; ZHAW4927)

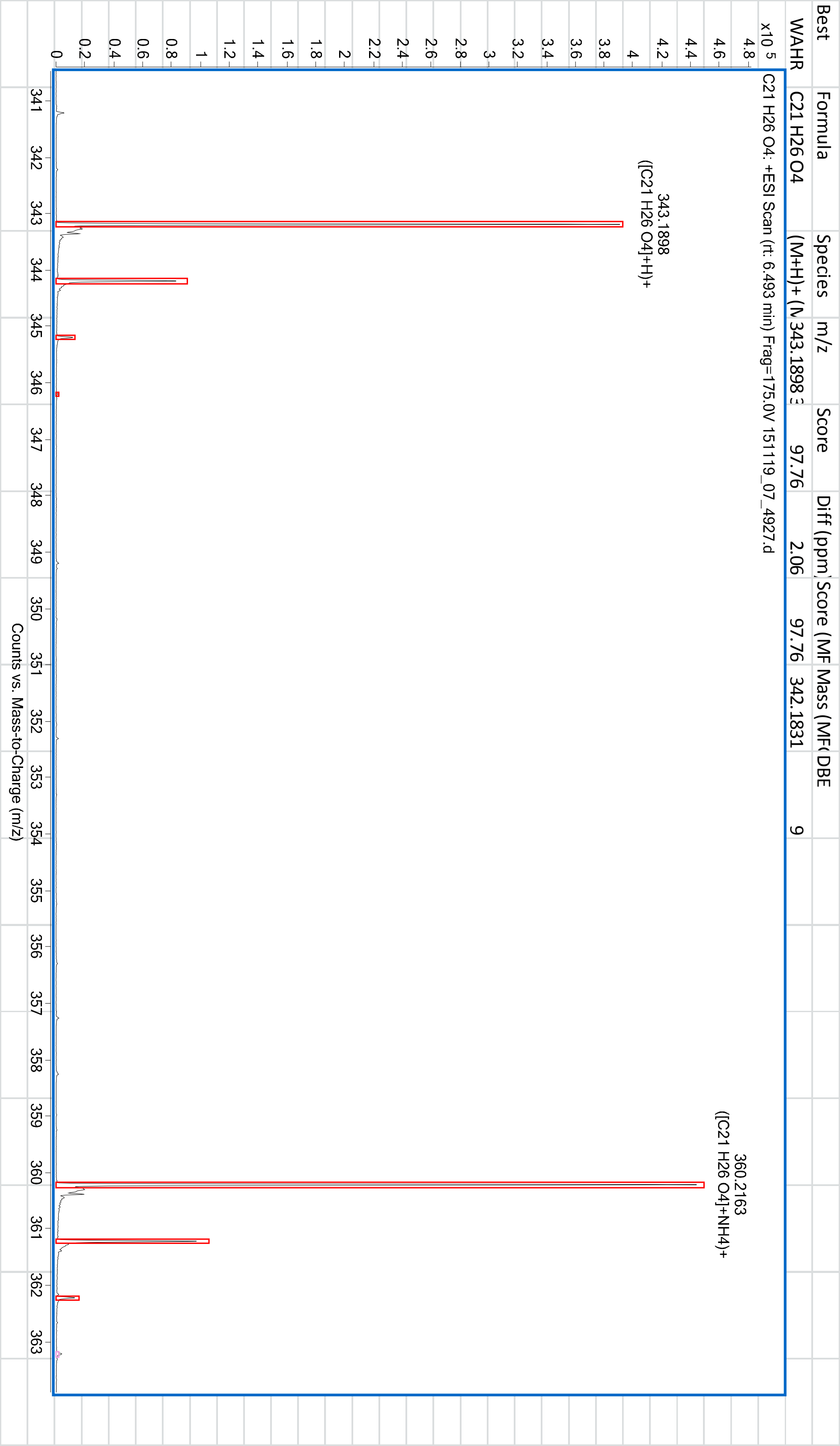

Methyl 2-(3-{[6-(benzyloxy)hexyl]oxy}phenyl)acetate (**8d**; ZHAW4928)

NMR

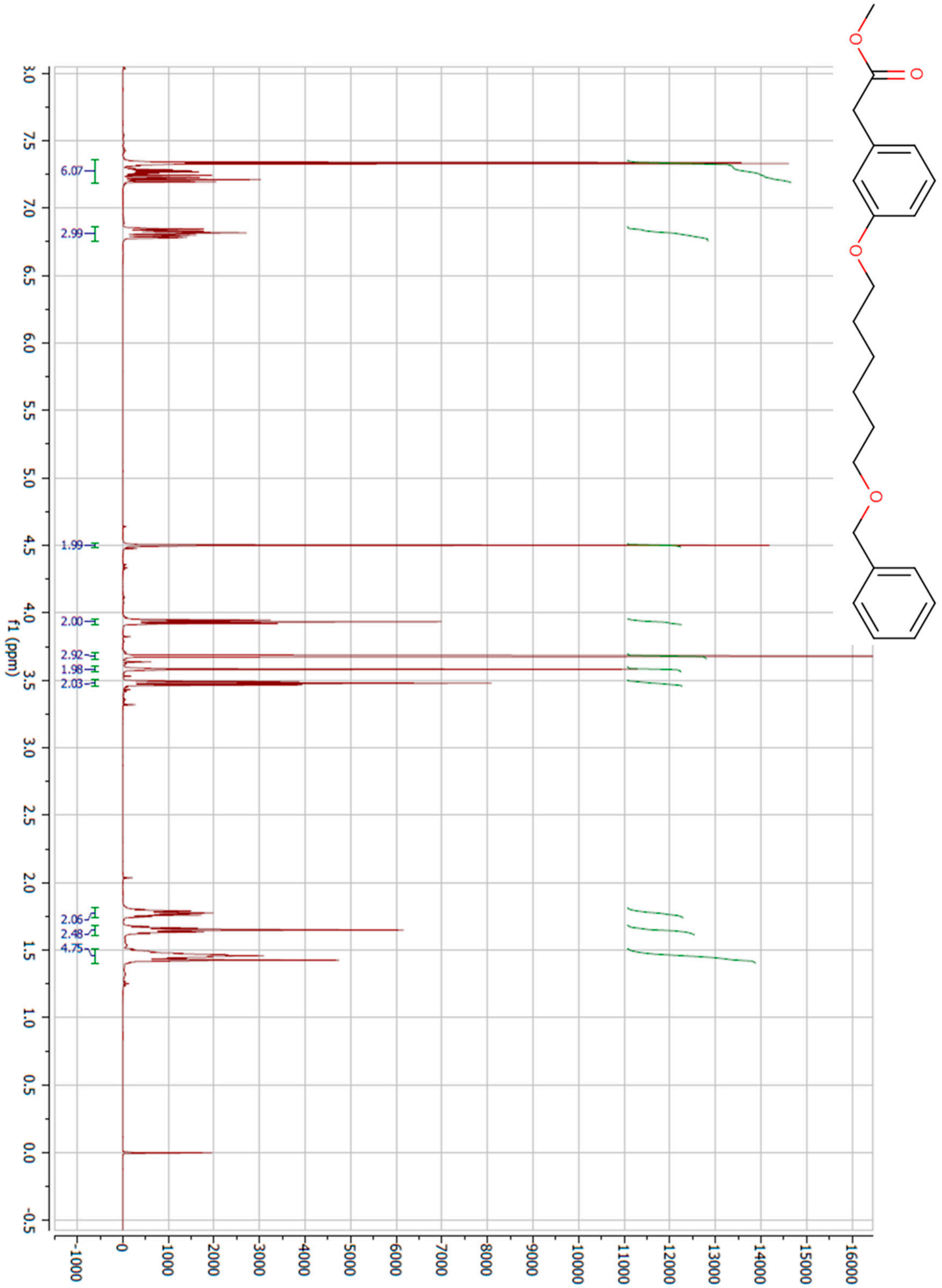

Methyl 2-(3-{[6-(benzyloxy)hexyl]oxy}phenyl)acetate (**8d**; ZHAW4928)

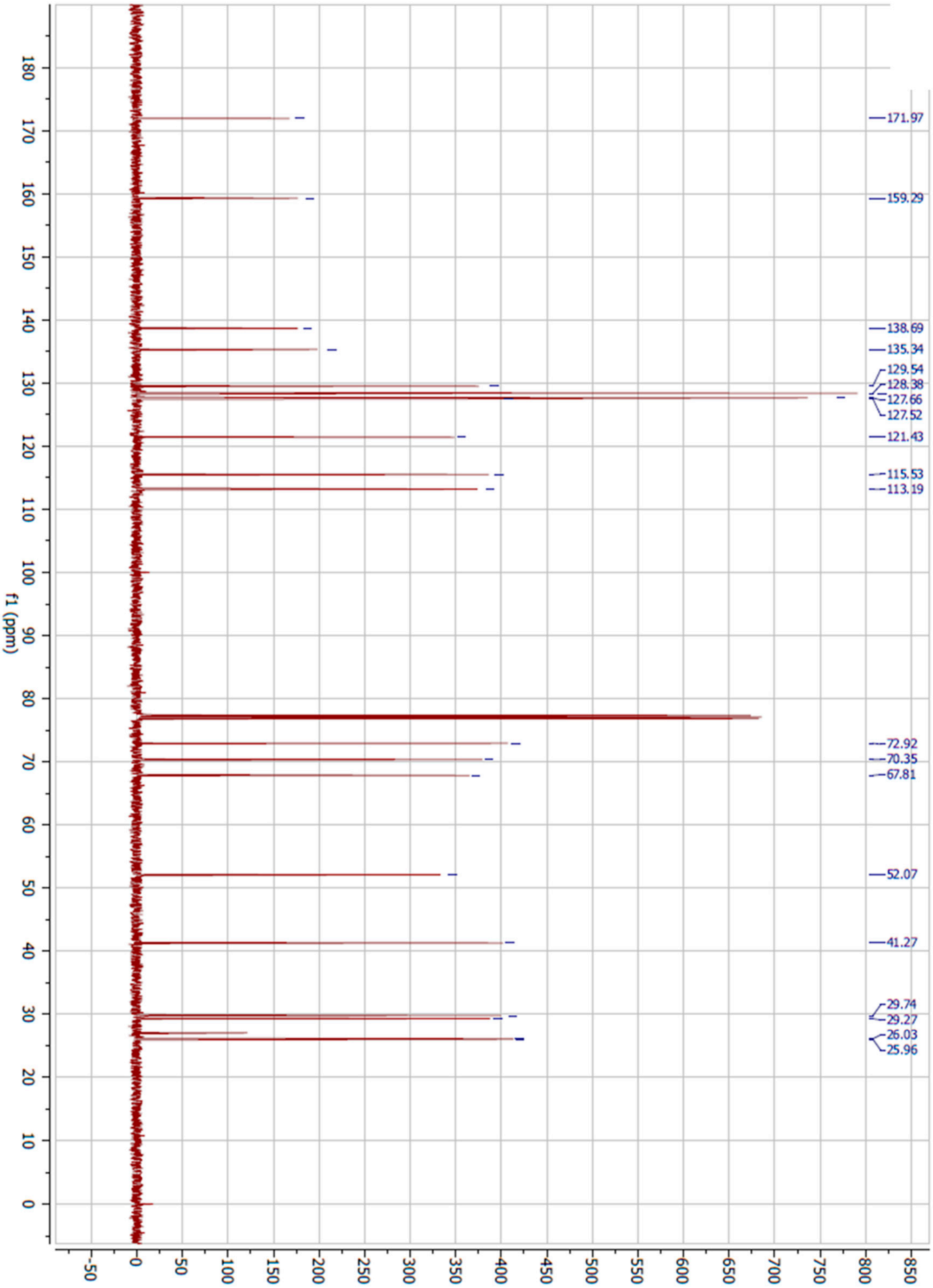

2-(3-{[3-(benzyloxy)propyl]oxy}phenyl)acetic acid (**9a**; ZHAW4559)

NMR

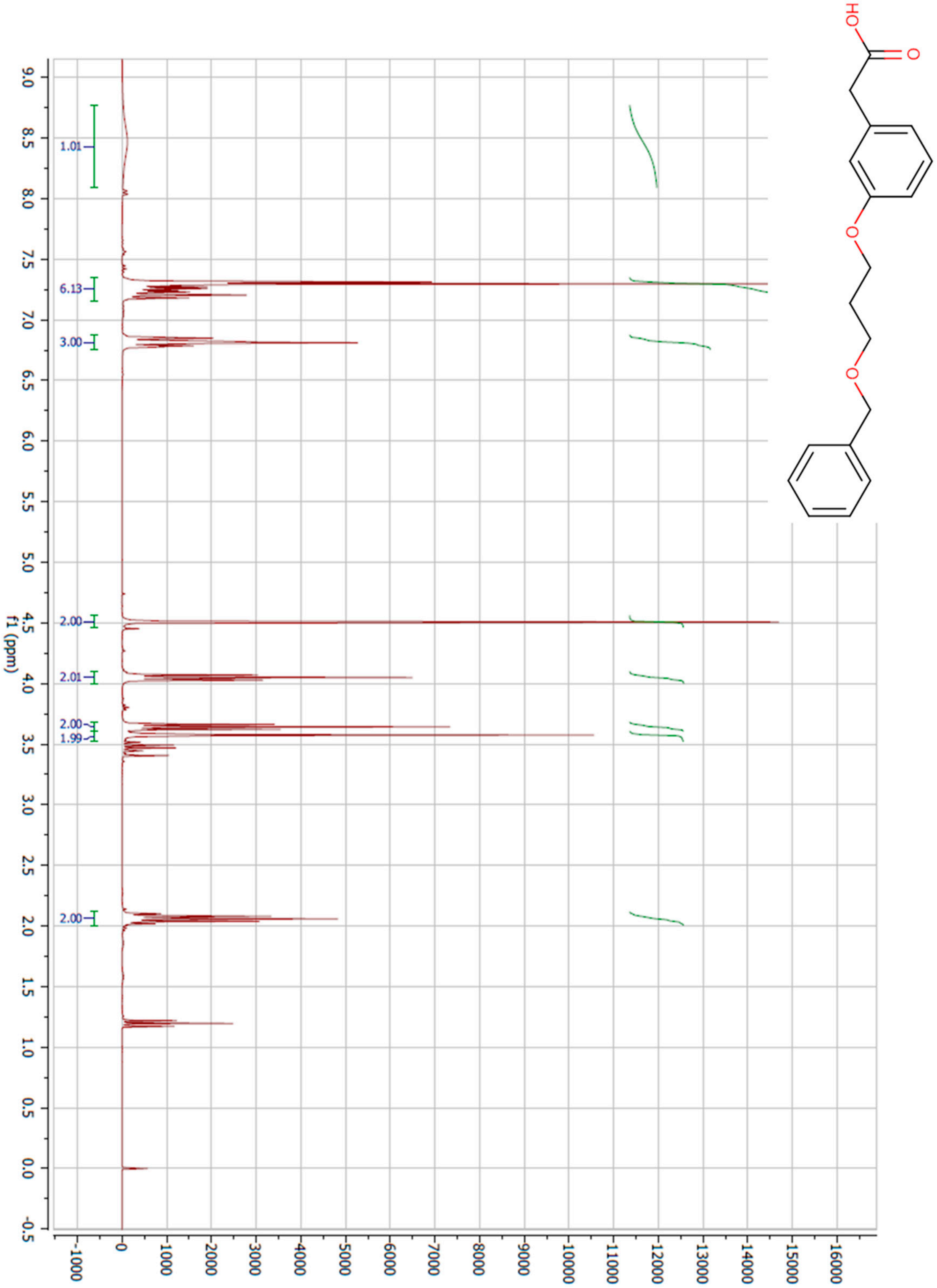

2-(3-{[3-(benzyloxy)propyl]oxy}phenyl)acetic acid (**9a**; ZHAW4559)

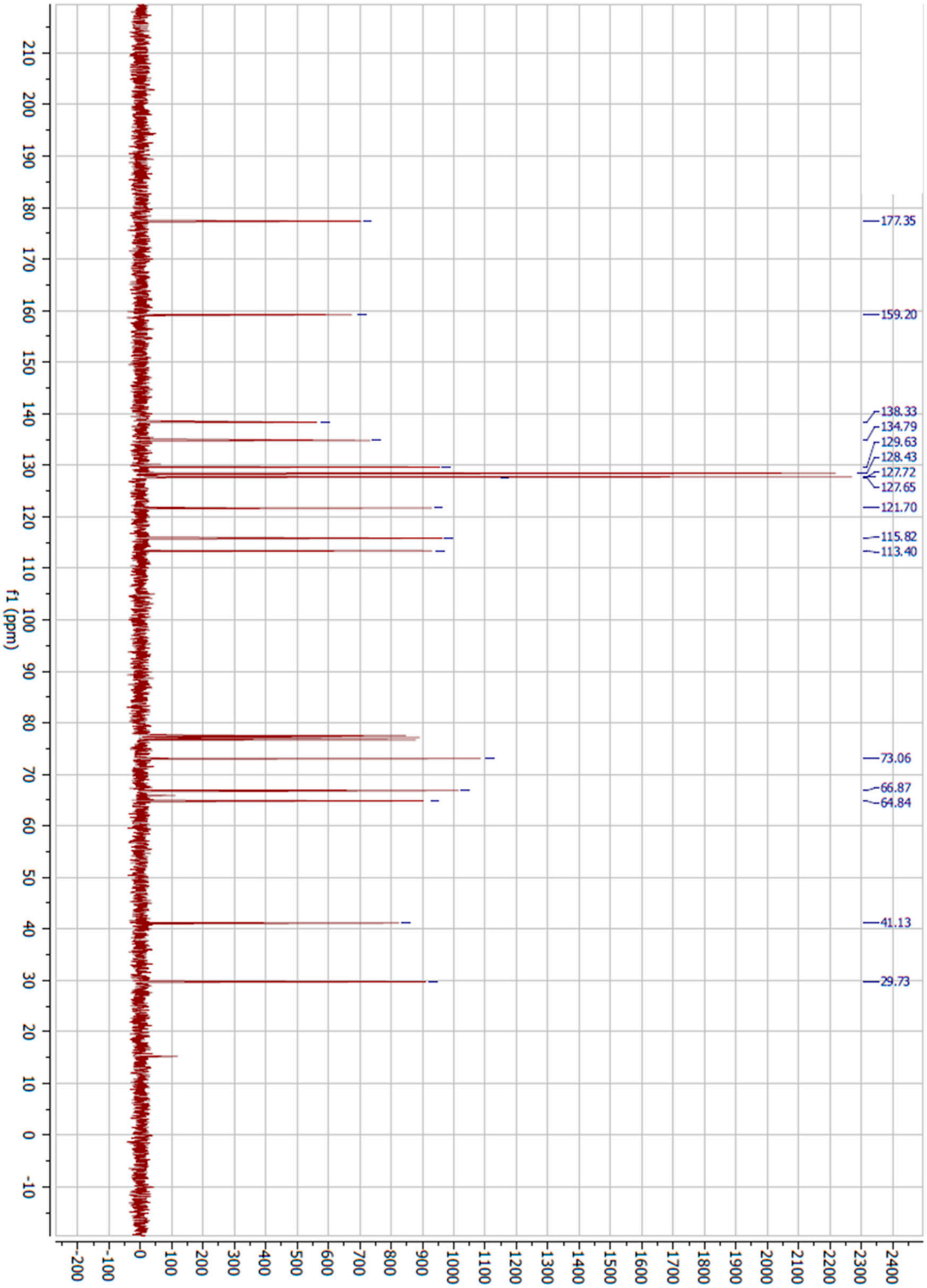

2-(3-{[4-(benzyloxy)butyl]oxy}phenyl)acetic acid (**9b**; ZHAW4560)

NMR

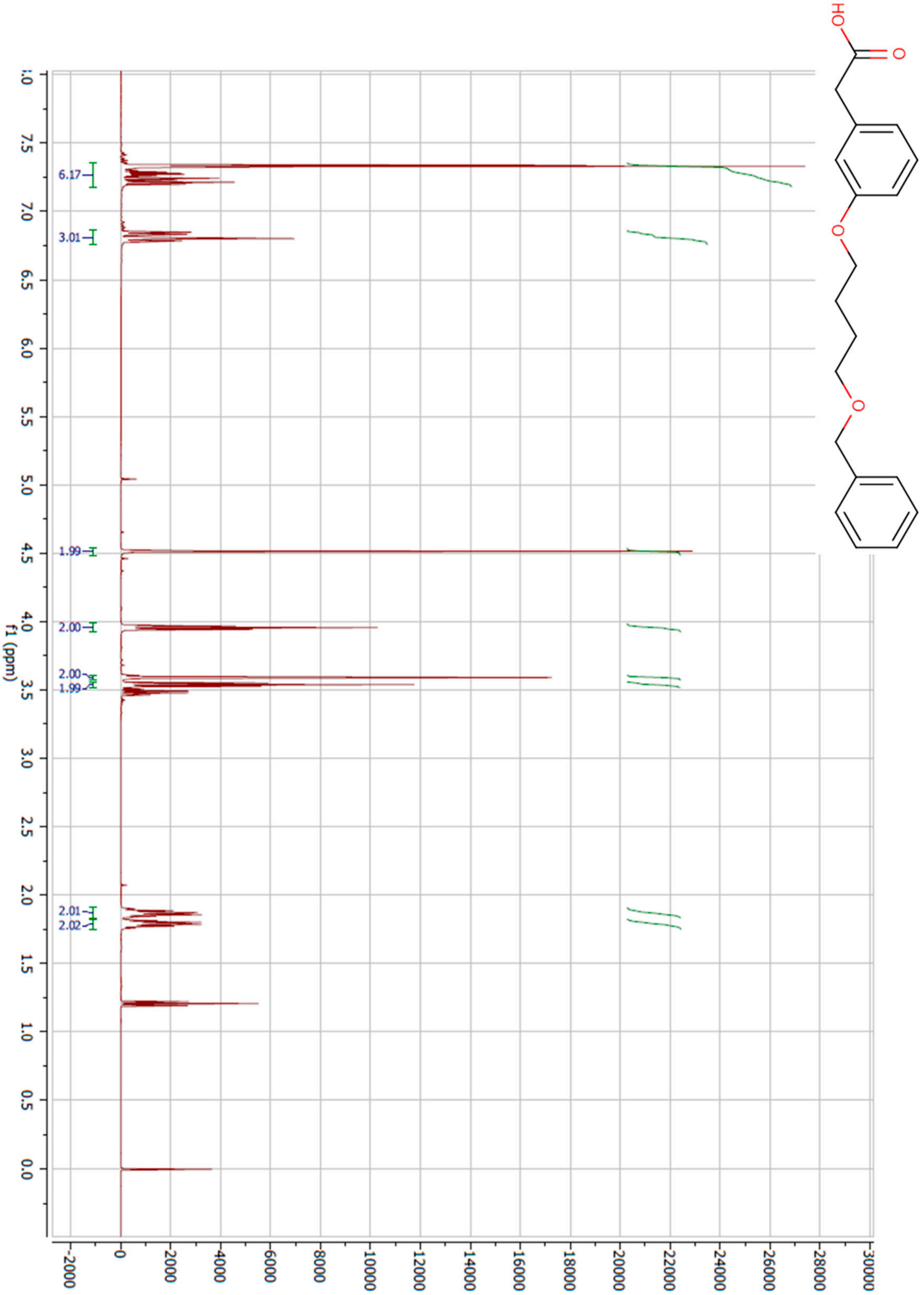

2-(3-{[4-(benzyloxy)butyl]oxy}phenyl)acetic acid (**9b**; ZHAW4560)

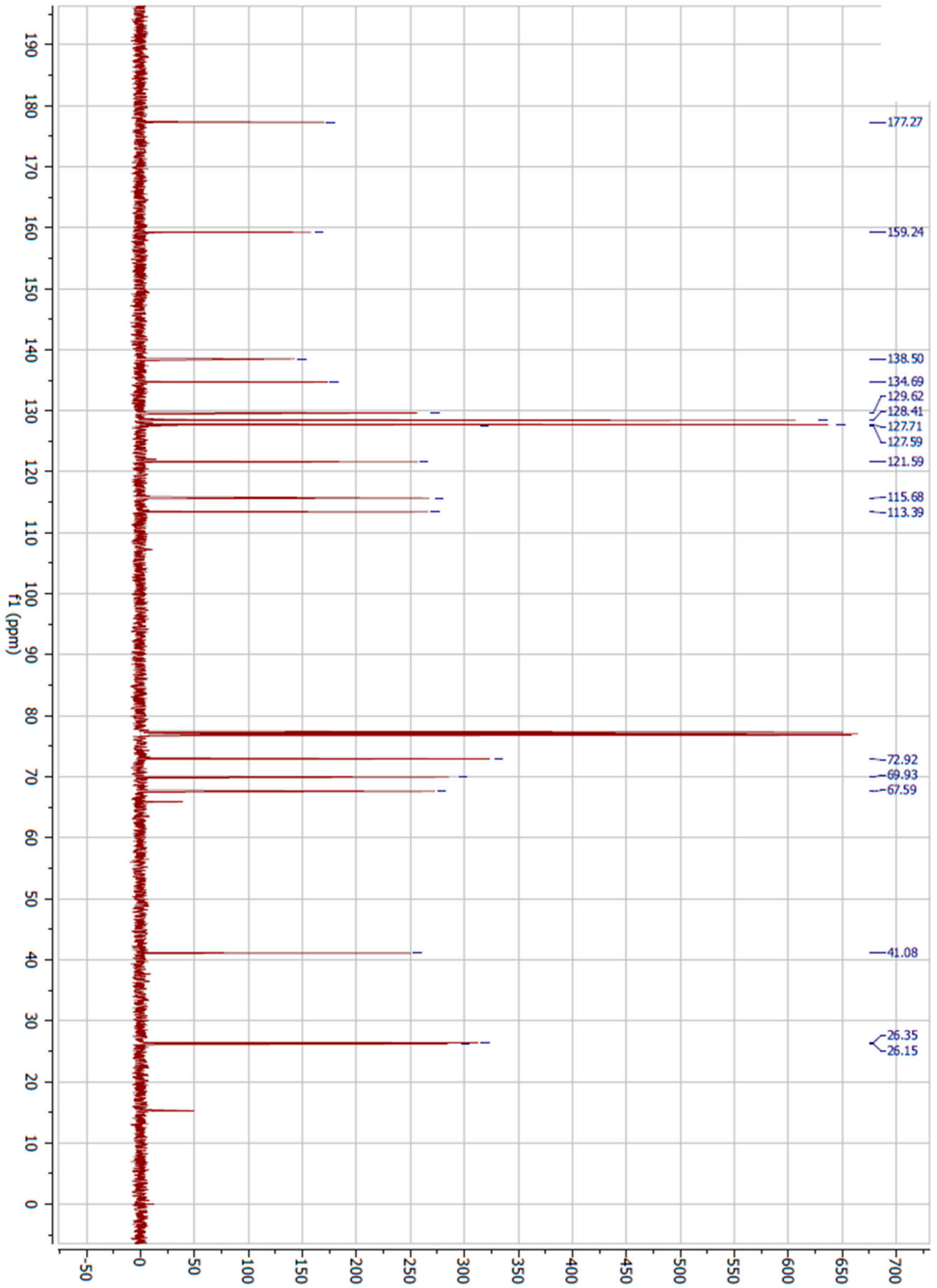

2-(3-{[5-(benzyloxy)pentyl]oxy}phenyl)acetic acid (**9c**; ZHAW4929)

NMR

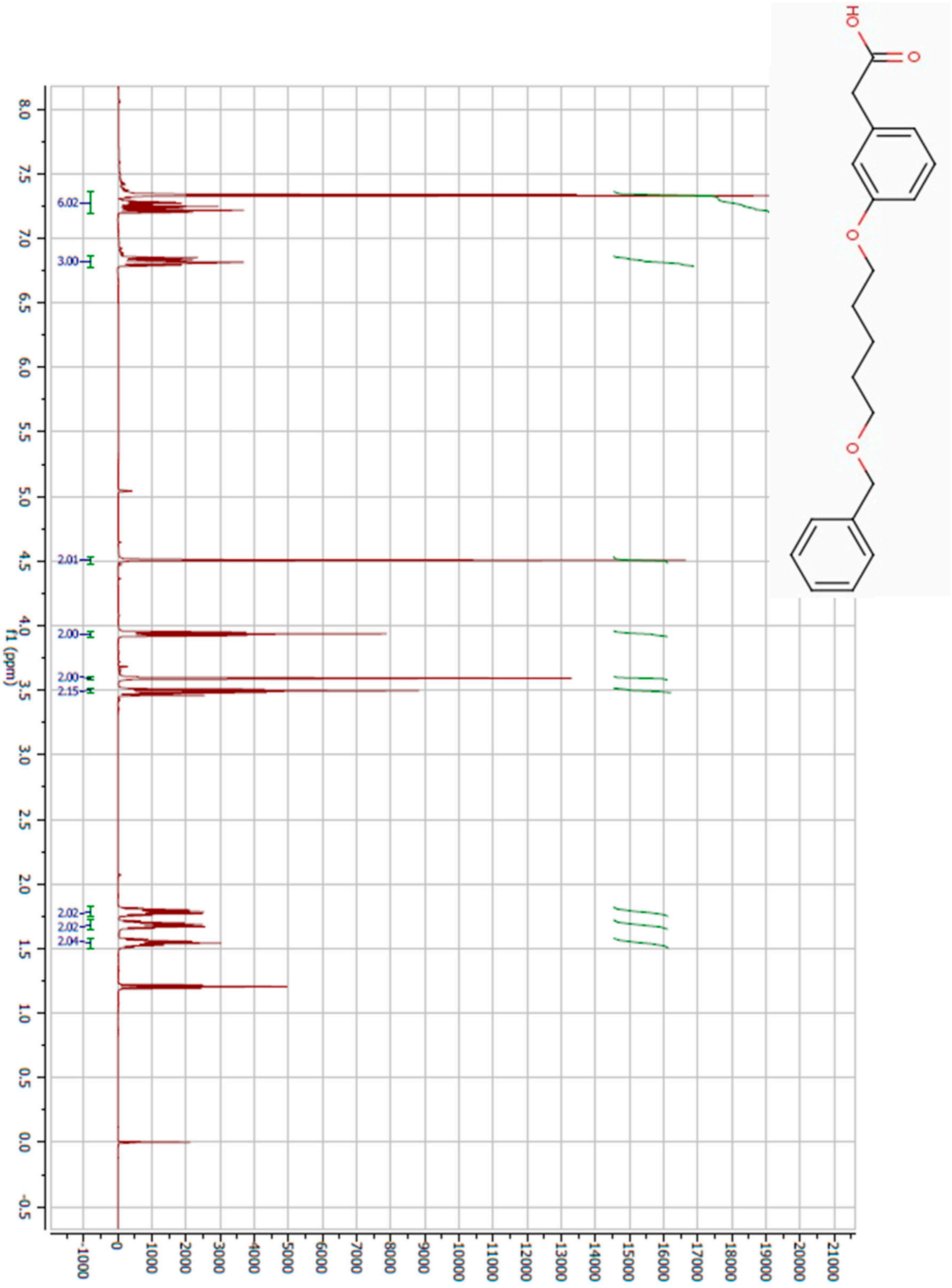

2-(3-{[5-(benzyloxy)pentyl]oxy}phenyl)acetic acid (**9c**; ZHAW4929)

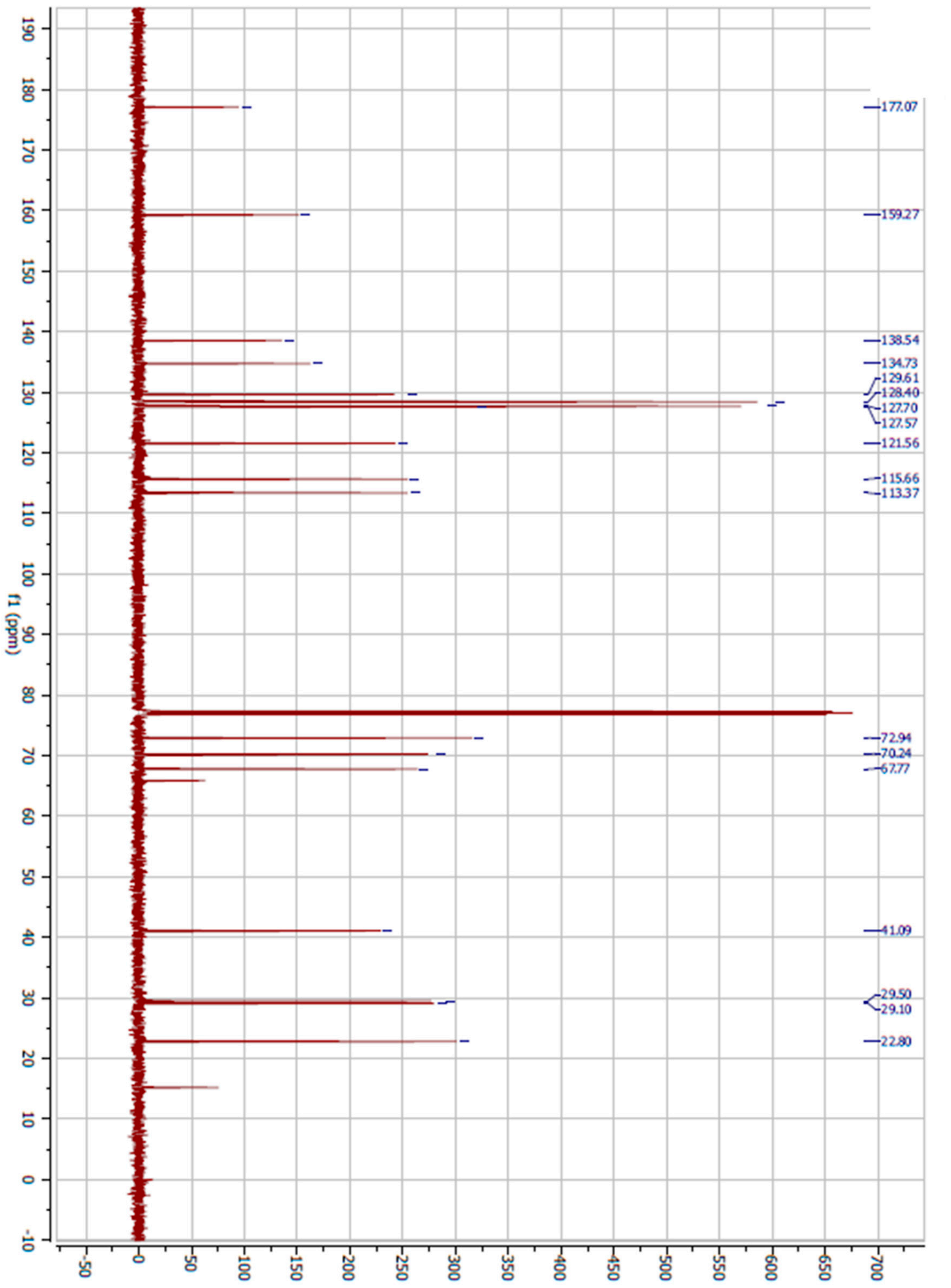

HRMS

2-(3-{[5-(benzyloxy)pentyl]oxy}phenyl)acetic acid (**9c**; ZHAW4929)

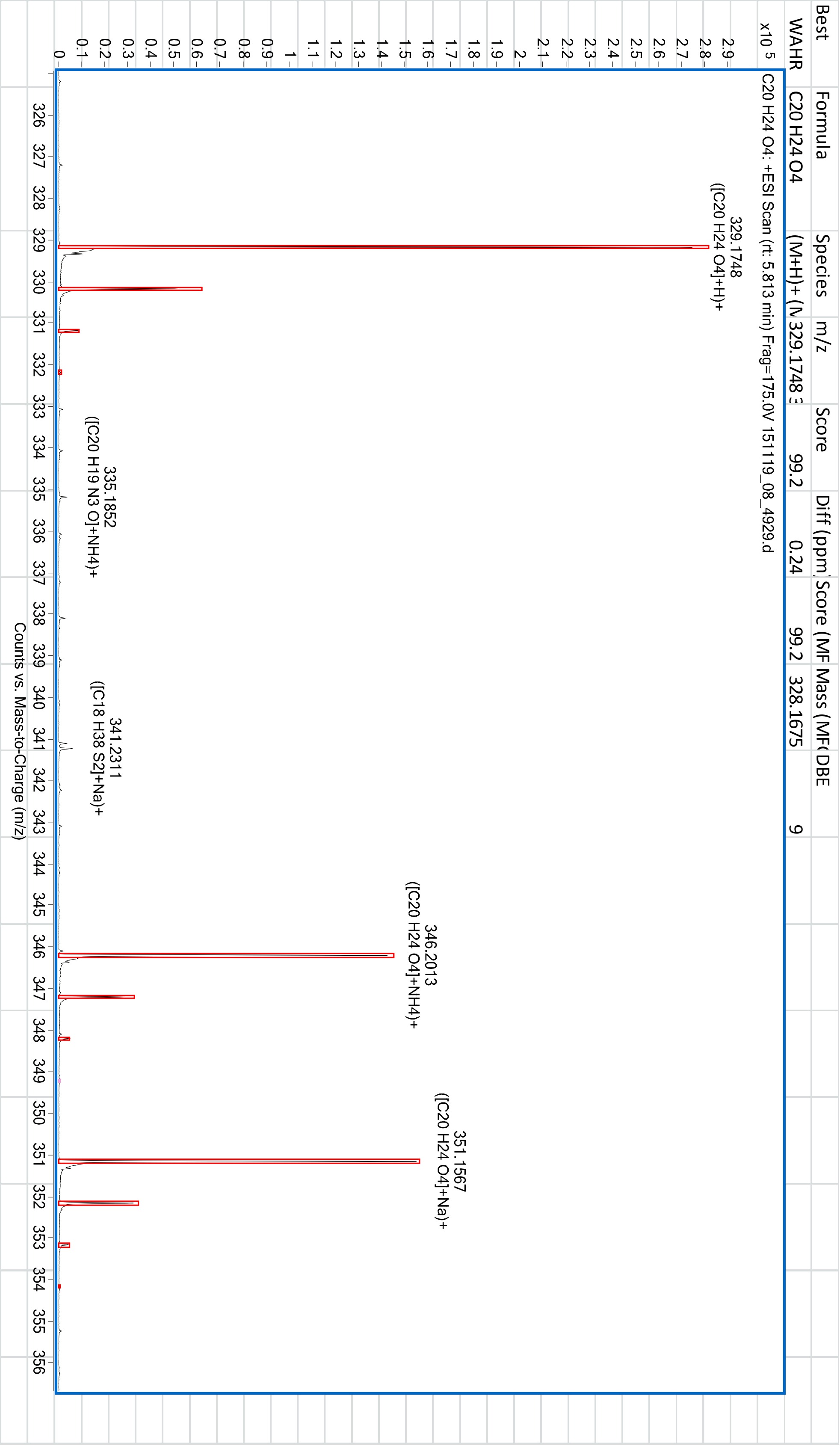

2-(3-{[6-(benzyloxy)hexyl]oxy}phenyl)acetic acid (**9d**; ZHAW4930)

<u>**NMR**</u>

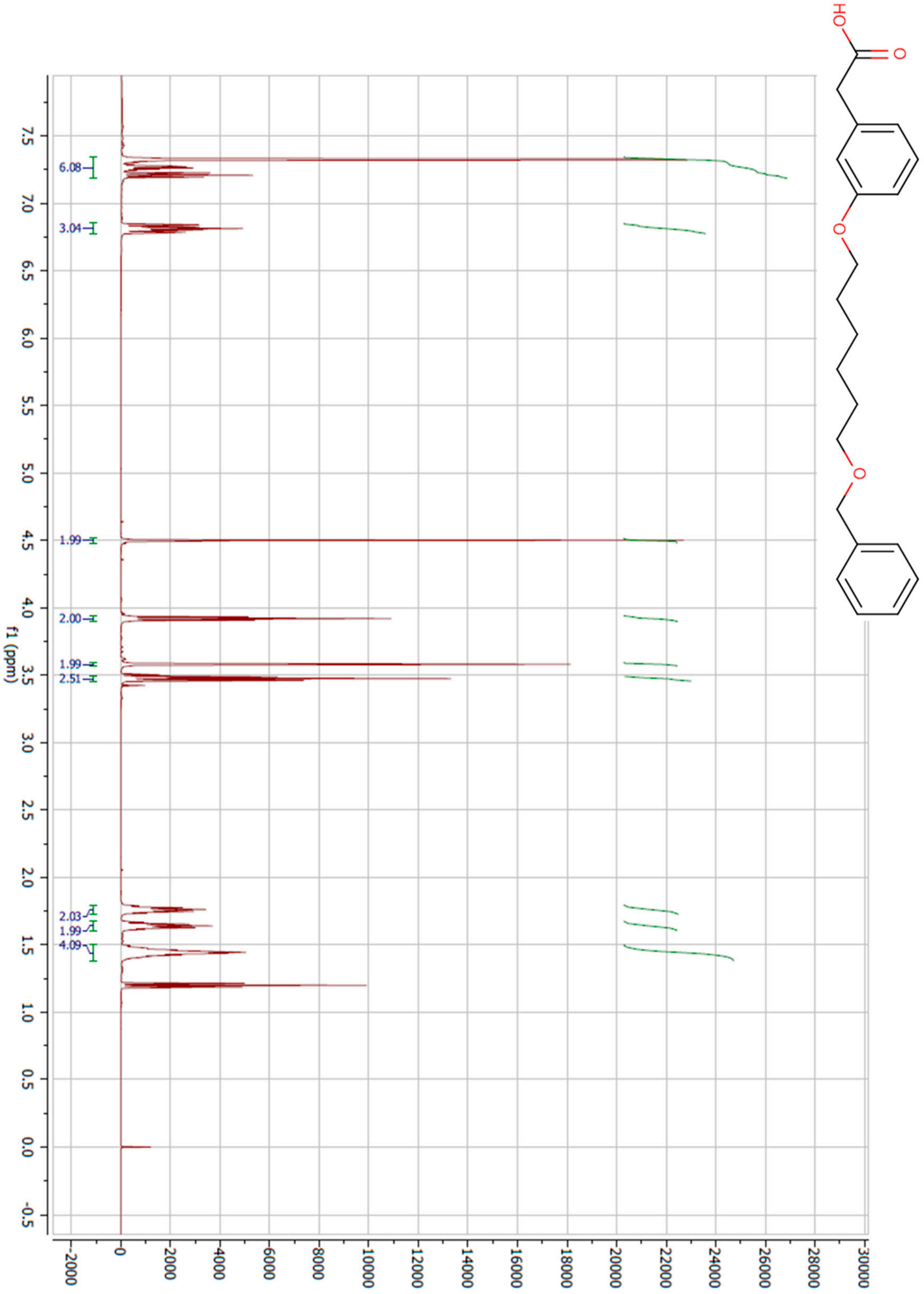

2-(3-{[6-(benzyloxy)hexyl]oxy}phenyl)acetic acid (**9d**; ZHAW4930)

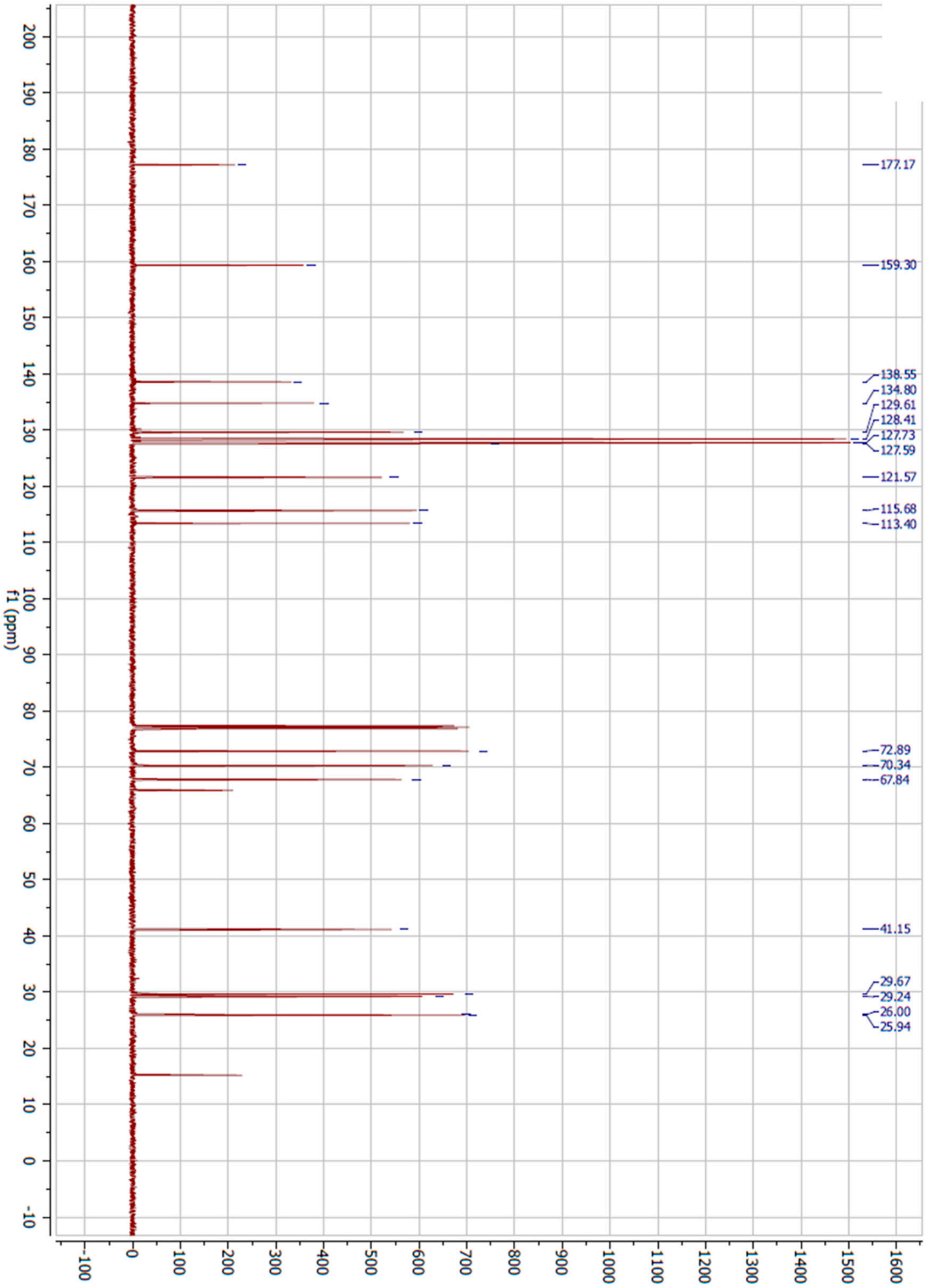

N-(2-benzyl-1,3-dioxo-2,3-dihydro-1H-isoindol-5-yl)-2-(3-{[3-(benzyloxy)propyl]oxy}phenyl)acetamide (**10a**; ZHAW4754)

NMR

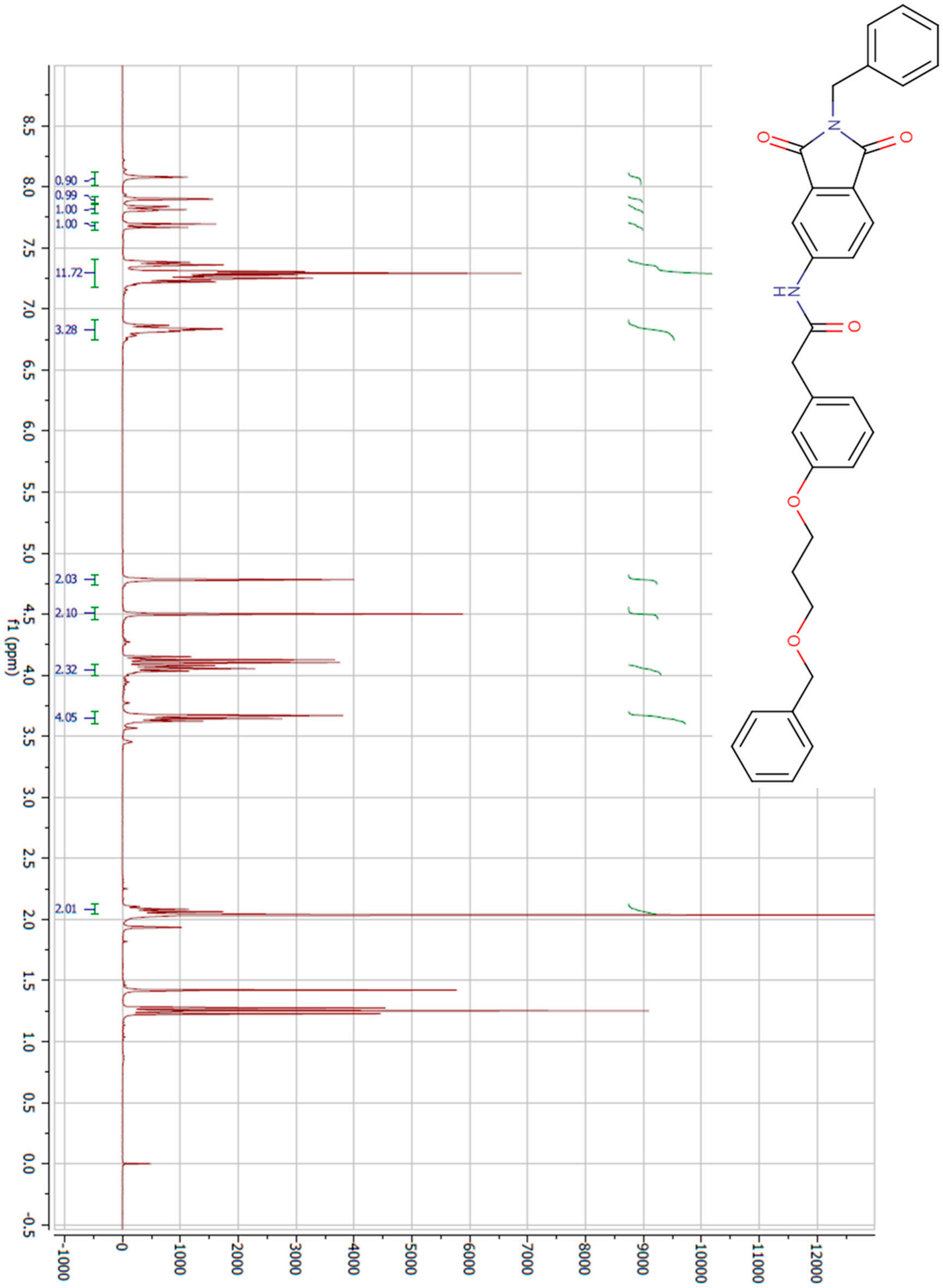

N-(2-benzyl-1,3-dioxo-2,3-dihydro-1H-isoindol-5-yl)-2-(3-{[3-(benzyloxy)propyl]oxy}phenyl)acetamide (**10a**; ZHAW4754)

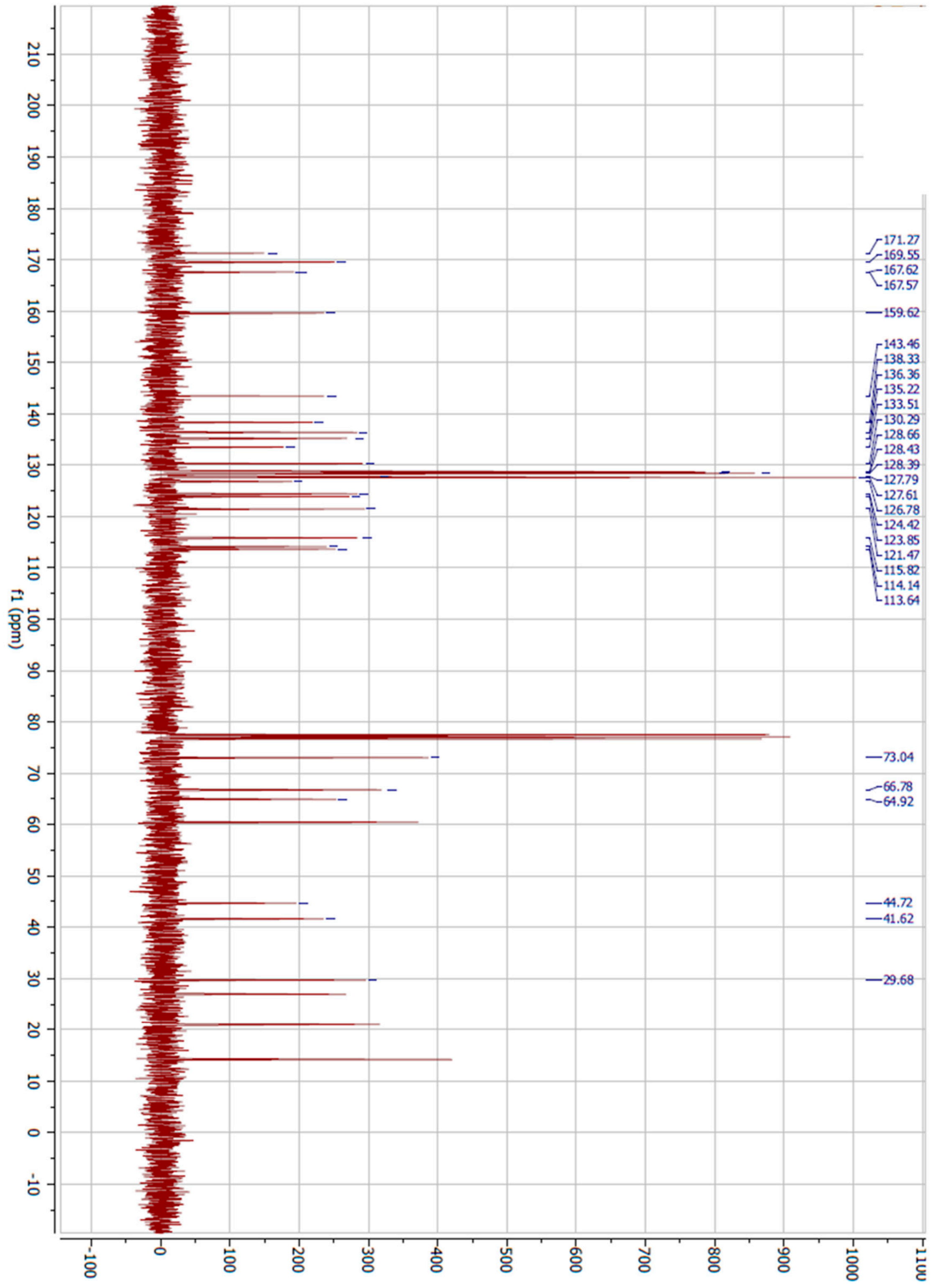

N-(2-benzyl-1,3-dioxo-2,3-dihydro-1H-isoindol-5-yl)-2-(3-{[4-(benzyloxy)butyl]oxy}phenyl)acetamide (**10b**; ZHAW4755)

NMR

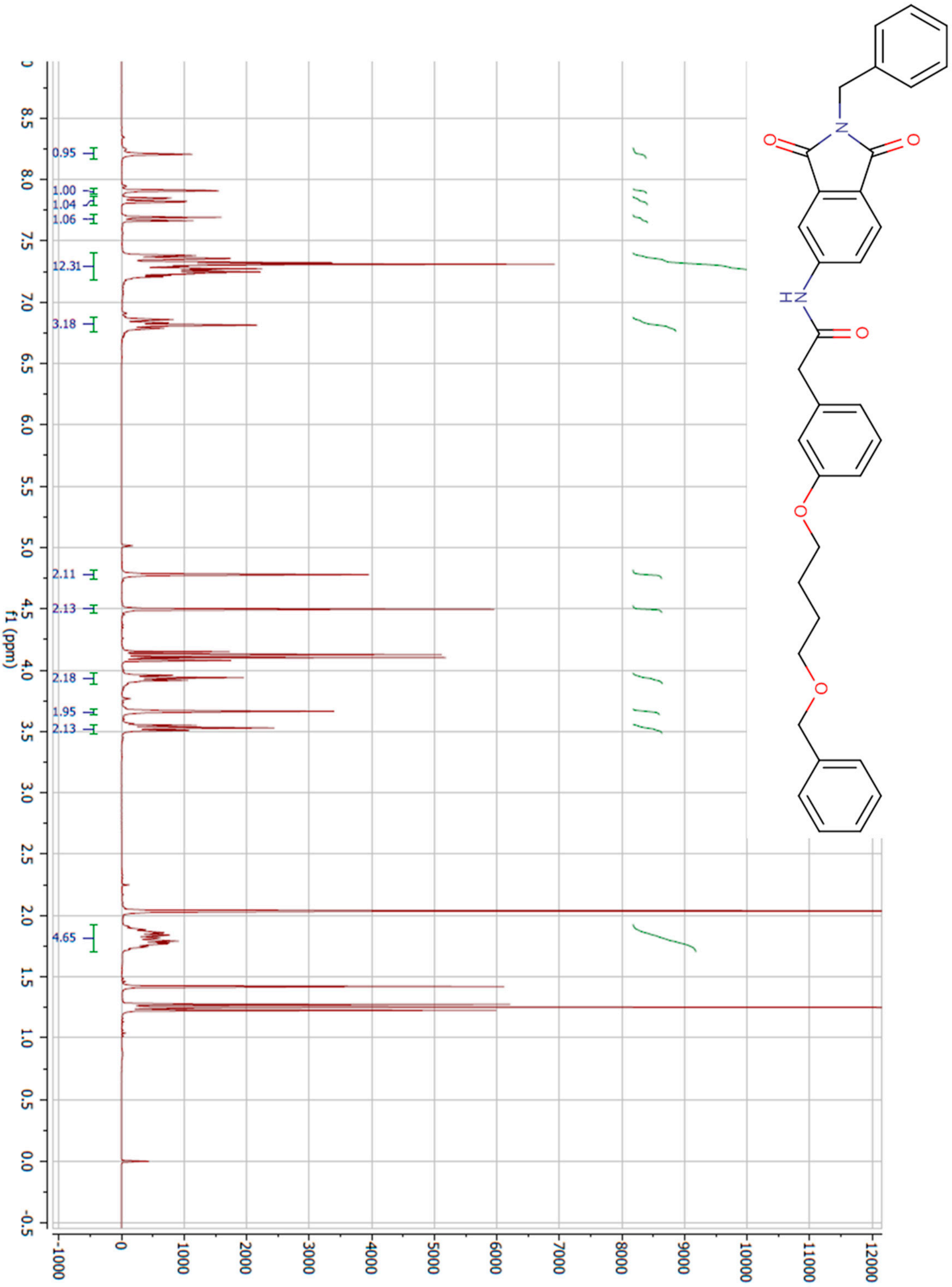

N-(2-benzyl-1,3-dioxo-2,3-dihydro-1H-isoindol-5-yl)-2-(3-{[4-(benzyloxy)butyl]oxy}phenyl)acetamide (**10b**; ZHAW4755)

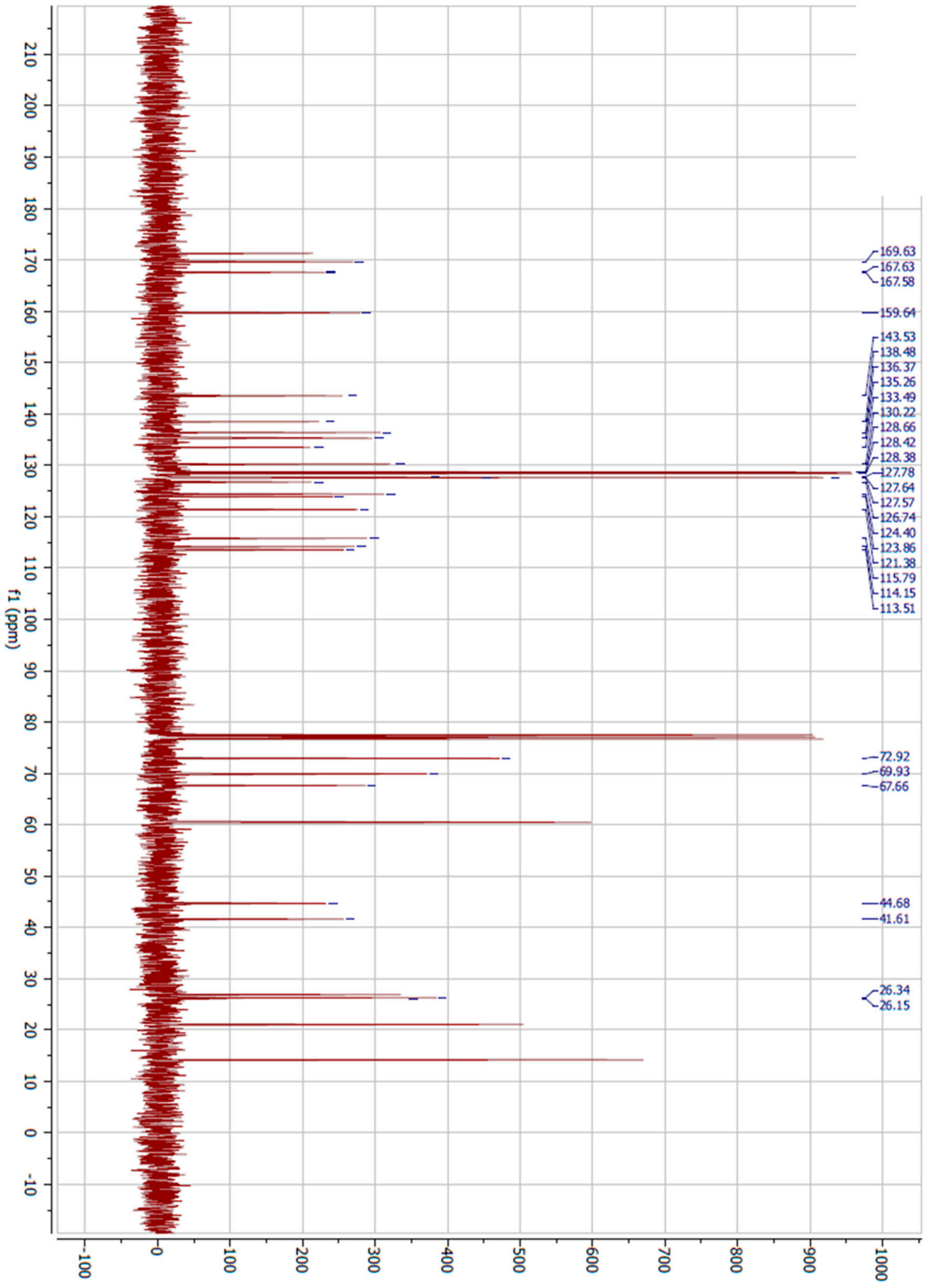

N-(2-benzyl-1,3-dioxo-2,3-dihydro-1H-isoindol-5-yl)-2-(3-{[5-(benzyloxy)pentyl]oxy}phenyl)acetamide (**10c**; ZHAW5606)

NMR

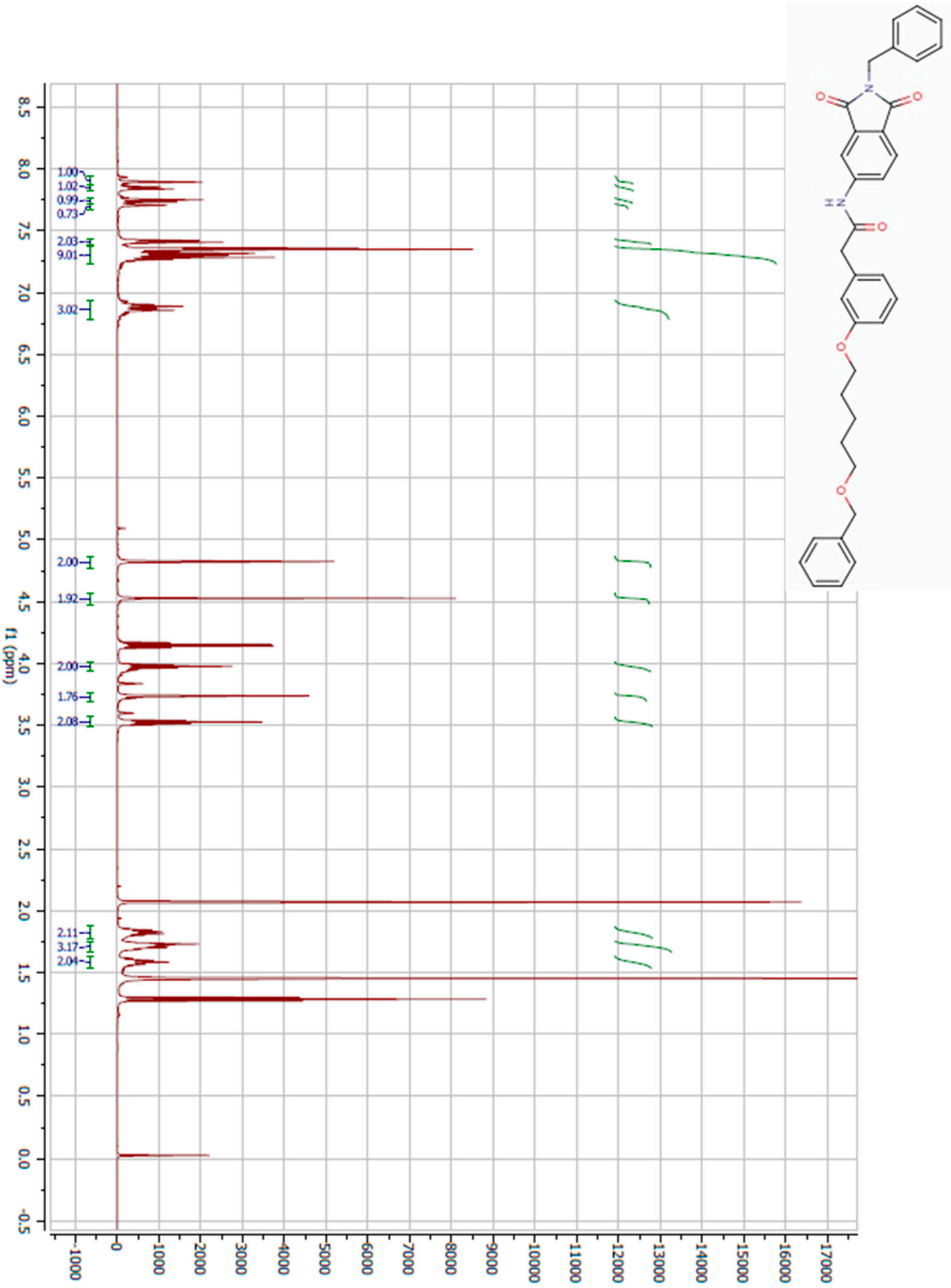

N-(2-benzyl-1,3-dioxo-2,3-dihydro-1H-isoindol-5-yl)-2-(3-{[5-(benzyloxy)pentyl]oxy}phenyl)acetamide (**10c**; ZHAW5606)

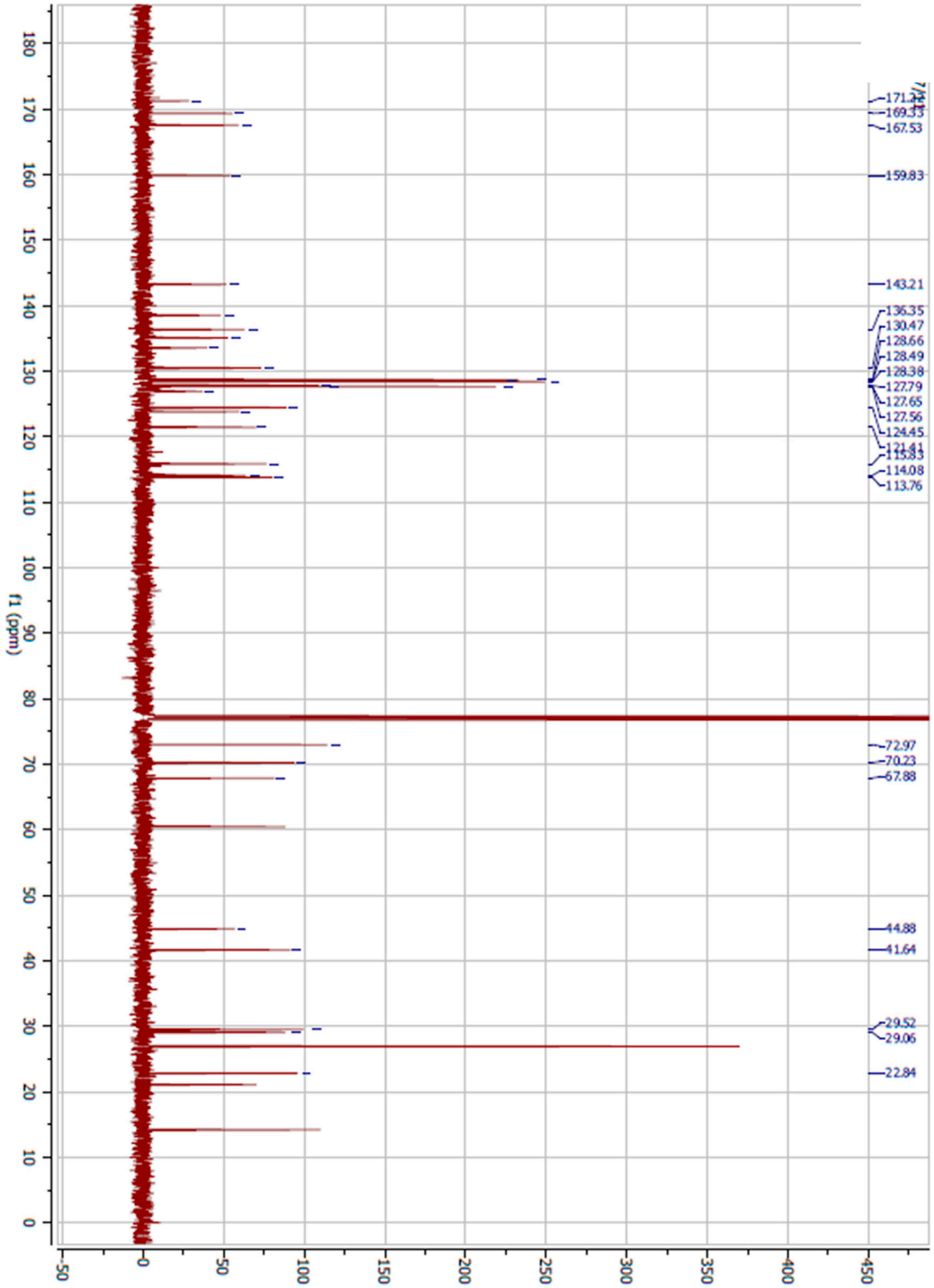

HRMS

N-(2-benzyl-1,3-dioxo-2,3-dihydro-1H-isoindol-5-yl)-2-(3-{[5-(benzyloxy)pentyl]oxy}phenyl)acetamide (**10c**; ZHAW5606)

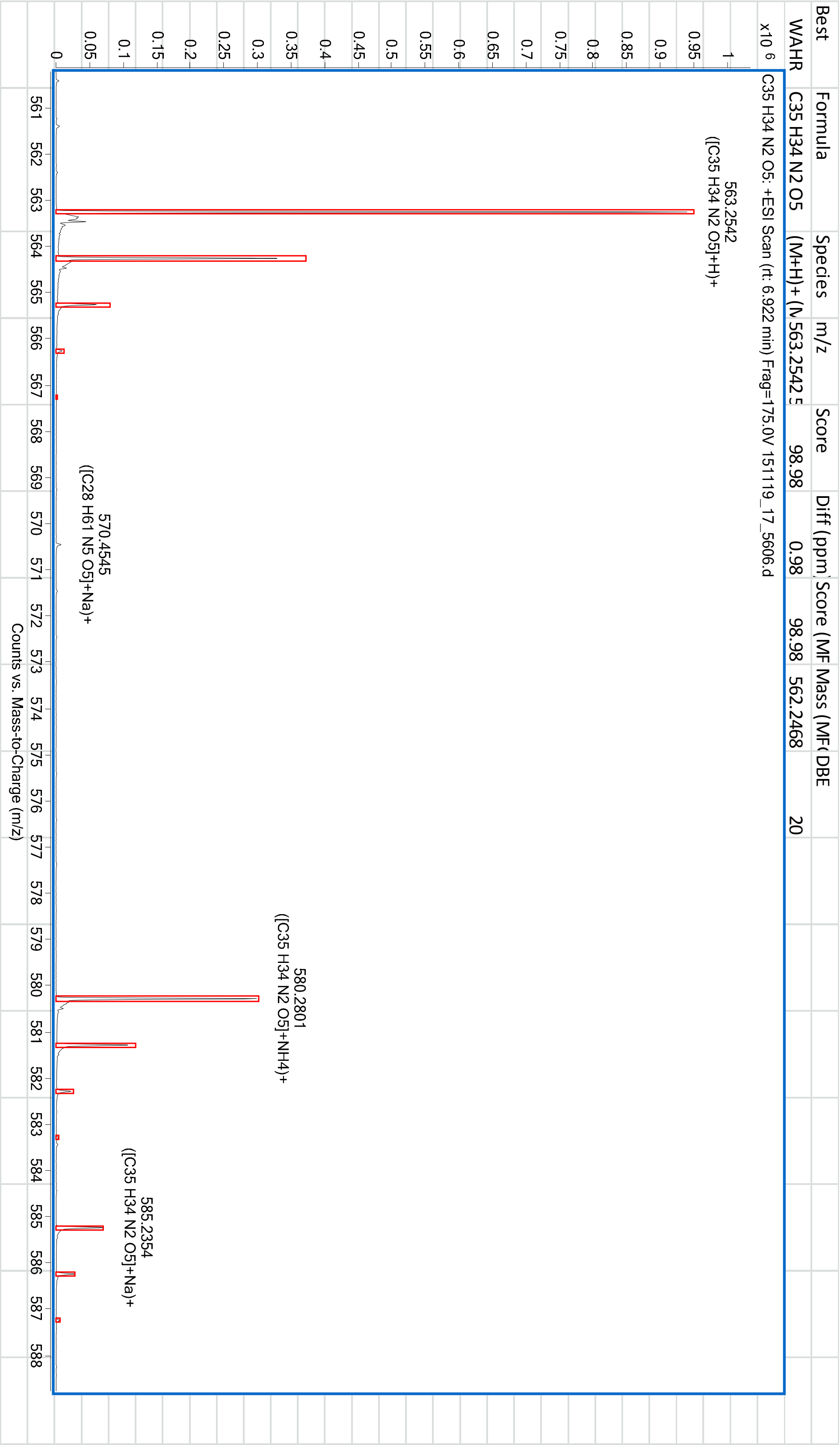

N-(2-benzyl-1,3-dioxo-2,3-dihydro-1H-isoindol-5-yl)-2-[3-(3-hydroxypropoxy)phenyl]acetamide (**11**; ZHAW4756)

NMR

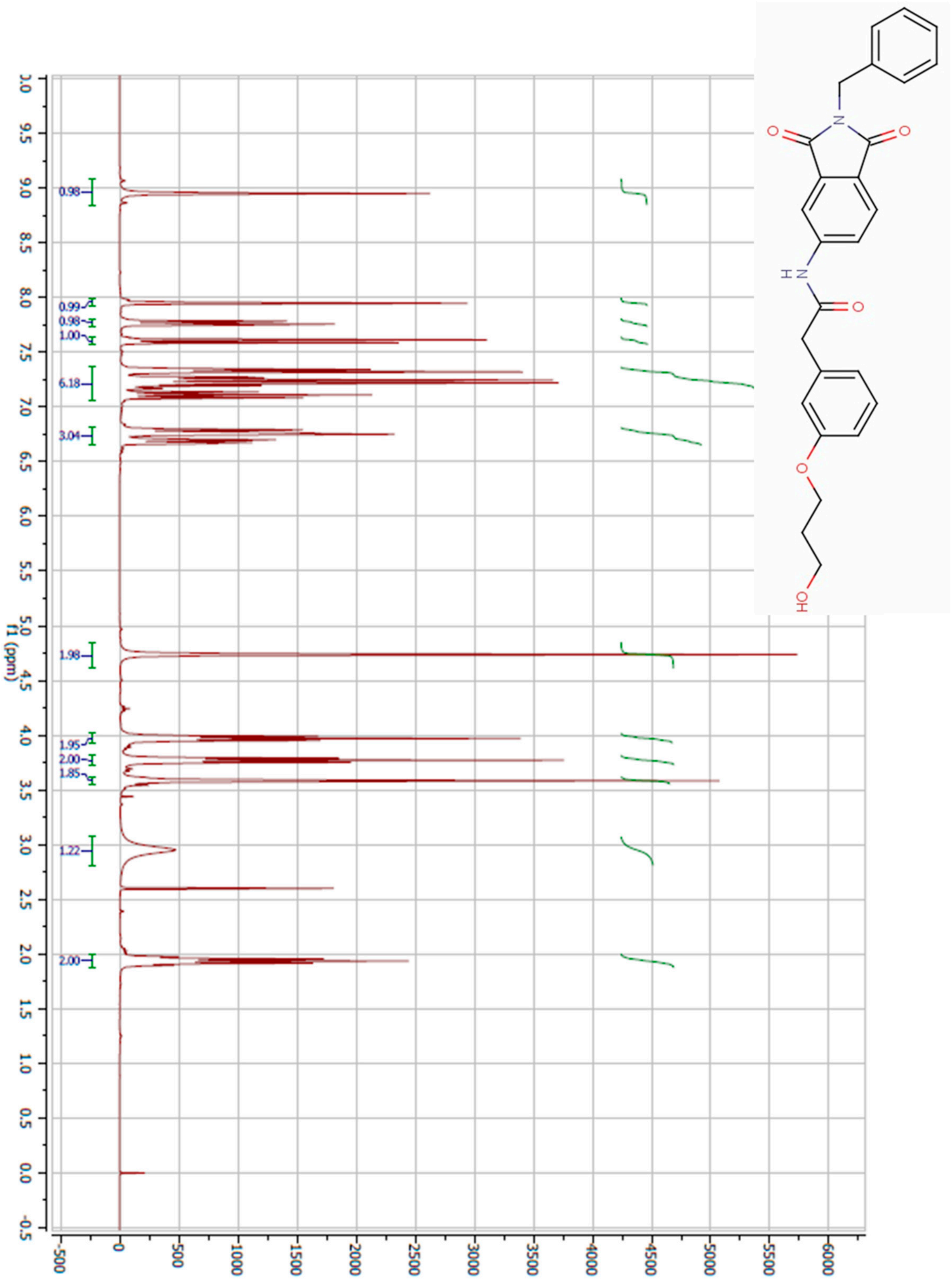

N-(2-benzyl-1,3-dioxo-2,3-dihydro-1H-isoindol-5-yl)-2-[3-(3-hydroxypropoxy)phenyl]acetamide (**11**; ZHAW4756)

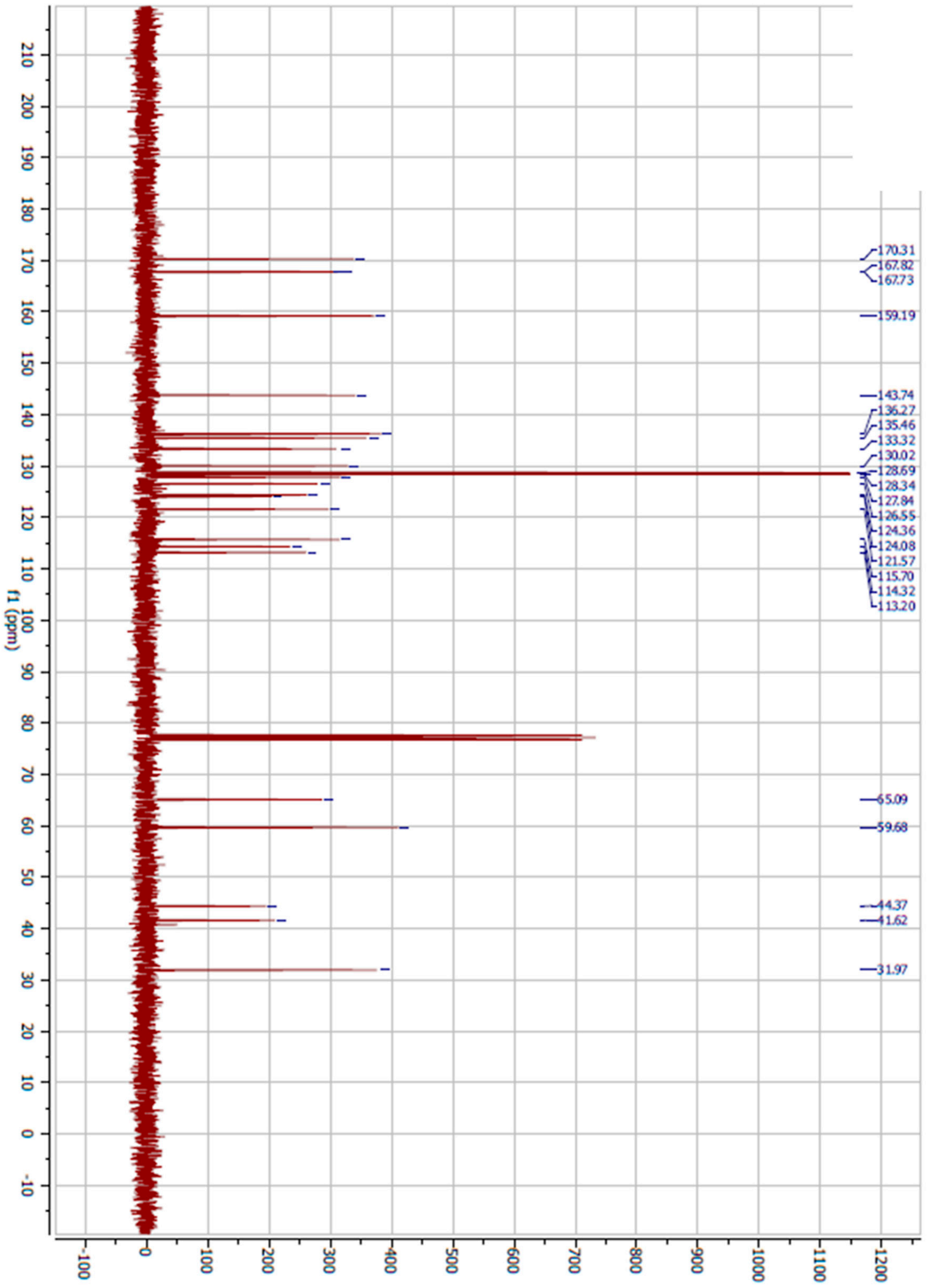

HRMS

N-(2-benzyl-1,3-dioxo-2,3-dihydro-1H-isoindol-5-yl)-2-[3-(3-hydroxypropoxy)phenyl]acetamide (**11**; ZHAW4756)

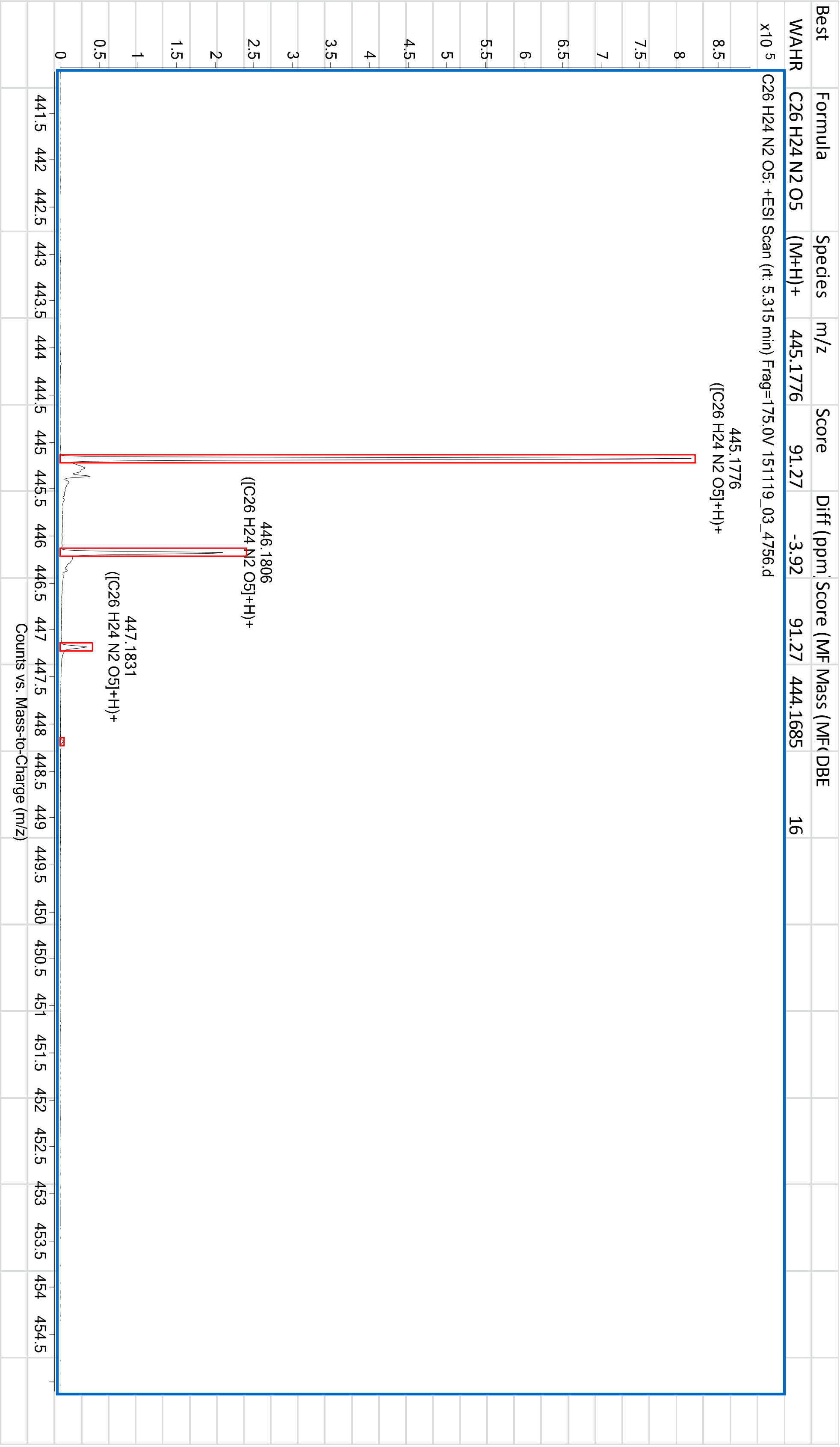

IC$_{50}$

N-(2-benzyl-1,3-dioxo-2,3-dihydro-1H-isoindol-5-yl)-2-[3-(3-hydroxypropoxy)phenyl]acetamide (**11**; ZHAW4756)

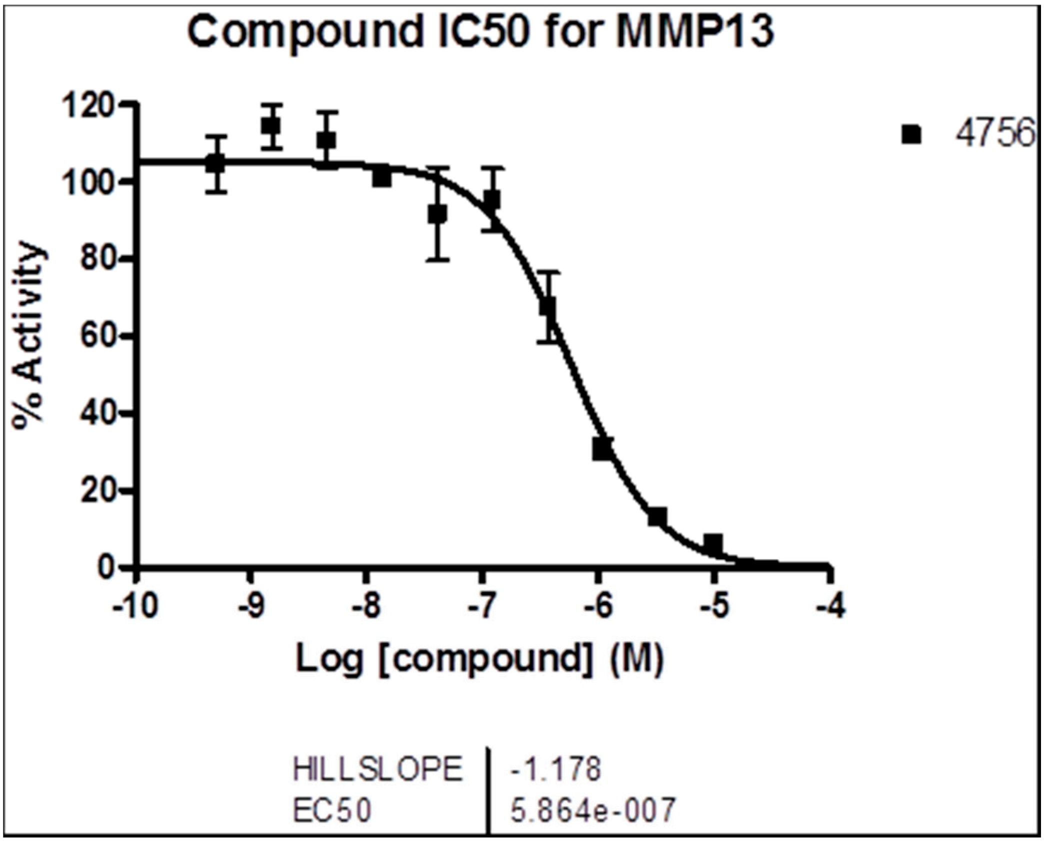

N-(2-benzyl-1,3-dioxo-2,3-dihydro-1H-isoindol-5-yl)-2-[3-(4-hydroxybutoxy)phenyl]acetamide
(**12**; ZHAW4757)

NMR

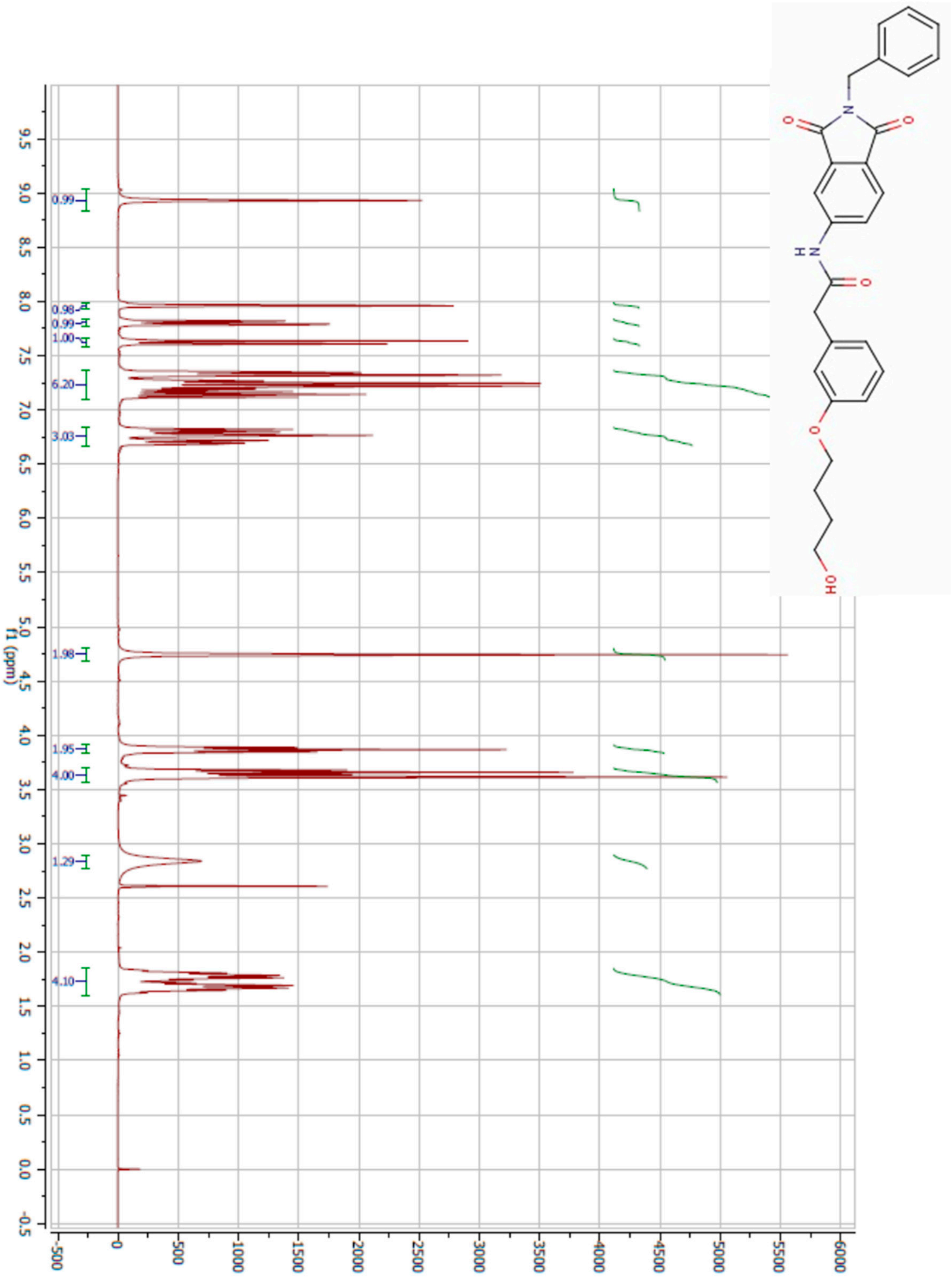

N-(2-benzyl-1,3-dioxo-2,3-dihydro-1H-isoindol-5-yl)-2-[3-(4-hydroxybutoxy)phenyl]acetamide (**12**; ZHAW4757)

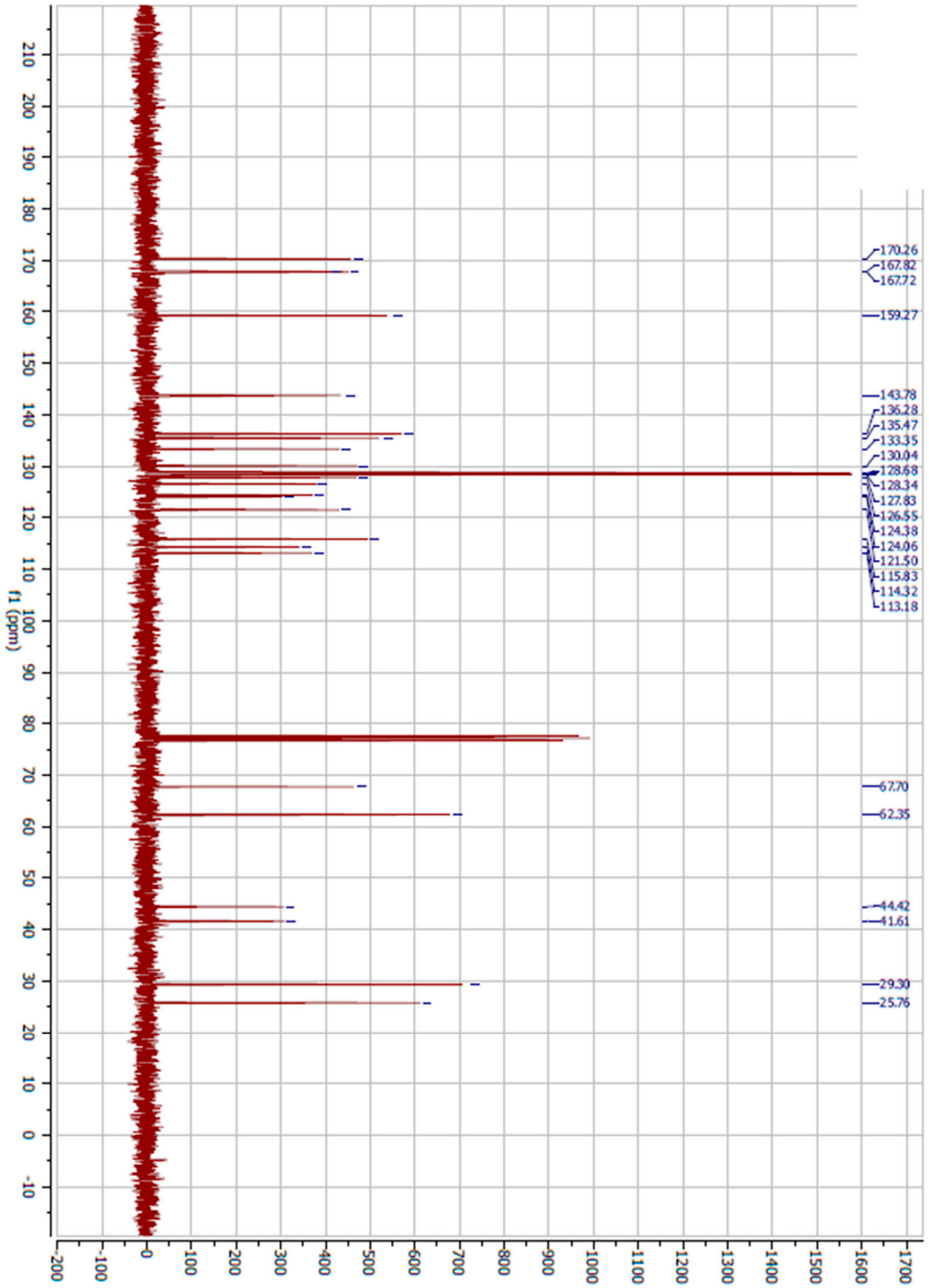

HRMS

N-(2-benzyl-1,3-dioxo-2,3-dihydro-1H-isoindol-5-yl)-2-[3-(4-hydroxybutoxy)phenyl]acetamide (**12**; ZHAW4757)

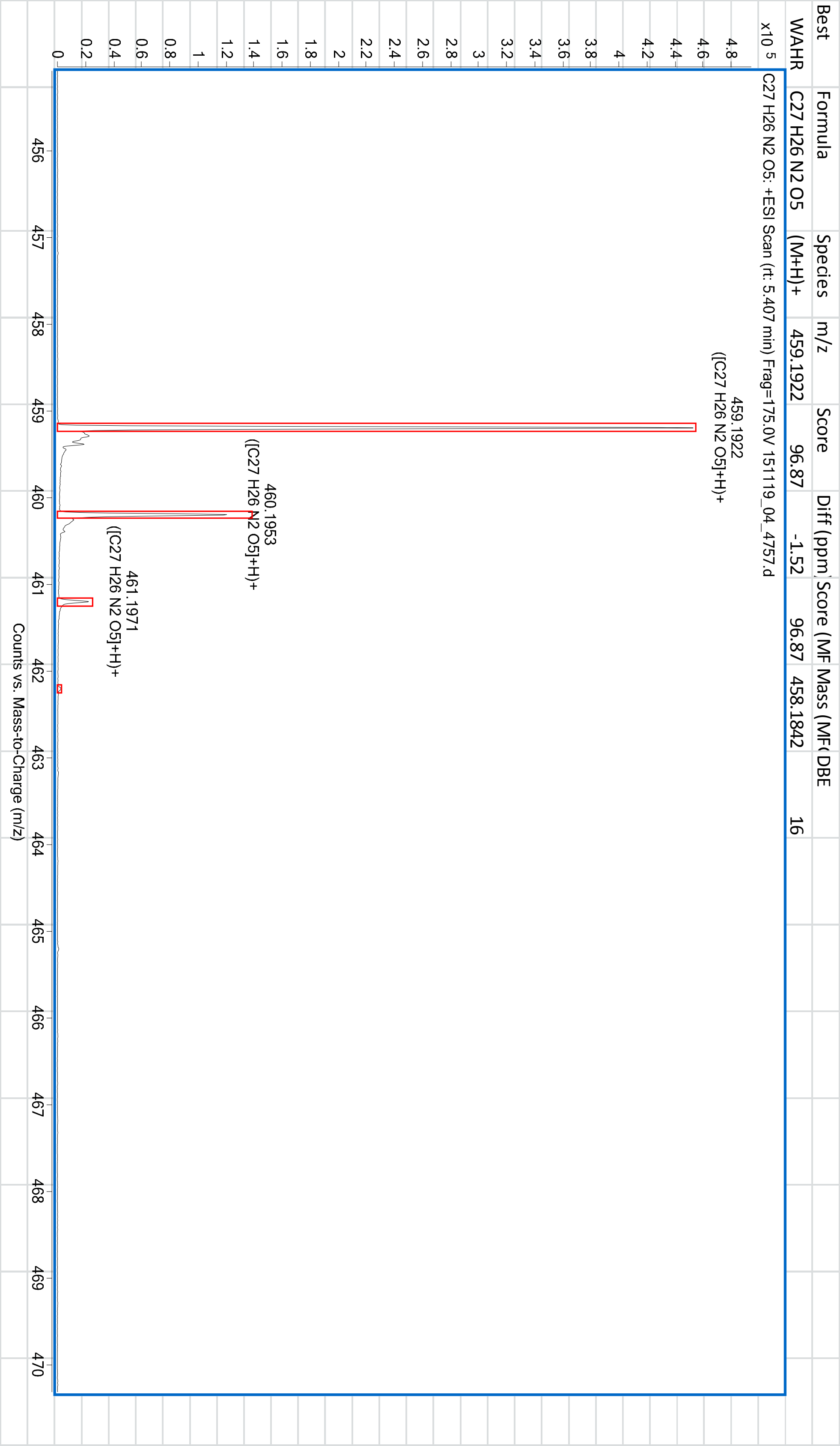

Best	Formula	Species	m/z	Score	Diff (ppm)	Score (MF	Mass (MF(	DBE
WAHR	C27 H26 N2 O5	(M+H)+	459.1922	96.87	-1.52	96.87	458.1842	16

IC50

N-(2-benzyl-1,3-dioxo-2,3-dihydro-1H-isoindol-5-yl)-2-[3-(4-hydroxybutoxy)phenyl]acetamide (**12**; ZHAW4757)

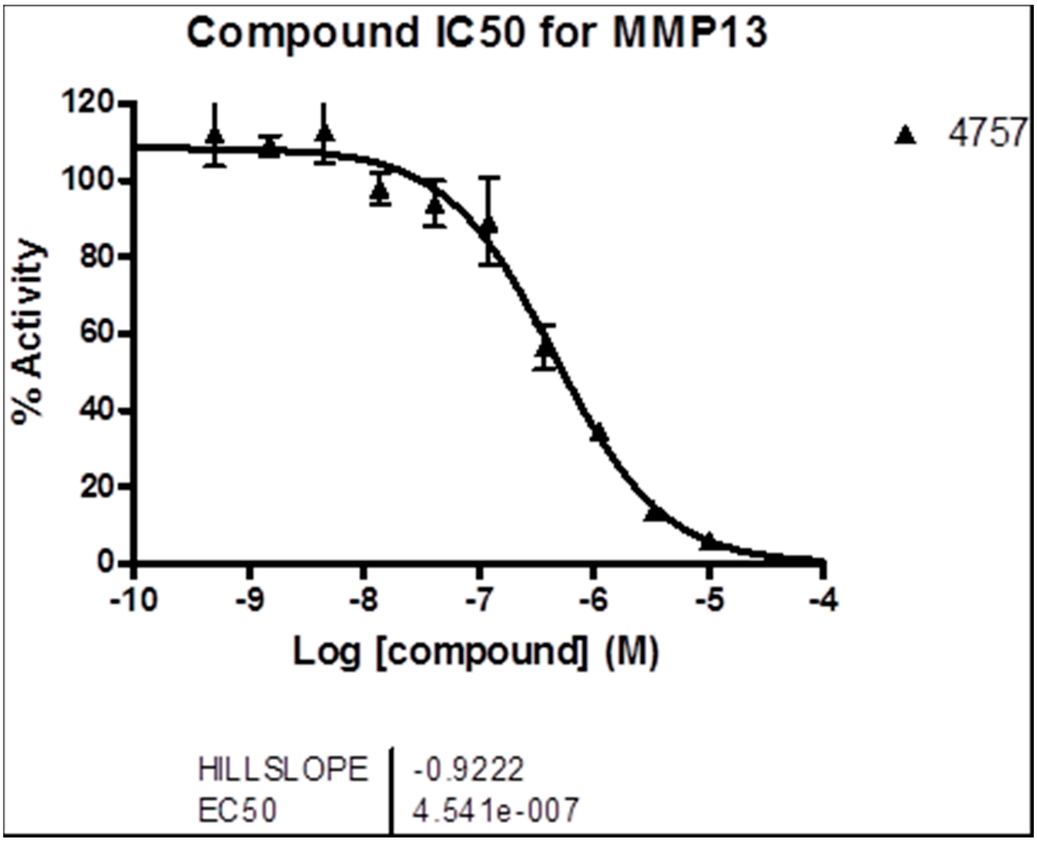

N-(2-benzyl-1,3-dioxo-2,3-dihydro-1H-isoindol-5-yl)-2-{3-[(6-hydroxyhexyl)oxy]phenyl}acetamide
(**13**; ZHAW5042)

NMR

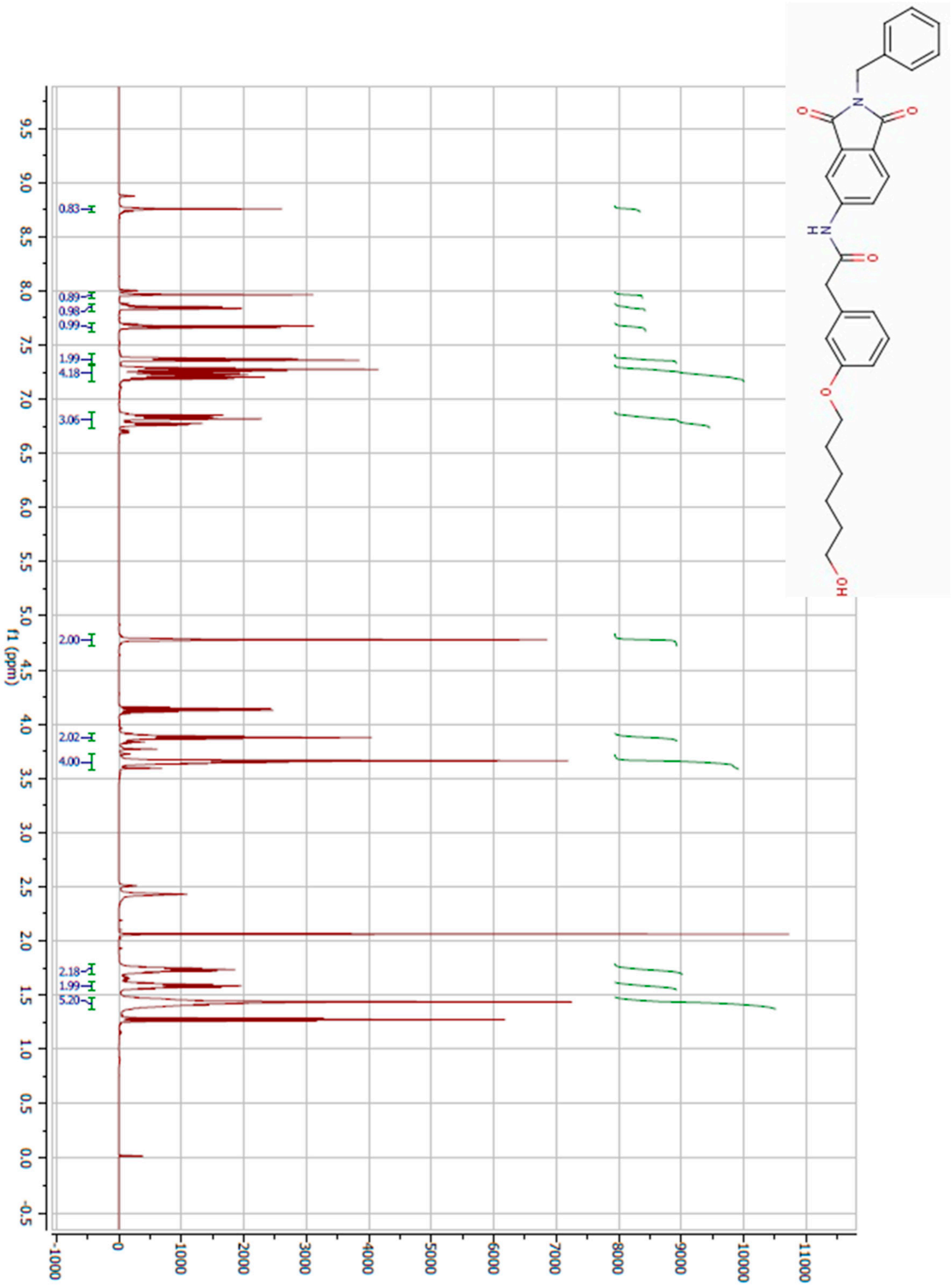

N-(2-benzyl-1,3-dioxo-2,3-dihydro-1H-isoindol-5-yl)-2-{3-[(6-hydroxyhexyl)oxy]phenyl}acetamide (**13**; ZHAW5042)

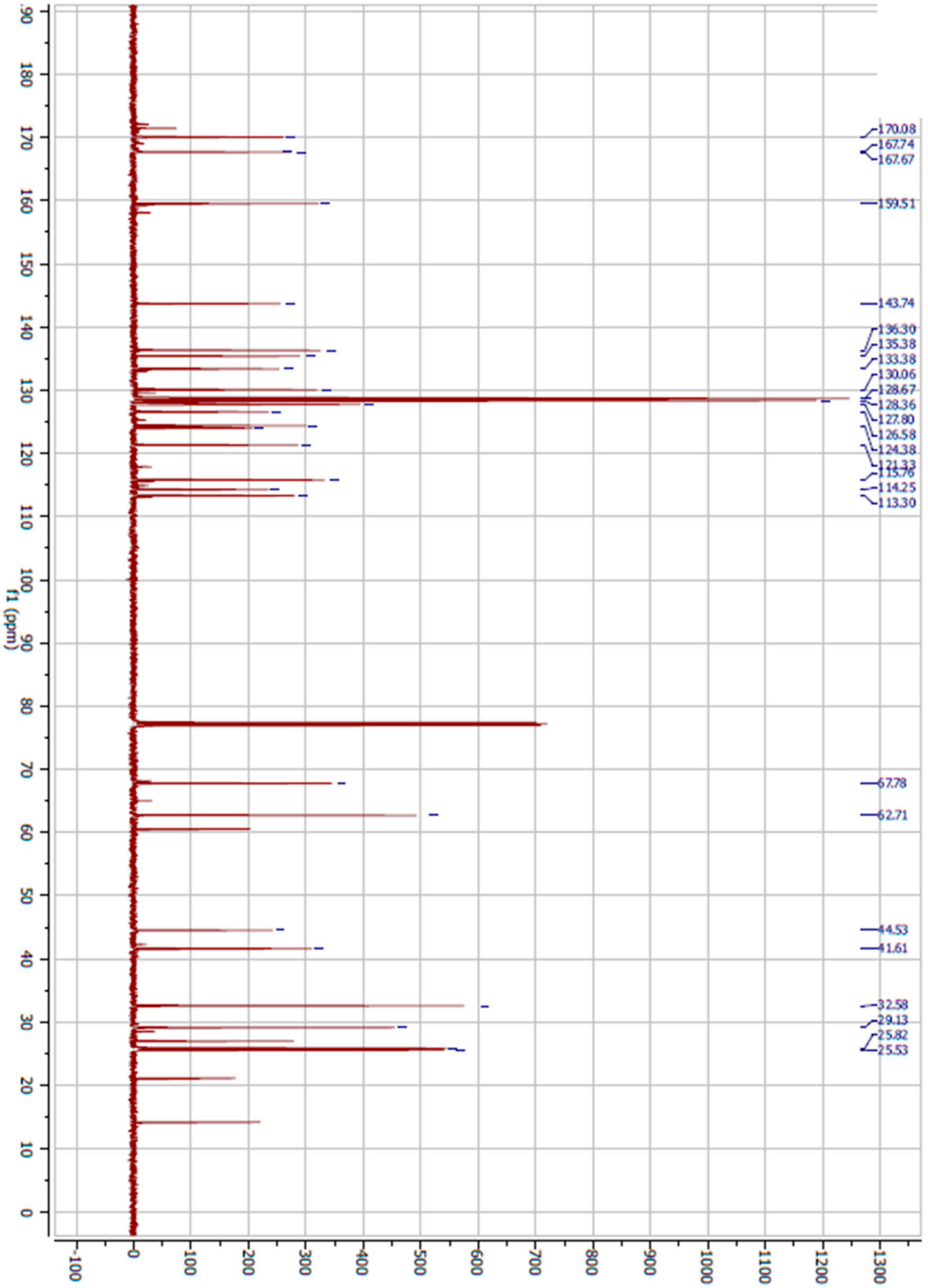

HRMS

N-(2-benzyl-1,3-dioxo-2,3-dihydro-1H-isoindol-5-yl)-2-{3-[(6-hydroxyhexyl)oxy]phenyl}acetamide (**13**; ZHAW5042)

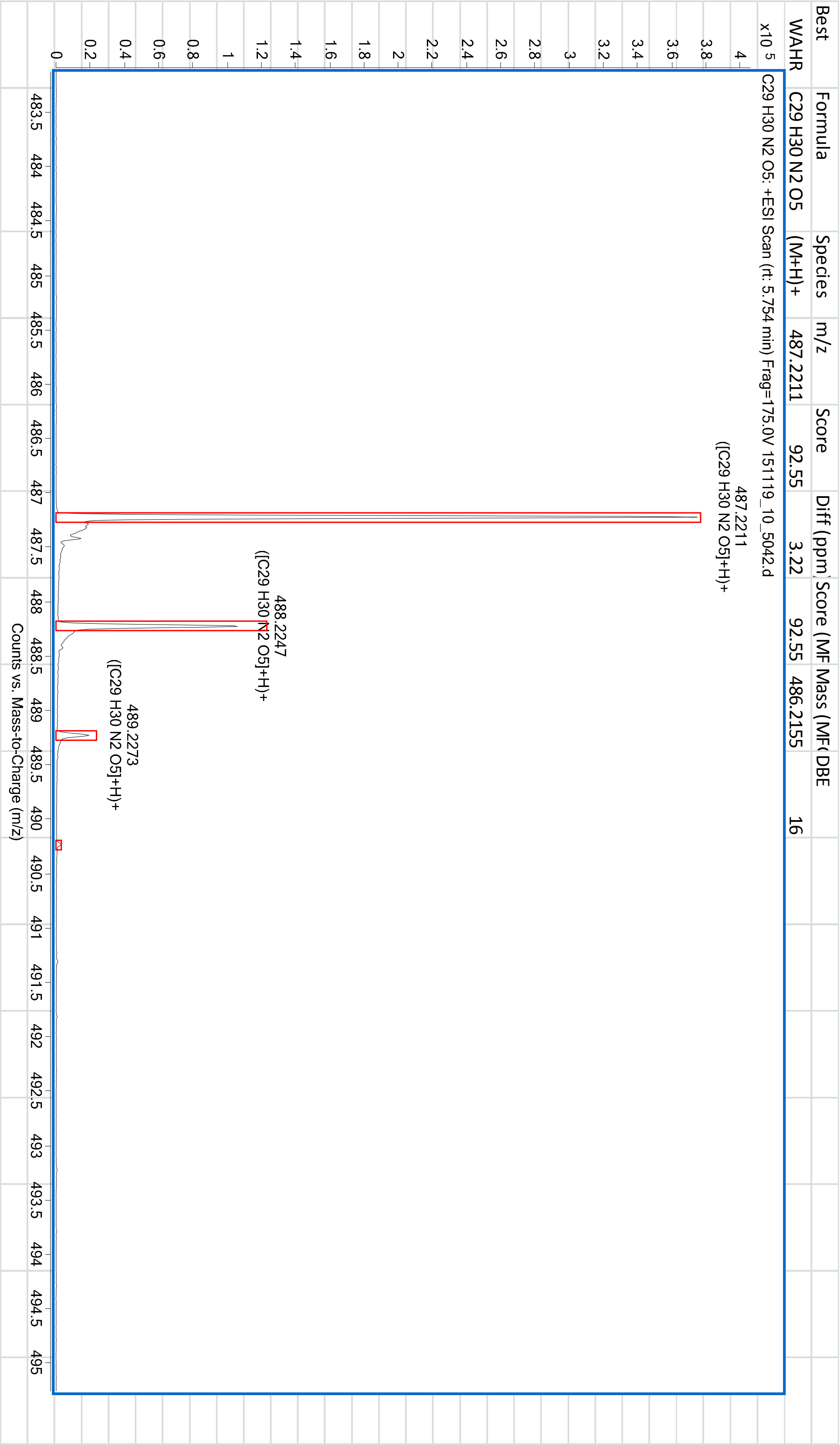

3-(3-{[(2-benzyl-1,3-dioxo-2,3-dihydro-1H-isoindol-5-yl)carbamoyl]methyl}phenoxy)propanoic acid (**14**; ZHAW4767)

NMR

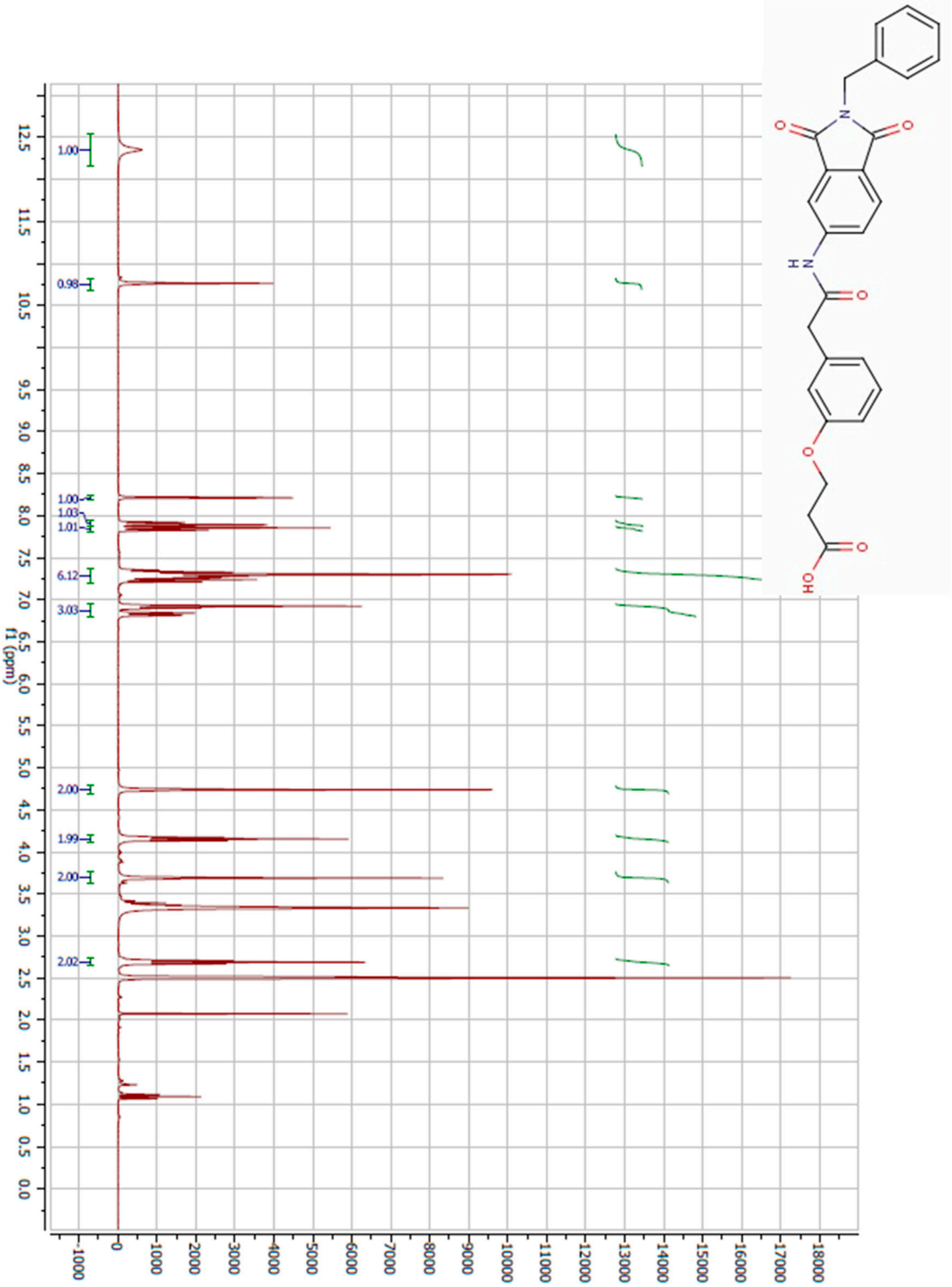

3-(3-{[(2-benzyl-1,3-dioxo-2,3-dihydro-1H-isoindol-5-yl)carbamoyl]methyl}phenoxy)propanoic acid (**14**; ZHAW4767)

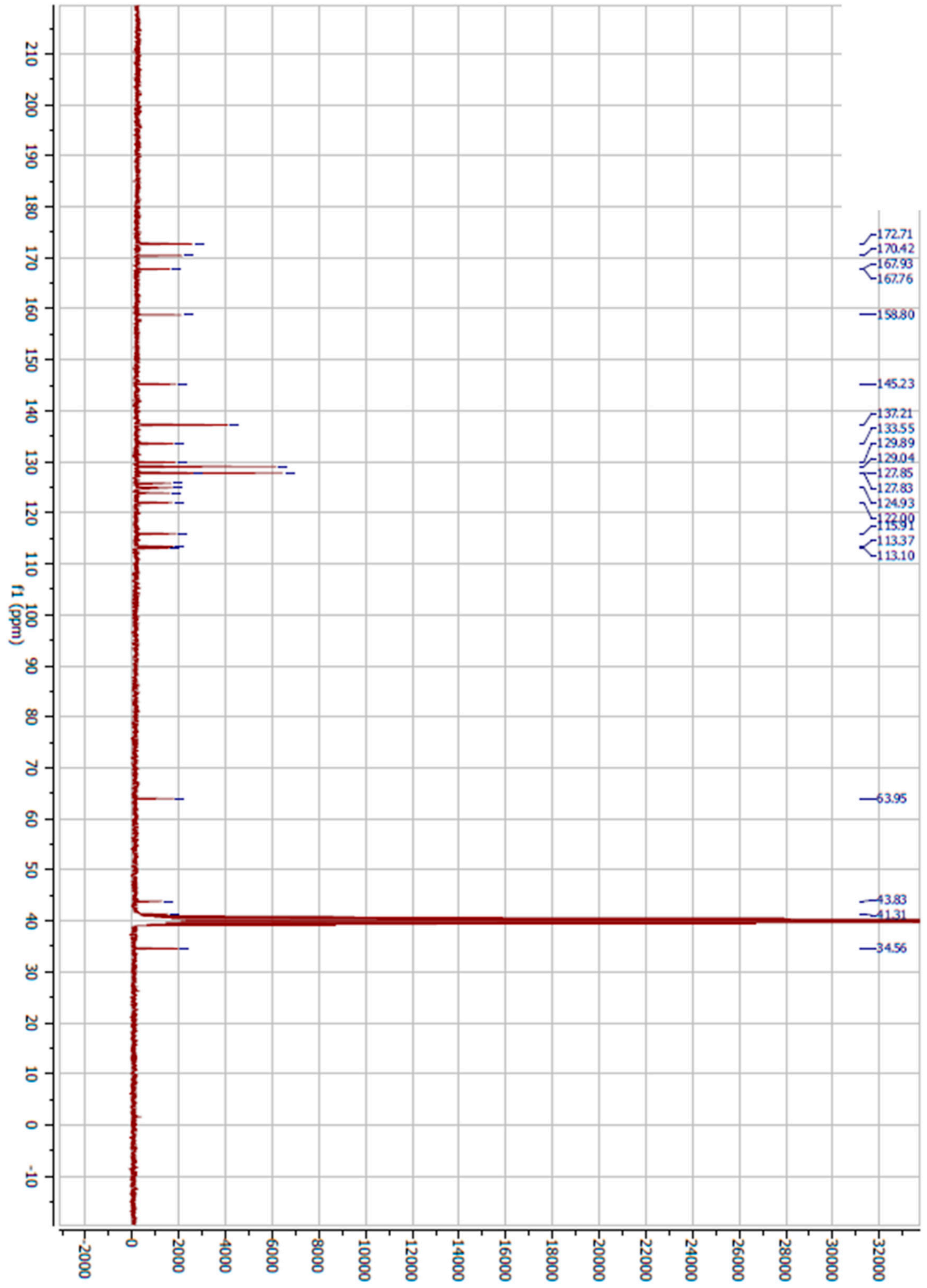

HRMS

3-(3-{[(2-benzyl-1,3-dioxo-2,3-dihydro-1H-isoindol-5-yl)carbamoyl]methyl}phenoxy)propanoic acid (**14**; ZHAW4767)

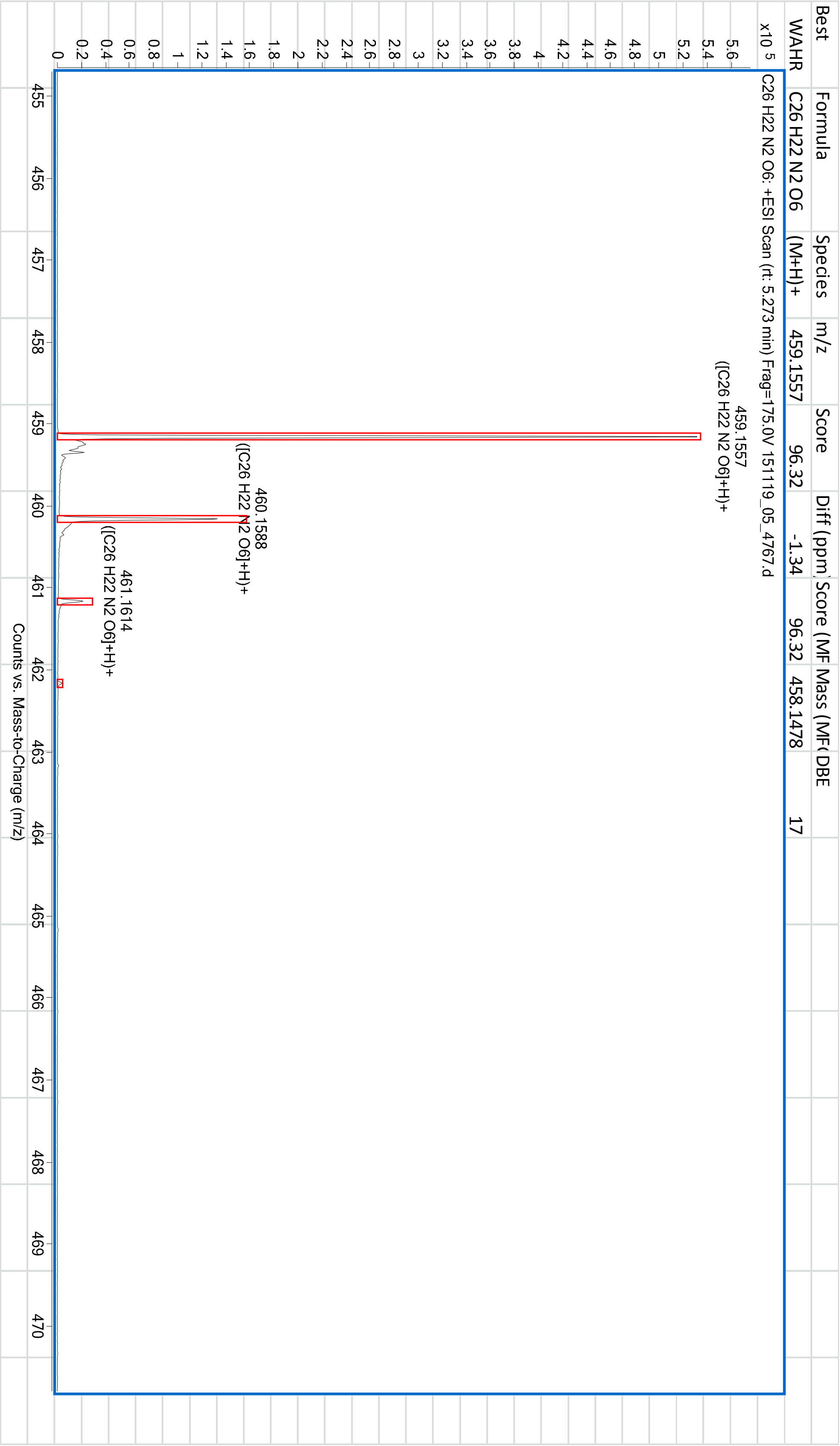

4-(3-{[(2-benzyl-1,3-dioxo-2,3-dihydro-1H-isoindol-5-yl)carbamoyl]methyl}phenoxy)butanoic acid (**15**; ZHAW4768)

NMR

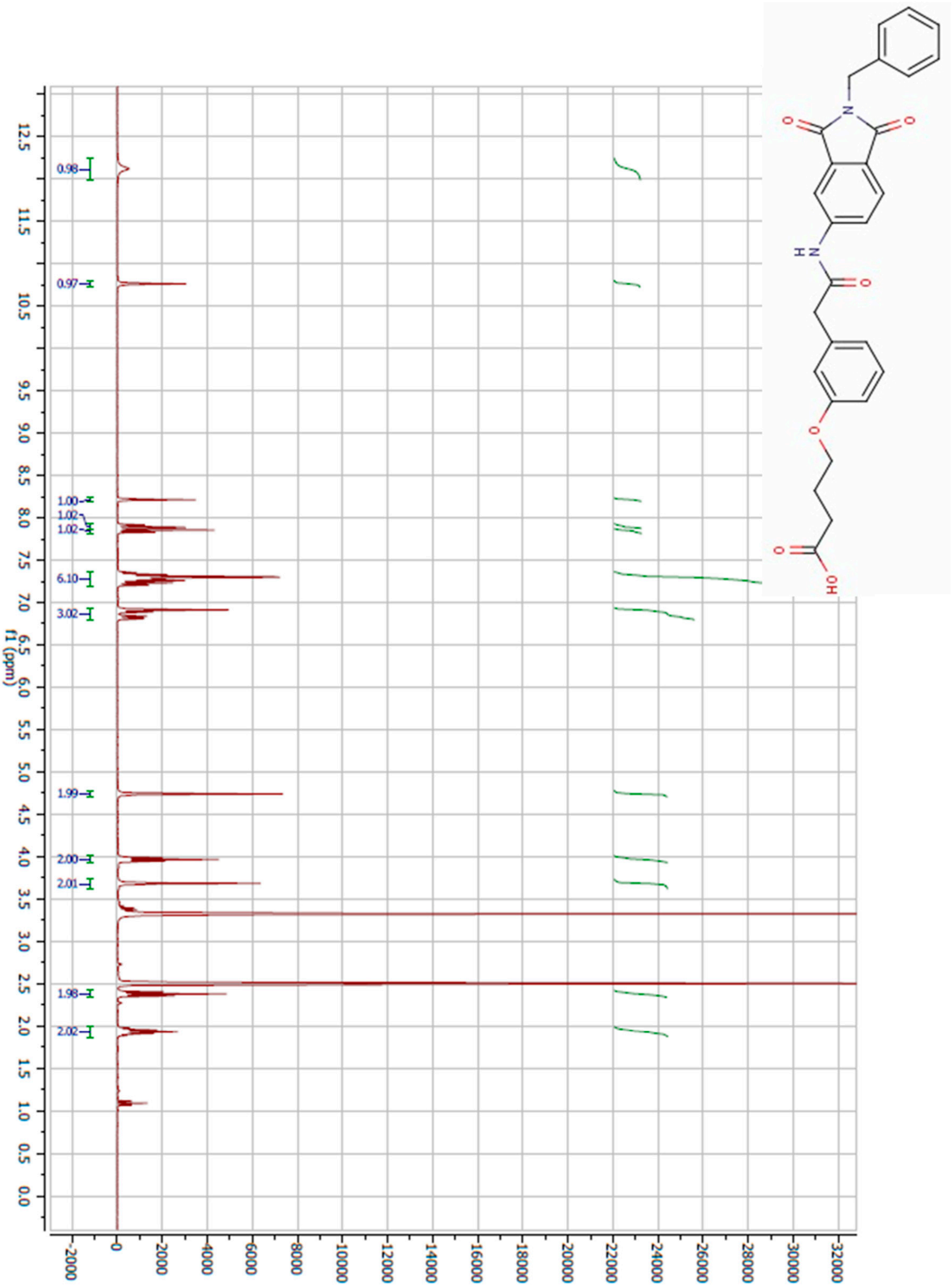

4-(3-{[(2-benzyl-1,3-dioxo-2,3-dihydro-1H-isoindol-5-yl)carbamoyl]methyl}phenoxy)butanoic acid (**15**; ZHAW4768)

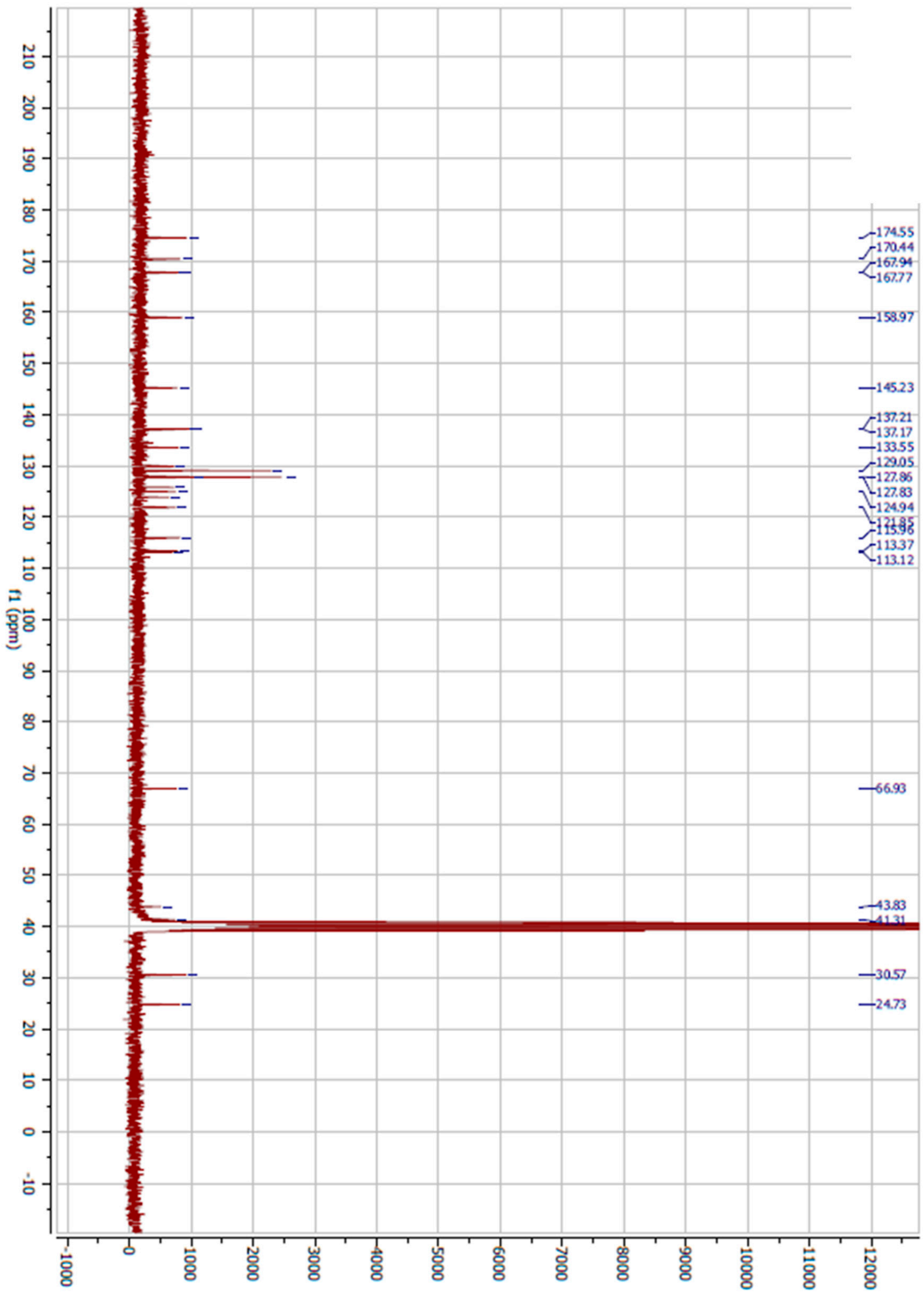

HRMS

4-(3-{[(2-benzyl-1,3-dioxo-2,3-dihydro-1H-isoindol-5-yl)carbamoyl]methyl}phenoxy)butanoic acid (**15**; ZHAW4768)

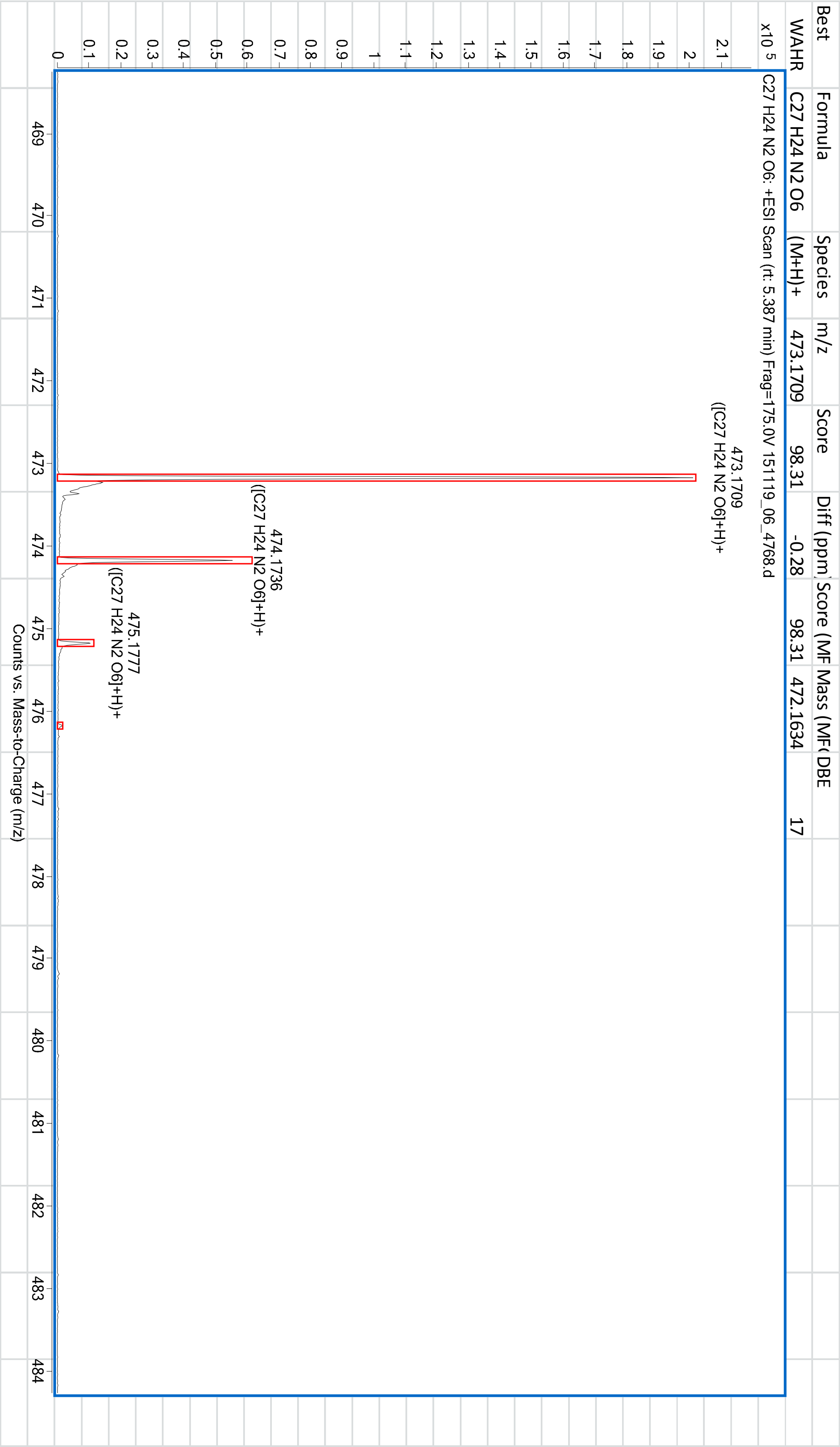

<u>IC50</u>

4-(3-{[(2-benzyl-1,3-dioxo-2,3-dihydro-1H-isoindol-5-yl)carbamoyl]methyl}phenoxy)butanoic acid (**15**; ZHAW4768)

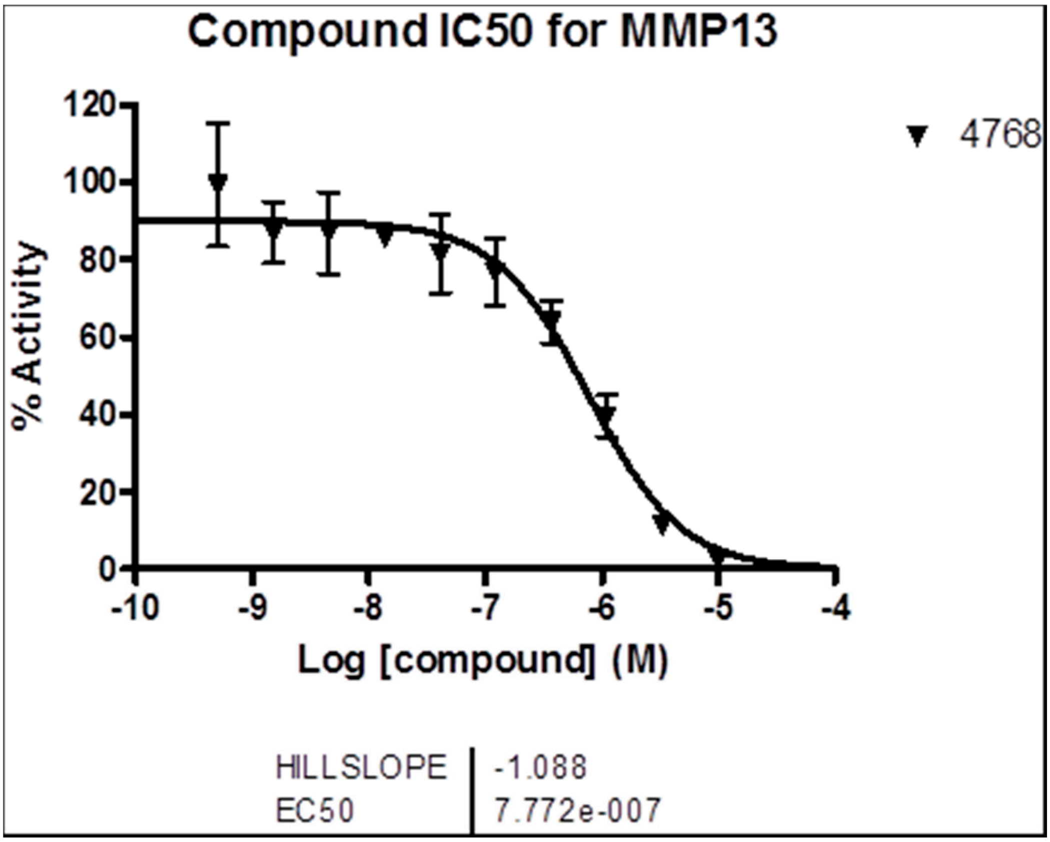

6-(3-{[(2-benzyl-1,3-dioxo-2,3-dihydro-1H-isoindol-5-yl)carbamoyl]methyl}phenoxy)hexanoic acid (**16**; ZHAW5078)

NMR

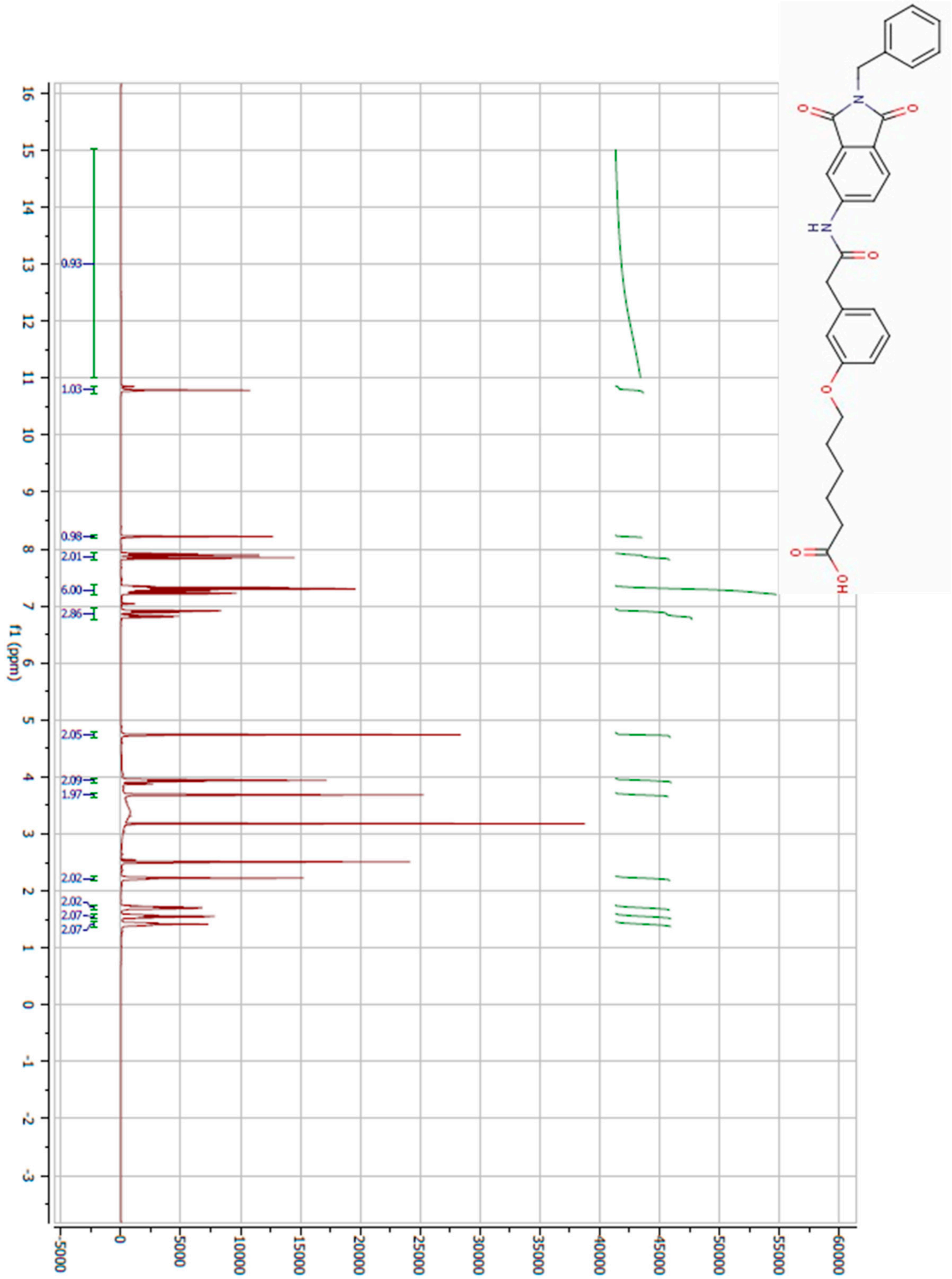

6-(3-{[(2-benzyl-1,3-dioxo-2,3-dihydro-1H-isoindol-5-yl)carbamoyl]methyl}phenoxy)hexanoic acid (**16**; ZHAW5078)

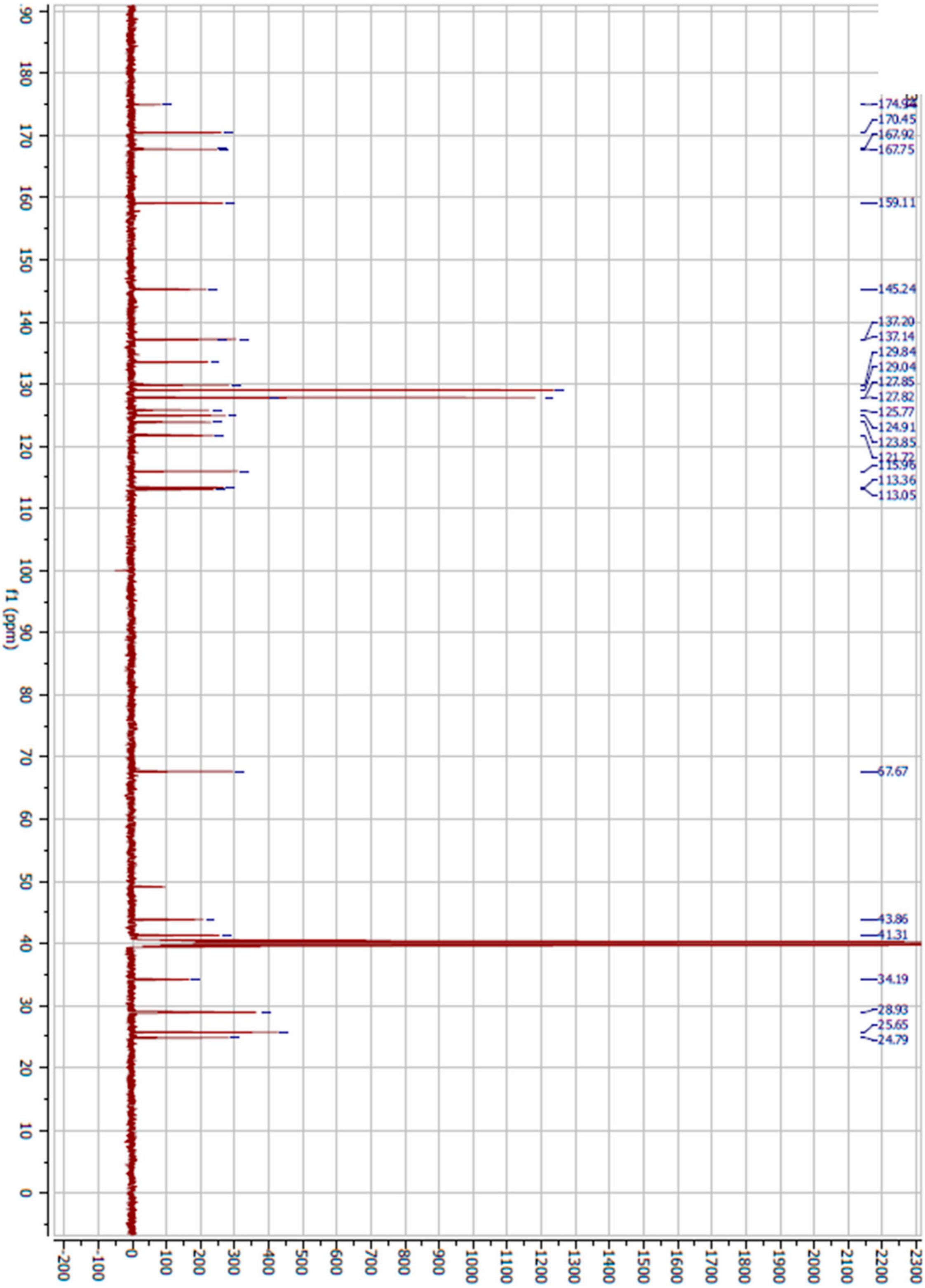

HRMS

6-(3-{[(2-benzyl-1,3-dioxo-2,3-dihydro-1H-isoindol-5-yl)carbamoyl]methyl}phenoxy)hexanoic acid (**16**; ZHAW5078)

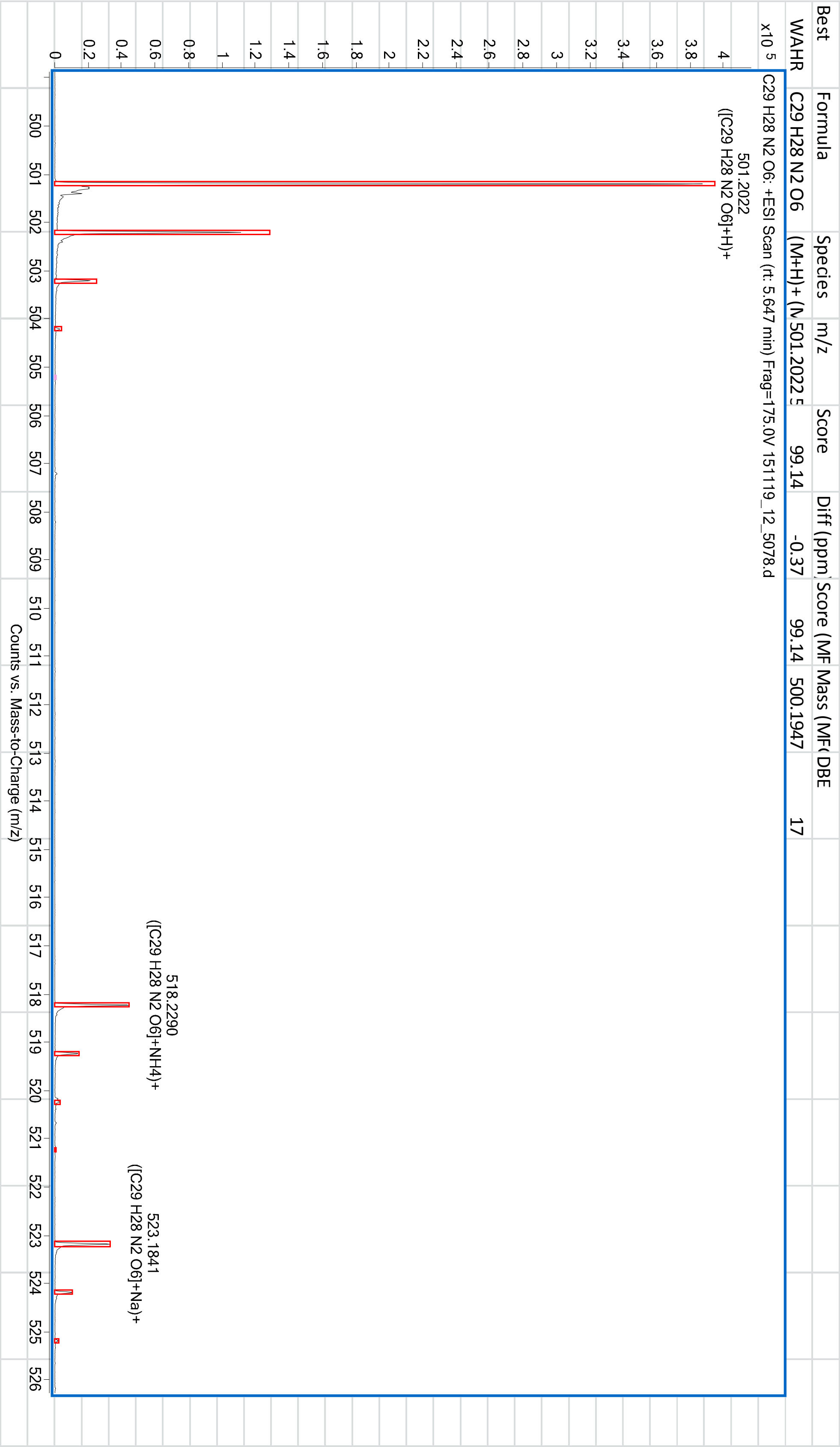

IC$_{50}$

6-(3-{[(2-benzyl-1,3-dioxo-2,3-dihydro-1H-isoindol-5-yl)carbamoyl]methyl}phenoxy)hexanoic acid (**16**; ZHAW5078)

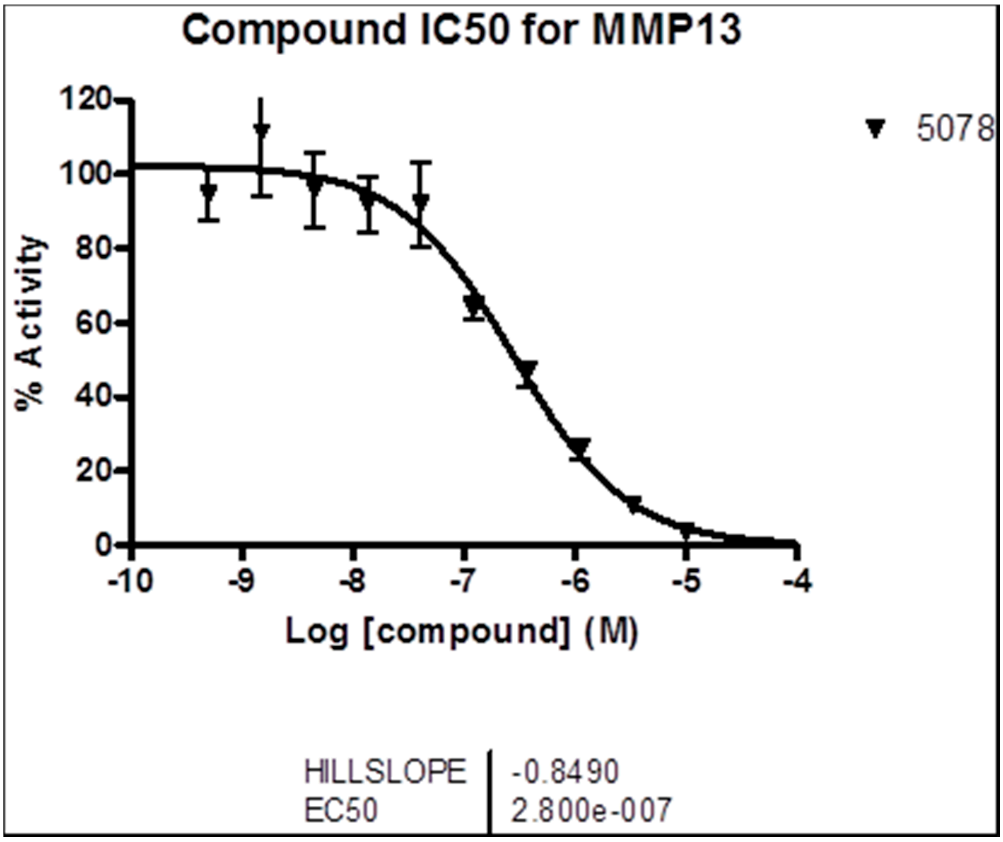

3-(3-{[(2-benzyl-1,3-dioxo-2,3-dihydro-1H-isoindol-5-yl)carbamoyl]methyl}phenoxy)-N-methanesulfonylpropanamide (**17**; ZHAW5136)

NMR

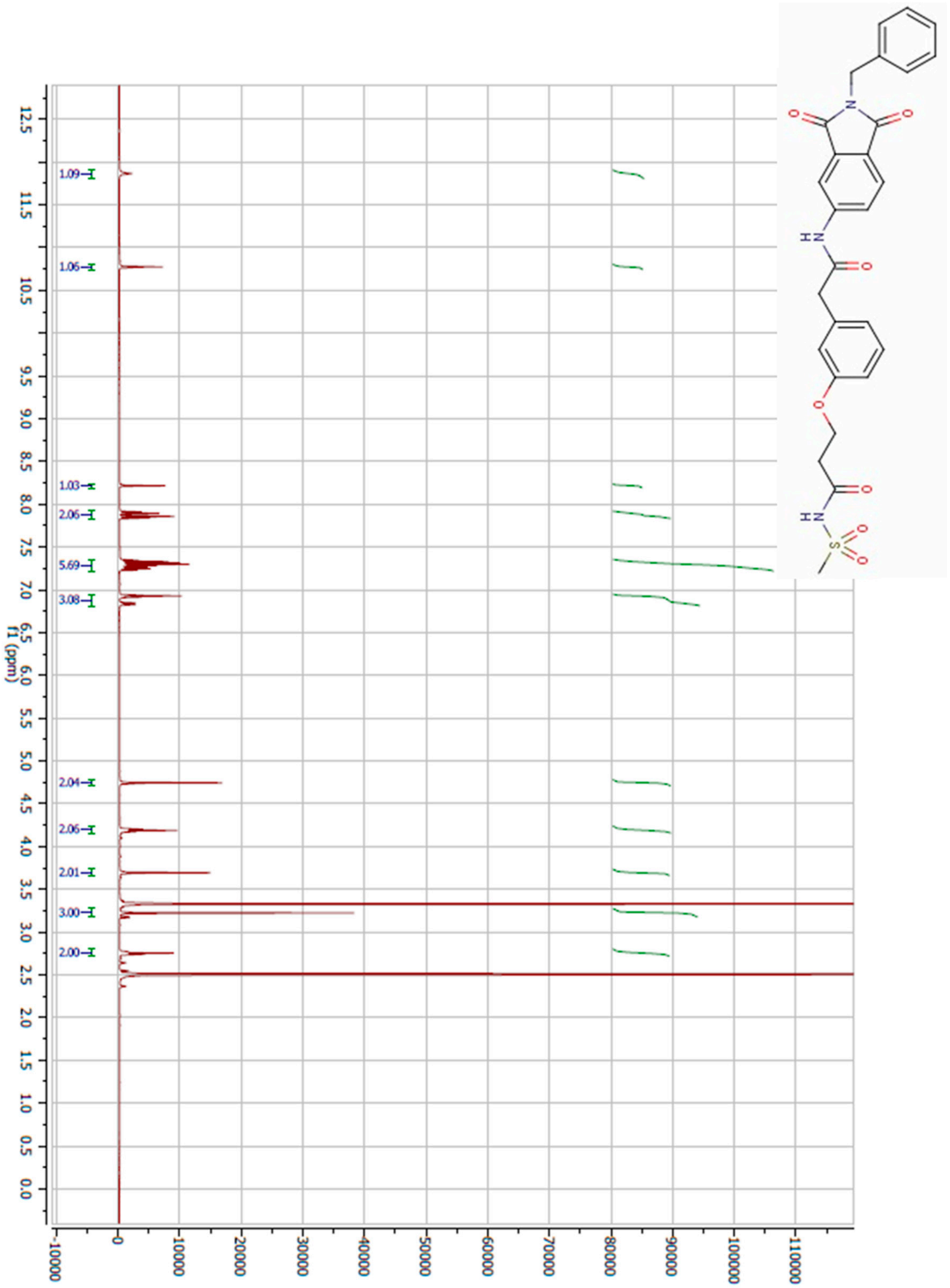

3-(3-{[(2-benzyl-1,3-dioxo-2,3-dihydro-1H-isoindol-5-yl)carbamoyl]methyl}phenoxy)-N-methanesulfonylpropanamide (**17**; ZHAW5136)

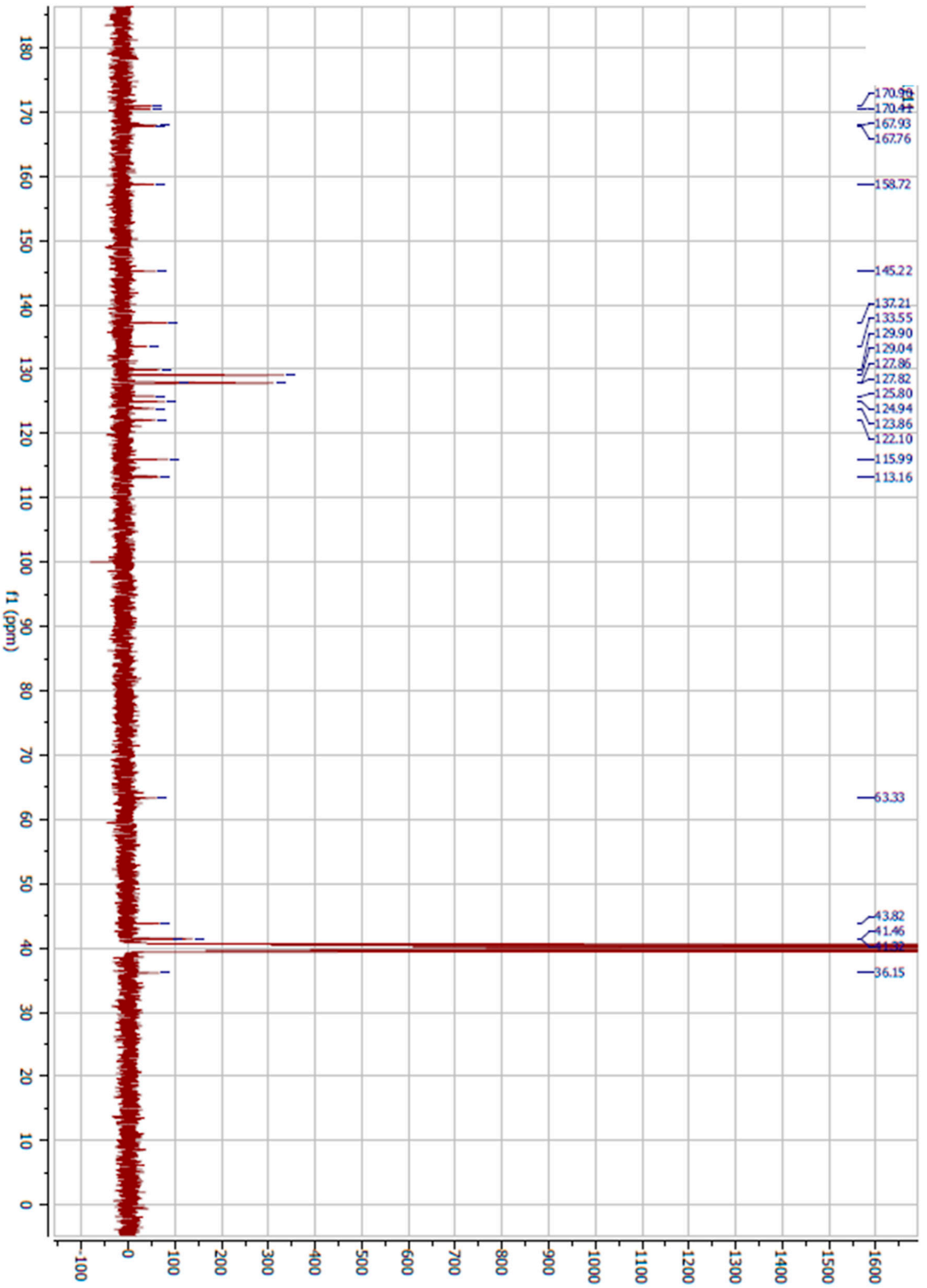

HRMS

3-(3-{[(2-benzyl-1,3-dioxo-2,3-dihydro-1H-isoindol-5-yl)carbamoyl]methyl}phenoxy)-N-methanesulfonylpropanamide (**17**; ZHAW5136)

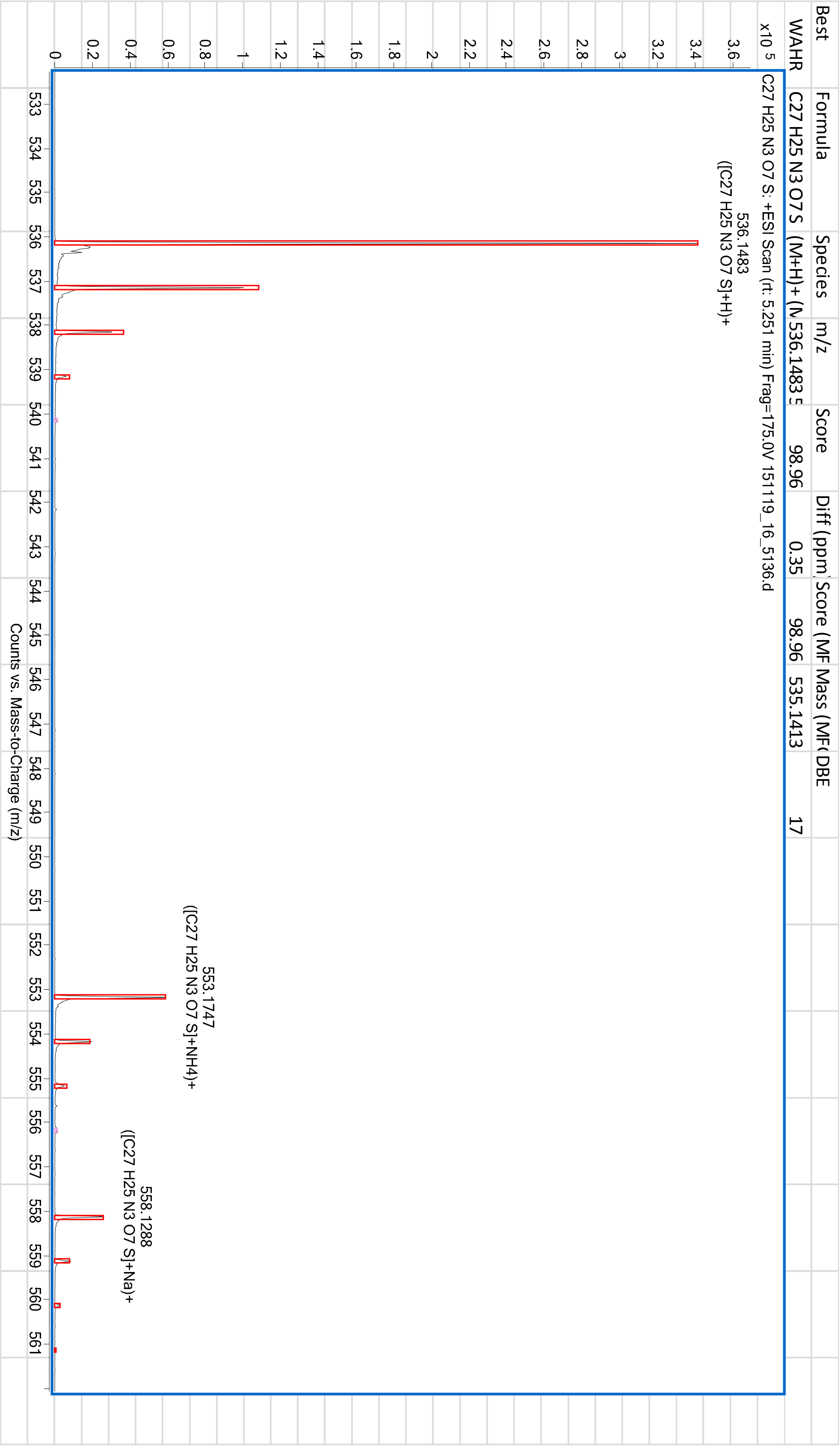

4-(3-{[(2-benzyl-1,3-dioxo-2,3-dihydro-1H-isoindol-5-yl)carbamoyl]methyl}phenoxy)-N-methanesulfonylbutanamide (**18**; ZHAW5135)

NMR

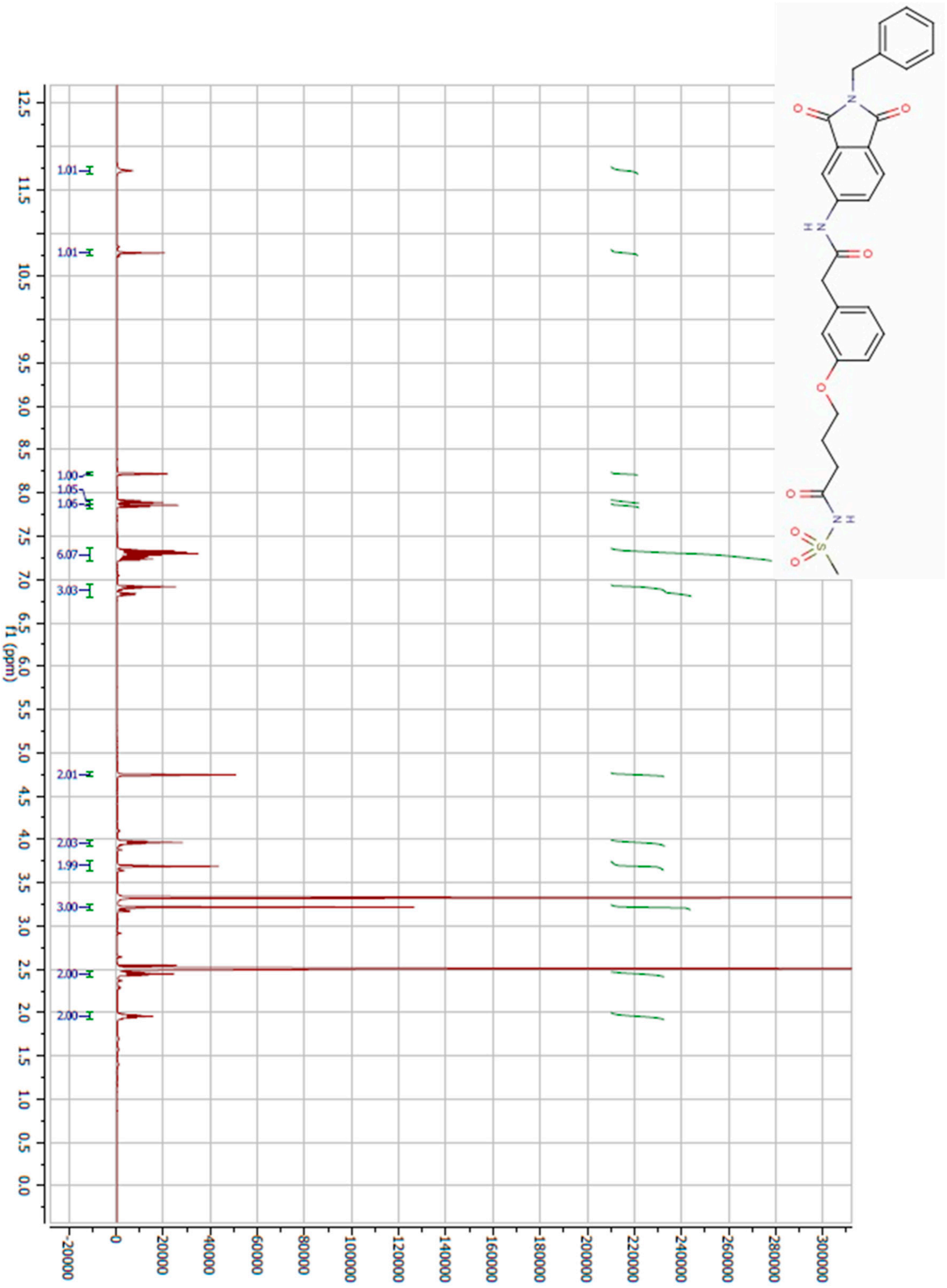

4-(3-{[(2-benzyl-1,3-dioxo-2,3-dihydro-1H-isoindol-5-yl)carbamoyl]methyl}phenoxy)-N-methanesulfonylbutanamide (**18**; ZHAW5135)

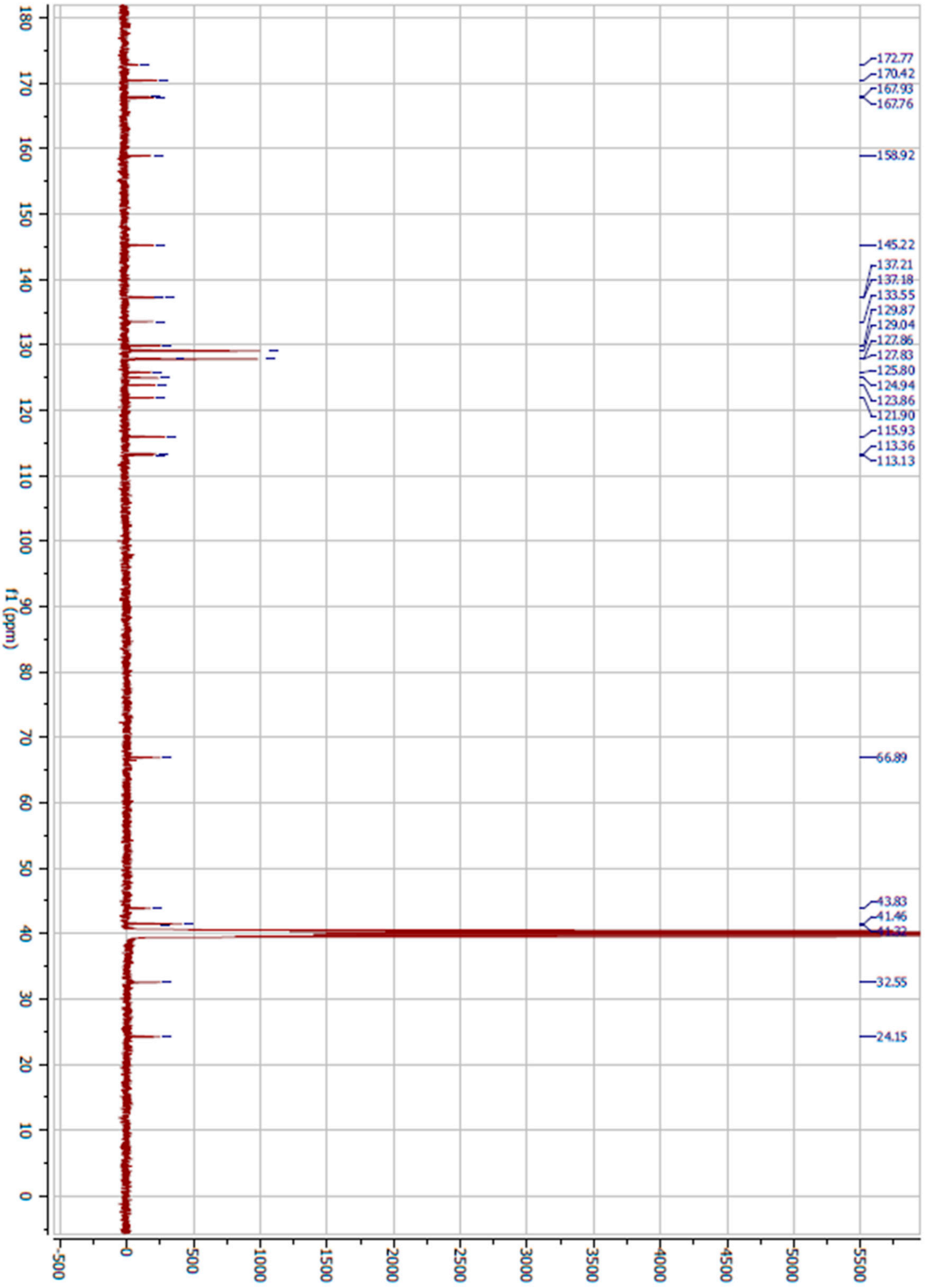

HRMS

4-(3-{[(2-benzyl-1,3-dioxo-2,3-dihydro-1H-isoindol-5-yl)carbamoyl]methyl}phenoxy)-N-methanesulfonylbutanamide (**18**; ZHAW5135)

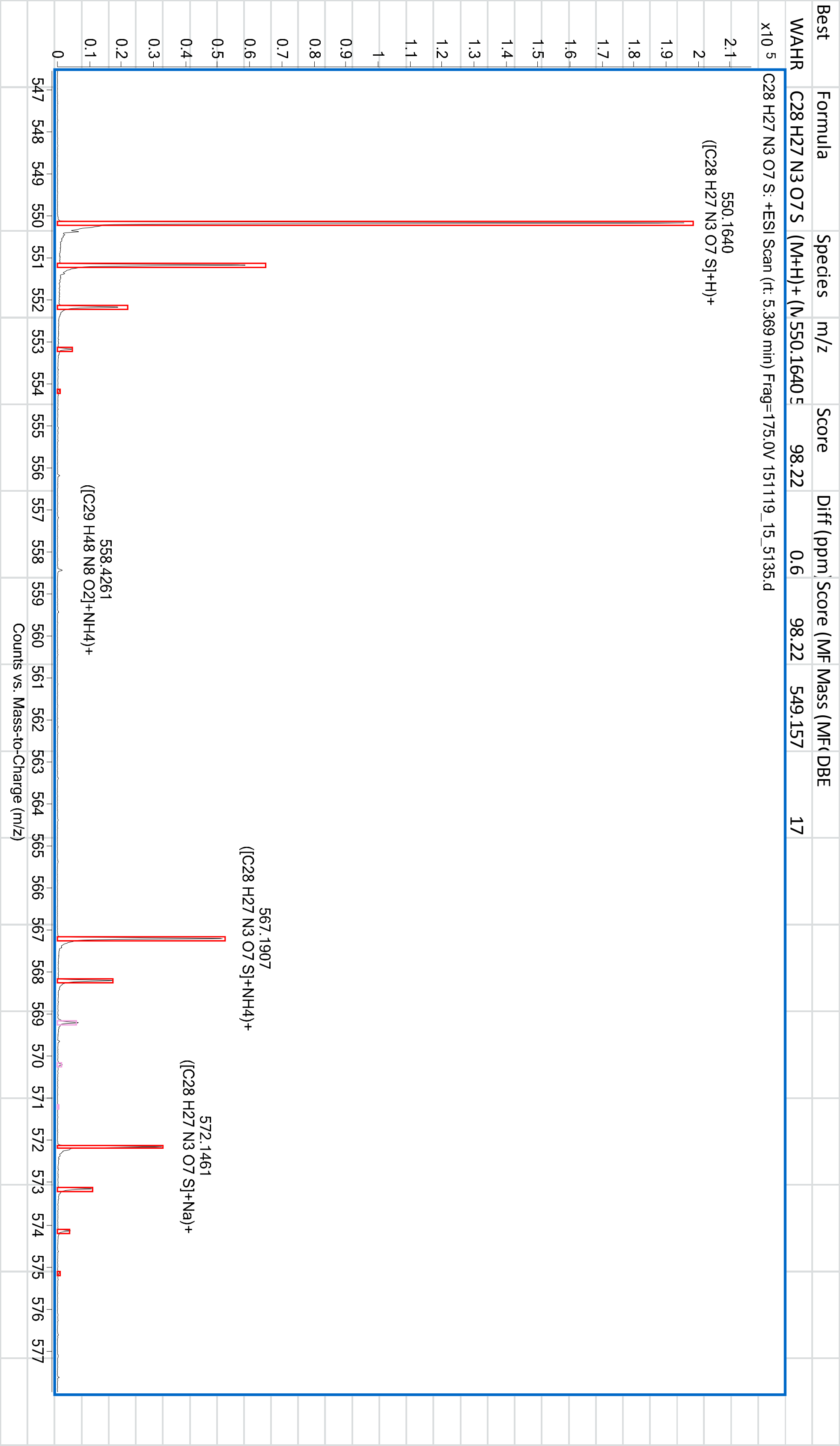

IC$_{50}$

4-(3-{[(2-benzyl-1,3-dioxo-2,3-dihydro-1H-isoindol-5-yl)carbamoyl]methyl}phenoxy)-N-methanesulfonylbutanamide (**18**; ZHAW5135)

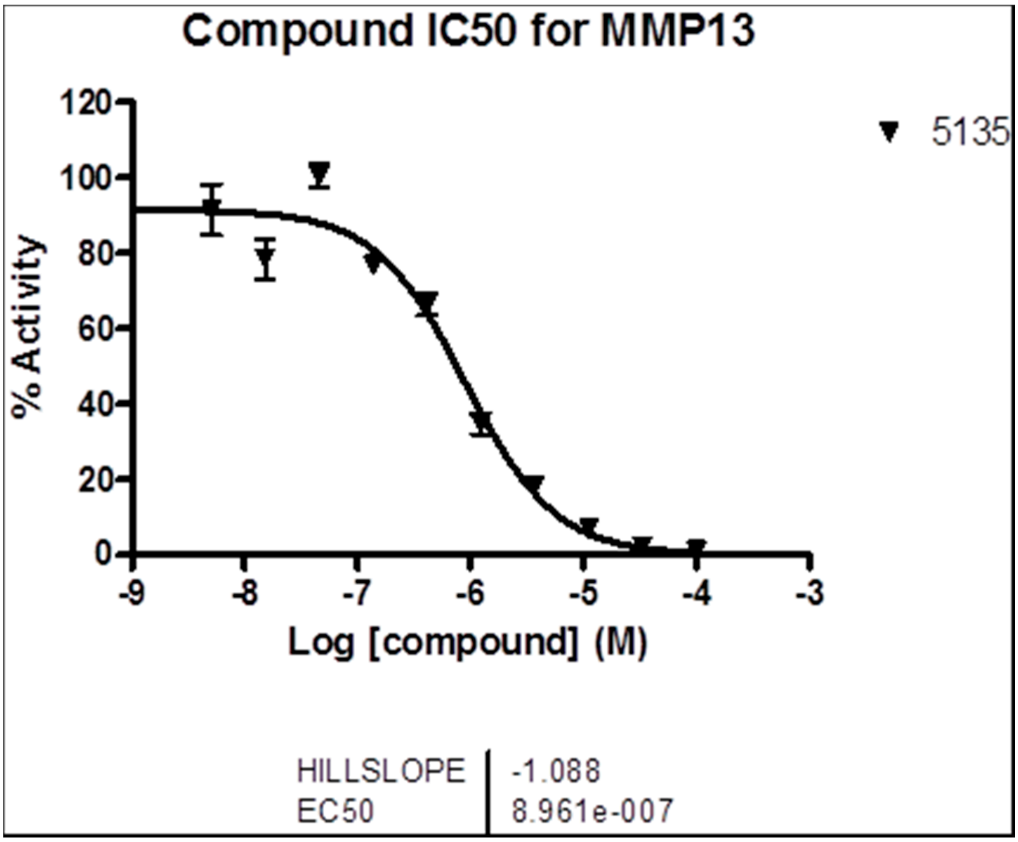

Supplement: Supplementary file 1 [file ijms-17-00314-s001.pdf]
